# Chemoselectivity in the cationic Phospha-Wittig reaction: accessing phosphorus heterocycles, phosphaalkenes, and their annulated [4 + 2] dimers

**DOI:** 10.1039/d5sc08693k

**Published:** 2025-12-26

**Authors:** Philipp Royla, Kai Schwedtmann, Rosa M. Gomila, Antonio Frontera, Jan J. Weigand

**Affiliations:** a Faculty of Chemistry and Food Chemistry, Technische Universität Dresden 01062 Dresden Germany jan.weigand@tu-dresden; b Department of Chemistry, Universitat de Illes Balears 07122 Palma de Mallorca Spain

## Abstract

Triflate salts of phosphito-phosphanides [L_C_P–P(OR)_3_]^+^ (1[OTf], R = alkyl, L_C_ = *N*-heterocyclic carbene) were obtained *via* nucleophilic fragmentation of the tetraphosphetane [(L_C_)_4_P_4_][OTf]_4_ (3[OTf]_4_) with organophosphites P(OR)_3_. The salts 1[OTf] act as versatile reagents in the cationic Phospha-Wittig reaction, converting aldehydes into imidazoliumyl-substituted phosphaalkenes 2[OTf] and, *via* a competing pathway, into diphosphiranes 4[OTf]_2_. The product distribution is governed by the aldehyde substituent, enabling selective access to isolable derivatives of both compound classes. The resulting phosphaalkenes 2[OTf] serve as precursors to diverse phosphorus heterocycles, undergoing expected [2 + 2] dimerisation to 1,3-diphosphetanes *syn*/*anti*-(2)_2_[OTf]_2_ and trapping reactions with 1,3-dienes to yield the tetrahydrophosphinine 8[OTf] and bicyclic derivative 9[OTf]. Most notably, an unprecedented annulative [4 + 2] dimerisation pathway for cationic *C*-aryl phosphaalkenes is uncovered that furnishes benzannulated tetrahydro-1,2-diphosphinines 7[OTf]_2_. Computational studies reveal that the operative mechanism of this transformation involves a Phospha-Diels–Alder step followed by an acid–base-catalytic proton transfer, which is calculated to be energetically more accessible than the classical [2 + 2] dimerisation.

## Abstract 2

We introduce the first cationic Phospha-Wittig reaction to access cationic phosphaalkenes that exhibit charge-enabled cycloaddition reactivity, including an unprecedented annulative [4 + 2] dimerisation.

## Graphical abstract

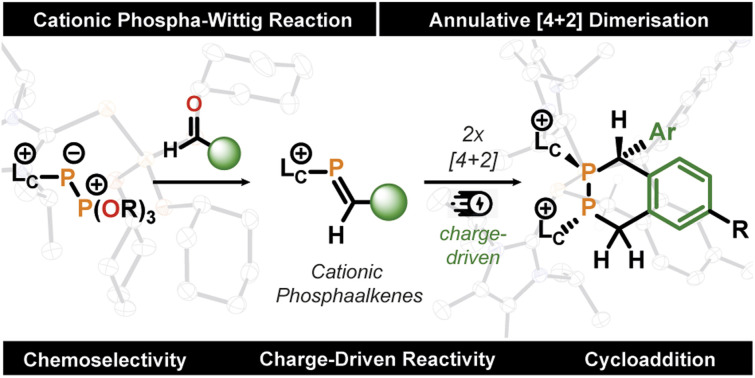

## Introduction

Phosphaalkenes are defined by the presence of a localized phosphorus–carbon double bond (P=C). Despite the electronegativity difference between the two elements, the 2p-3p π-bond in these molecules is nearly apolar,^1^ imparting phosphaalkenes with reactivity patterns more closely resembling those of alkenes than imines.^2,3^ A small HOMO–LUMO gap in phosphaalkenes confers a unique electronic structure that makes them attractive as scaffolds for transition metal-mediated catalysis and as versatile building blocks in polymer synthesis and organophosphorus chemistry.^2,4–6^

**Fig. 1:**
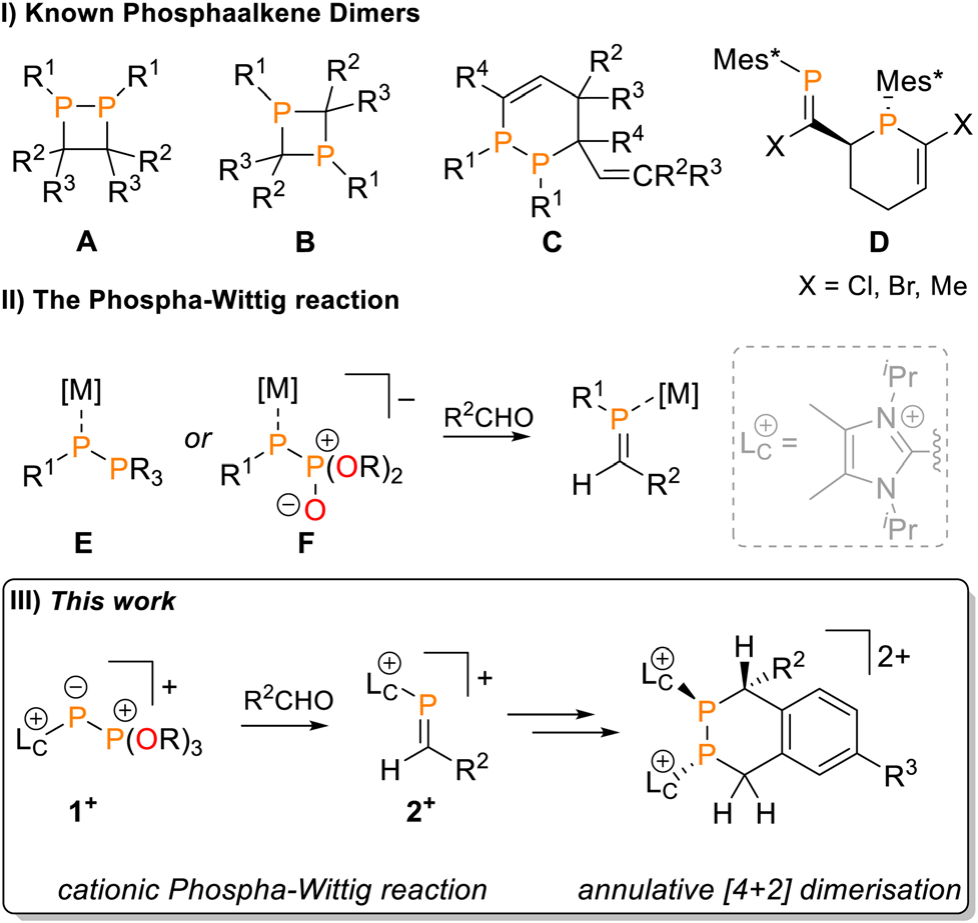
(I) Known examples of phosphaalkenes and 1-phosphabutadiene dimers; (II) general formula for the Phospha-Wittig reaction, [M] = *e.g.* [W(CO)_5_]; (III) synthesis of imidazoliumyl-substituted phosphaalkenes 2^+^ from phosphito-phosphanides 1^+^ and their annulative [4 + 2] dimerisation.

In the absence of sufficient electronic or steric stabilisation, the P=C double bond engages in dimerisation reactions (Fig. 1). The most commonly observed pathway is a [2 + 2] cycloaddition, occurring either in a head-to-head or head-to-tail fashion to furnish 1,2- or 1,3-diphosphetanes A or B, respectively.^7–11^ Alternative dimerisation modes are reported for conjugated 1-phosphabutadienes, which can behave as both dienes and dienophiles in [4 + 2] Phospha-Diels–Alder reactions. Depending on the phosphorus substituent, distinct regioisomeric outcomes are observed. Sterically small substituents favour P–P bond formation to give diphosphacyclohexenes C (R^1^ = Me, Cy, ^*t*^Bu, Ph),^12,13^ whereas the bulky Mes* (Mes* = 2,4,6-^*t*^Bu_3_C_6_H_2_) group promotes formation of phosphaalkene-tethered phosphacyclohexenes D.^14^

Synthetic access to the P=C motif is achieved by approaches that mirror classical organic transformations used to construct C=C double bonds.^9^ A historically important method is the Phospha-Wittig reaction, which involves the transfer of a phosphinidene fragment from phosphanylidene-phosphoranes, R^1^P–PR_3_ or R^1^P[M]–PR_3_ (E, M = metal fragment), or -phosphonates, R^1^P–P(O)(OR)_2_ or R^1^P[M]–P(O)(OR)_2_ (F, M = metal fragment) to a carbonyl compound (Fig. 1).^15–18^

We are investigating the chemistry of cationic *P*-imidazoliumyl-substituted phosphaalkenes, accessible *via* an adapted route from the reaction of phosphonio-phosphanides [L_C_P–PR_3_]^+^ (R = aryl, alkyl, L_C_ = *N*-heterocyclic carbene) with thiocarbonyls.^19^ The imidazoliumyl group stabilises the low-coordinated phosphorus environment and simultaneously acts as a leaving group, permitting post-synthetic modification.^20,21^ This modularity could provide a general route to tailor phosphaalkenes for specific electronic or steric environments from a common precursor, avoiding optimization of multi-step procedures. However, extending this approach to prepare C–H-functionalised phosphaalkenes 2^+^ from aldehydes *via* a cationic Phospha-Wittig reaction was challenging.

The Horner–Wadsworth–Emmons (HWE) reaction offers a compelling alternative to the classical Wittig reaction for the construction of C=C double bonds.^22^ This well-established method employs phosphonate-stabilised carbanions, which are more nucleophilic than their ylide counterparts. Indeed, the heavier metal-coordinated phosphorus analogues F (Fig. 1) readily react with ketones, while the reactivity of E is limited to aldehydes. Inspired by this logic, we targeted phosphito-phosphanides 1^+^ and hypothesized that they could react with aldehydes to give access to imidazoliumyl-substituted phosphaalkenes 2^+^.

We now report the synthesis of isolable triflate salts of 1^+^*via* the nucleophilic fragmentation of tetraphosphetane [(L_C_)_4_P_4_][OTf]_4_ (3[OTf]_4_) by organophosphites P(OR)_3_ (R = alkyl). Derivatives of 1^+^ enable the cationic Phospha-Wittig reaction with a range of aldehydes for the first time. We systematically assess the chemoselectivity of this transformation leading to selective access to isolable phosphaalkenes and diphosphiranes. In addition, we uncover a hitherto unknown [4 + 2] dimerisation pathway of *C*-aryl substituted phosphaalkenes that furnishes benzannulated tetrahydro-1,2-diphosphinines. Computational studies reveal an underlying Phospha-Diels–Alder mechanism succeeded by a proton shift mediated by acid-base catalysis. Crucially, our findings highlight that cationically charged *P*-imidazoliumyl-substituted phosphaalkenes unlock new reactivity profiles that are inaccessible to their neutral counterparts.

## Results and discussion

### Synthesis of phosphito-phosphanides

**Scheme 1:**
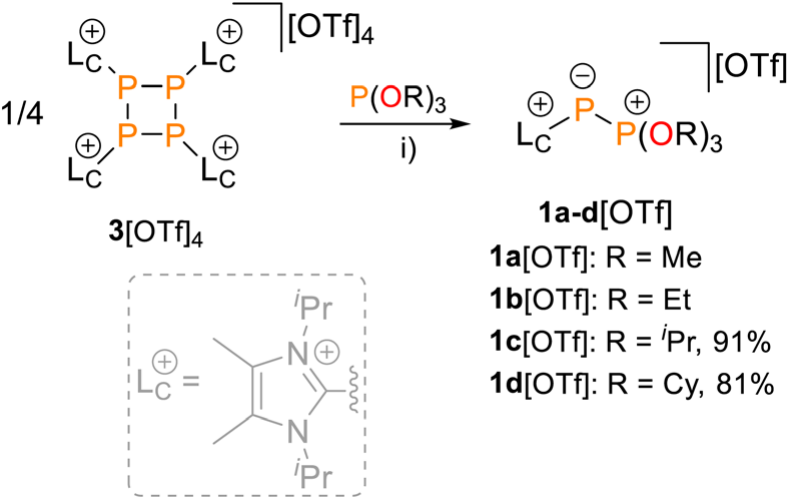
Nucleophilic fragmentation of 3[OTf]_4_ with organophosphites P(OR)_3_ (R = Me, Et, ^*i*^Pr, Cy); reagents and conditions: (i) +4.2 P(OR)_3_, CH_3_CN or CD_2_Cl_2_, r.t., 16 h, 91% (1c[OTf], R = ^*i*^Pr), 16 h, 81% (1d[OTf], R = Cy).

Treating 3[OTf]_4_ with a slight excess (4.2 equiv.) of organophosphites P(OR)_3_ (R = Me, Et, ^*i*^Pr, Cy) in CH_3_CN or CD_2_Cl_2_ gave pale-yellow solutions after 16 h at room temperature (Scheme 1). In all cases, ^31^P NMR spectroscopic investigations of aliquots removed from the reaction mixture revealed the complete conversion of 3[OTf]_4_. The products displayed the expected AX spin systems with characteristically shielded resonances of the A parts and were assigned to 1a–d[OTf] [*δ*(^31^P_A_) = −199.4–(–182.4) ppm, *δ*(^31^P_X_) = 78.3–90.2 ppm, ^1^*J*(PP) = −608–(–601) Hz; see the SI for details, Section S2.1].^19,23^

**Fig. 2:**
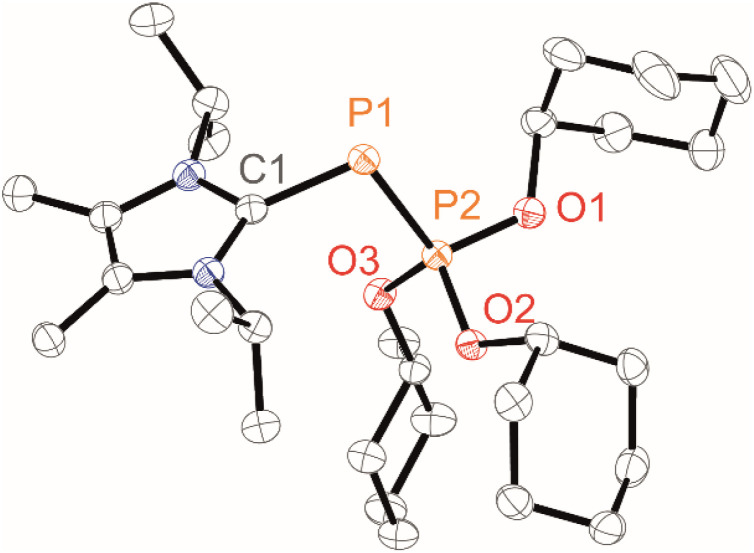
Molecular structure of 1d^+^ in 1d[OTf]; hydrogen atoms are omitted for clarity, and thermal ellipsoids are displayed at 50% probability (100 K); selected bond lengths (in Å) and angles (in °): P1–P2 2.0832(5), C1–P1 1.8151(16), P2–O1 1.5713(12), P2–O2 1.5764(11), P2–O3 1.5694(11), C1–P1–P2 96.75(5).

Whereas work-up of 1a,b[OTf] led to their decomposition to unidentified products and starting material (see the SI, Fig. S1), analytically pure 1c,d[OTf] were obtained in isolated yields of 91% and 81%, respectively. Their synthesis is conveniently adaptable to a multigram scale (∼3.5 g). Structural confirmation of 1d[OTf] was achieved through single-crystal X-ray diffraction analysis after recrystallisation from a saturated C_6_H_5_F/Et_2_O solution at room temperature (Fig. 2). The observed P–P bond length [2.0832(5) Å] is markedly shorter than those reported for cationic phosphonio-phosphanides [L_C_P–PR_3_]^+^ [R = alkyl, 2.1162(4)–2.1446(4) Å]^19^ and falls at the lower end of the range for structurally characterised phosphanylidene-phosphoranes (2.06–2.15 Å),^23^ approaching values characteristic of P=P double bonds (*cf.* P=P 2.04 Å, P–P 2.22 Å).^24,25^

### Phosphito-phosphanides as Phospha-Wittig reagents

**Scheme 2:**
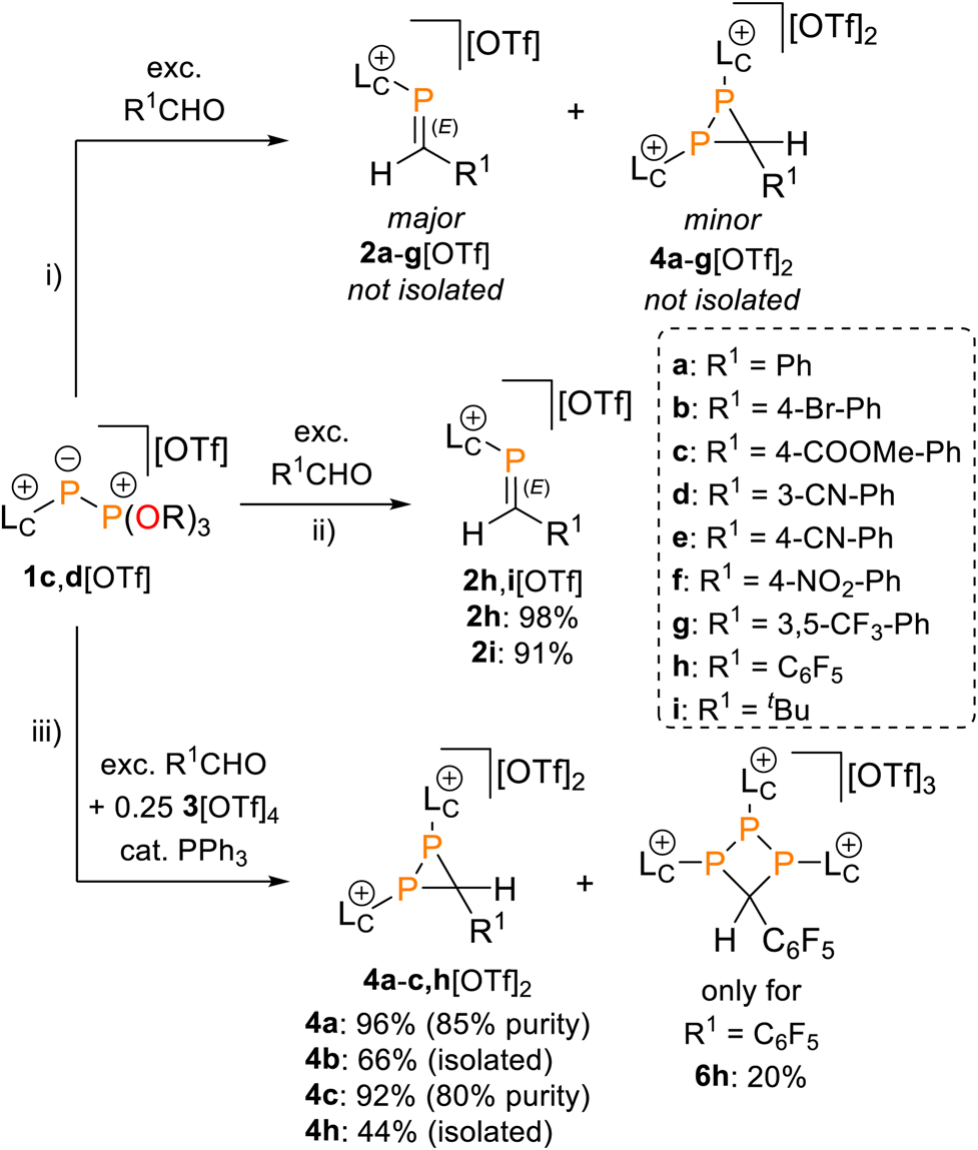
Reaction of 1c,d[OTf] with an excess of aldehydes R^1^CHO to give inseparable mixtures of 2a–g[OTf] and 4a–g[OTf]_2_ (top) or isolable 2h,i[OTf] (middle); and direct, independent synthesis of 4a–c[OTf]_2_ (bottom); reagents and conditions: (i) + 2.0–5.0 equiv. of R^1^CHO, – OP(OR)_3_, CH_3_CN, r.t., 4 h-7 d, 98% (for 2h[OTf]), 91% (for 2i[OTf]); (ii) for R^1^ = C_6_F_5_: +2.0 equiv. of F_5_C_6_CHO, −OP(O^*i*^Pr)_3_, CH_3_CN, r.t., 4 h, 98%; for R^1^ = ^*t*^Bu: +10.0 equiv. of ^*t*^BuCHO, −OP(O^*i*^Pr)_3_, CH_3_CN, r.t., 4 d, 91%; (iii) for 4a–c[OTf]_2_: +5.0 equiv. of R^1^CHO, +0.25 equiv. of 3[OTf]_4_, +0.1 equiv. of Ph_3_P, −OP(O^*i*^Pr)_3_, CH_3_CN, r.t., 4 d-7 d, 66% (for 4b[OTf]_2_), for 4h[OTf]_2_ and 6h[OTf]_3_: +2.0 equiv. of F_5_C_6_CHO, +0.25 equiv. of 3[OTf]_4_, +0.1 equiv. of Ph_3_P, −OP(O^*i*^Pr)_3_, CH_3_CN, r.t., 3 d, 44% (for 4h[OTf]_2_) and 20% (for 6h[OTf]_3_).

The reactivity of 1c,d[OTf] was tested in reactions with an excess (2–10 equiv.) of a series of aldehydes R^1^CHO in CH_3_CN solution (Scheme 2). ^31^P NMR spectroscopic analysis of aliquots removed from the reaction mixtures after 4 h to 7 d (see Table 1) showed successful Phospha-Wittig conversion to the phosphoric acid esters, OP(O^*i*^Pr)_3_ [*δ*(^31^P) = −2.8 ppm]^26^ or OP(OCy)_3_ [*δ*(^31^P) = −2.5 ppm], and phosphaalkenes *E*-2a–i^+^, indicated by diagnostic, deshielded resonances [*δ*(^31^P) = 173.8–200.5 ppm, ^2^*J*(PH) = 14–23 Hz, see the SI, Fig. S13]. Minor, slightly upfield-shifted [*δ*(^31^P) = 154.0–176.6 ppm] resonances were assigned to the corresponding *Z*-isomers due to the characteristically large ^2^*J*(PH) coupling constant [^2^*J*(PH) = 43–44 Hz].^27^ The reactions are highly stereoselective with typical *E*/*Z* ratios > 97 : 3. The ^31^P NMR resonances of phosphaalkenes 2a–i^+^ are shifted to lower frequencies compared to the reported values of neutral C–H substituted phosphaalkenes [*δ*(^31^P) = avg. 250 ppm].^8,16,18,28,29^ Similar trends are known for inversely polarized phosphaalkenes.^30^

**Table 1:** Scope of the Phospha-Wittig reaction of 1c,d[OTf] with a series of aldehydes

R^1^ =		Reagent	Time^a^	Equiv.	2^+^ : 4^2+^ ratio	*δ*(^31^P):*E-*2^+^, *Z*-2^+^ in ppm	^2^ *J*(PH):*E-*2^+^, *Z*-2^+^ in Hz
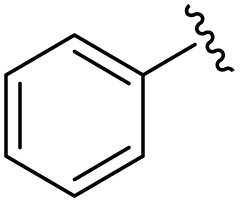	2a^+^	1c[OTf]	7d	5.0	63 : 37	178.2, 154.0	19, 44
		1d[OTf]	7d	5.0	65 : 35		
		1c[OTf]	4h^b^	5.0	42 : 57		
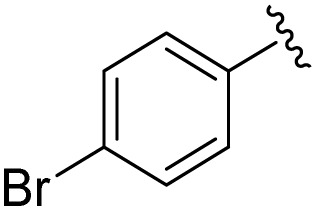	2b^+^	1c[OTf]	5d	5.0	79 : 21	182.5, 157.8	21, 43
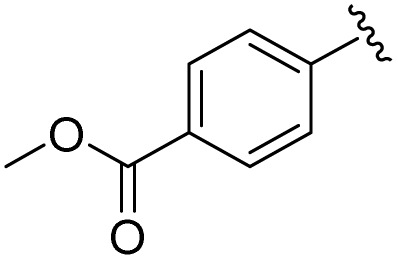	2c^+^	1c[OTf]	4d	5.0	83 : 17	191.5, 167.0	22, 44
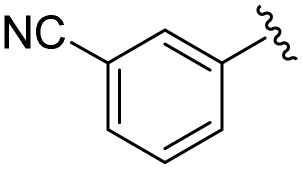	2d^+^	1c[OTf]	4d	5.0	91 : 9	192.0, 168.4	22, 43
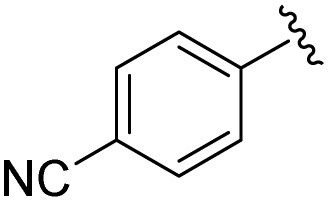	2e^+^	1c[OTf]	16h	5.0	91 : 9	197.5, 172.3	20, 43
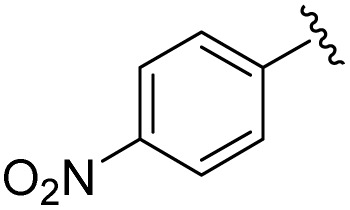	2f^+^	1c[OTf]	16h	5.0	92 : 8	200.5, 175.5	22, 43
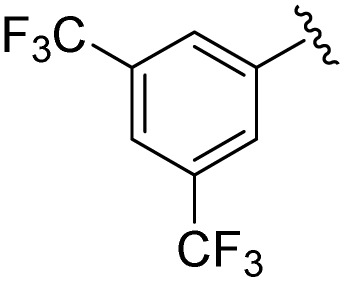	2g^+^	1c[OTf]	16h	5.0	94 : 6	200.3, 176.6	23, 43
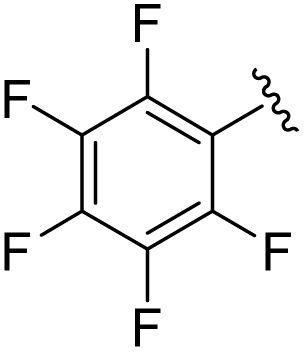	2h^+^	1c[OTf]	4h	2.0	>99 : 1	215.5, –	20, –
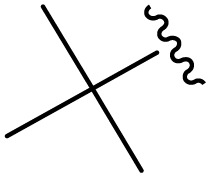	2i^+^	1c[OTf]	4d	10.0	>99 : 1	173.8, –	14, –

a Time required for full conversion of 1c,d[OTf] at room temperature, if not specified differently.

b 80 °C.

In addition to phosphaalkenes, the ^31^P{^1^H} NMR spectra of most reaction mixtures (for 2a–g^+^) showed sets of shielded resonances of AX spin systems [*e.g.*, R^1^ = Ph (a): *δ*(^31^P_A_) = −174.1 ppm, *δ*(^31^P_X_) = −138.9 ppm, ^1^*J*(PP) = 128 Hz, ^2^*J*(P_X_H) = 31 Hz] in line with the formation of diphosphiranes 4a–g^2+^ (Scheme 2, for the integral ratio see Table 1).^31^ Diphosphirane formation was rationalised by cyclopropanation of the *in situ*–formed phosphaalkenes by phosphinidene [L_C_–P]^+^, transferred from 1c,d[OTf], in a ligand displacement reaction.^19,32,33^ Supporting evidence includes ^31^P NMR resonances consistent with the transient formation of free P(O^*i*^Pr)_3_ [*δ*(^31^P) = 140.7 ppm] over the course of the conversion (see the SI, Fig. S14). In the presence of excess aldehyde, released P(O^*i*^Pr)_3_ is consumed in an Abramov reaction^34^ to produce diisopropyl (isopropoxy(aryl)methyl)phosphonates (^*i*^PrO)_2_OP–C(Ar)O^*i*^Pr [*δ*(^31^P) = *ca.* 25.0 ppm; *cf.* (MeO)_2_OP–C(Ph)OMe: *δ*(^31^P) = 21.9 ppm].^35^ Conversion rates of the Phospha-Wittig reaction were accelerated by elevated temperatures, increasing diphosphirane formation, albeit at the expense of lower chemoselectivity, and the formation of several other unidentified products (see the SI, Section S2.6). Notably, while diphosphiranes are typically not observed in the Phospha-Wittig reaction using phosphanylidene-phosphoranes, Gates *et al.* reported diphosphirane formation in the Phospha–Peterson reaction.^8^

**Fig. 3:**
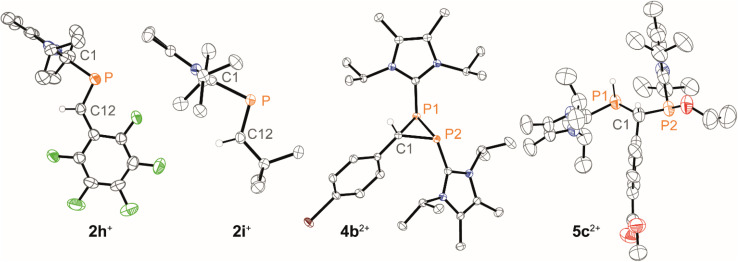
Molecular structures of 2h,i^+^ in 2h,i[OTf], 4b^2+^ in 4b[OTf]_2_·*o*-C_6_H_4_F_2_, and 5c^2+^ in 5c[OTf]_2_·*o*-C_6_H_4_F_2_; selected hydrogen atoms and anions are omitted for clarity, and thermal ellipsoids are displayed at 50% probability (100 K); selected bond lengths (Å) and angles (°): for 2h^+^: P1–C12 1.692(4), C1–P1–C12 97.71(19); 2i^+^: P1–C12 1.671(2), C1–P1–C12 101.38(10); for 4b^2+^: P1–P2 2.2270(5), P1–C1 1.8668(13), P2–C1 1.8729(13), P1–C1–P2 73.10(5), C1–P1–P2 53.58(4); for 5c^2+^: P1–C1 avg. 1.916, P2–C1 avg. 1.858, P1–C1–P2 avg. 101.6.

In general, reactions of 1c[OTf] with aldehydes bearing electron-withdrawing groups or ^*t*^BuCHO favoured phosphaalkene formation (see Table 1), while reactions with 4-methoxybenzaldehyde or mesitylaldehyde did not result in satisfactory phosphaalkene or diphosphirane production (see the SI, Section S2.7). Similar solubilities of the triflate salts of phosphaalkenes 2a–g^+^ and diphosphiranes 4a–g^2+^ prevented their separation. However, the phosphaalkenes 2h,i^+^ [R^1^ = C_6_F_5_ (2h^+^), ^*t*^Bu (2i^+^)] were obtained chemoselectively leading to the isolation of their triflate salts in excellent yields of 98% and 91%, respectively. The *E*-configuration of the cations was verified by single-crystal X-ray diffraction analysis (Fig. 3). In the solid state the P=C double bond lengths {2h[OTf]: P1–C12 1.692(4) Å, 2i[OTf]: P1–C12 1.671(2) Å} are similar to other structurally characterised, C–H substituted phosphaalkenes (1.61–1.71 Å),^36^ for instance *E*-Mes*P=C(H)Ph [P=C 1.660(6) Å],^29^ and close to the calculated values for the parent HP=CH_2_ [P=C 1.652 Å (at the 6-31G* level of theory)].^37^ We also note that the P=C bond in 2h[OTf] is co-planar with the C_6_F_5_ substituent, indicating a delocalization of the π-electron density.^9^

The direct and chemoselective formation of the diphosphiranes 4a–c^2+^ was achieved independently by reacting 1c[OTf] with the corresponding aldehydes in the presence of 0.25 equivalents of 3[OTf]_4_ and catalytic PPh_3_, as a source of [L_C_–P]^+^ (Scheme 2). After work-up, the triflate salts 4a,c[OTf]_2_ were obtained as crude solids (purity 80–85%, determined by ^31^P NMR spectroscopy), enabling the unambiguous assignment of all atoms with multinuclear NMR analysis. Efforts to purify these solids by washing with various solvent mixtures, recrystallisation, or separation over a silica plug under inert conditions did not improve purity and in some cases resulted in decomposition to unidentified products. In contrast, diphosphirane 4b[OTf]_2_ precipitated as an analytically pure solid from a saturated C_6_H_5_F solution of the crude product and was isolated in 66% yield.

X-ray diffraction analysis of suitable crystals of 4b[OTf]_2_ verified the molecular structure and the *anti*-disposition of the two L_C_-substituents (Fig. 3). The central three-membered ring features acute bond angles [*e.g.* P1–C1–P2 73.10(5)°] and C–P bond distances [P1–C1 1.8668(13) Å, P2–C1 1.8729(13) Å], consistent with other structurally identified diphosphiranes.^19,31,33,38^ The P1–P2 bond length [P1–P2 2.2270(5) Å] is at the longer end of comparable structures.

Diphosphiranes are known to engage in ring opening reactions *via* P–P bond cleavage upon photo- or thermal treatment or in reactions with electro- and nucleophiles.^39^ Similarly, crude compound 4c[OTf]_2_ activates the O–H bond in EtOH leading to the isolation of 5c[OTf]_2_ in 50% yield, verified by analysis of the molecular structure of 5c[OTf]_2_ by single crystal X-ray diffraction analysis (Fig. 3). The product was isolated as a mixture of two diastereomers and variable temperature NMR experiments showed no interconversion between them in a range of 240–340 K (see the SI, Fig. S32).

**Fig. 4:**
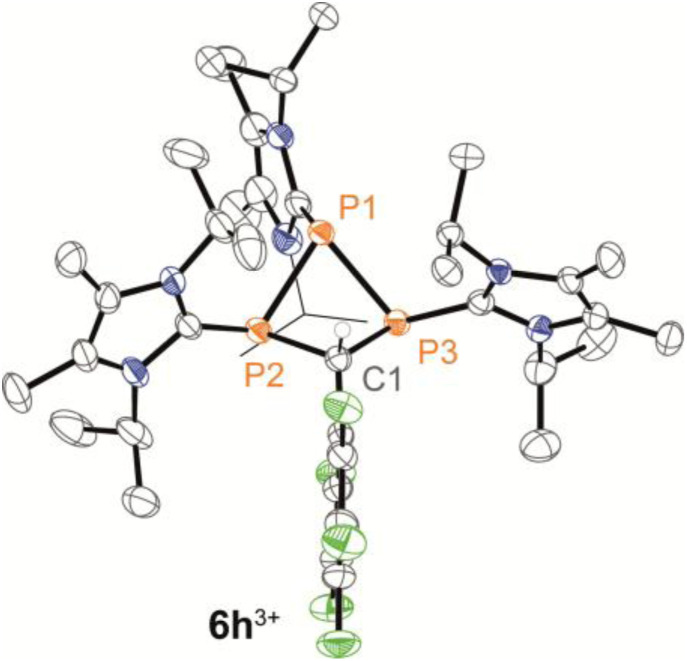
Molecular structures of 6h^3+^ in 6h[OTf]_3_·C_6_H_5_F; selected hydrogen atoms and anions are omitted for clarity, and thermal ellipsoids are displayed at 50% probability (100 K), one ^*i*^Pr of the L_C_ group at P1 is shown in wireframe for clarity; selected bond lengths (Å) and angles (°): for 6h^3+^: P1–P2 2.2354(8), P1–P3 2.2540(9), P2–C1 1.890(2), P3–C1 1.904(3), P2–P1–P3 73.56(3), P2–C1–P3 90.24(10).

We further conducted experiments of the *in situ*-generated or isolated phosphaalkenes 2h,i[OTf] with 0.25 equivalents of 3[OTf]_4_ and catalytic PPh_3_ to access the diphosphiranes 2h,i[OTf]_2_. No signs of conversion were observed for 2i^+^ (R^1^ = ^*t*^Bu) after over a week of stirring at room temperature or treatment at elevated temperatures (up to 70 °C). In contrast, the reaction of phosphaalkene 2h^+^ (R^1^ = C_6_F_5_) afforded diphosphirane 4h^2+^ as the major product. Minor amounts of another previously unobserved product showed sets of resonances in agreement with a AX_2_ spin system in the ^31^P{^19^F} NMR spectrum (see the SI, Sections S2.14 and S2.15), which led to the tentative assignment of the product as triphosphetane 6h[OTf]_3_ (Scheme 2). A crude solid isolated from the mixture contained both products in a 76 : 24 integral ratio. Separation of the products afforded the triflate salts of diphosphirane 4h[OTf]_2_ and triphosphetane 6h[OTf]_3_ as analytically pure solids in 44% and 20% yield, respectively, with only minimal contamination (<5%) of the other product according to multinuclear NMR analysis. Attempts to bias the product distribution to selectively form 6h[OTf]_3_ by performing the reaction with 0.5 equivalents of 3[OTf]_4_ did not lead to a meaningful difference in the observed chemoselectivity (see the SI, Section S2.3). Single crystal X-ray diffraction analysis of both products unambiguously confirmed their molecular structures (for 4h[OTf]_2_ see the SI, Fig. S49; for 6h[OTf]_3_ see Fig. 4). The formation of 6h[OTf]_3_ underpins the previously observed capability of 3[OTf]_4_ to simultaneously act as a source of [L_C_–P]^+^ and [(L_C_–P)_2_]^2+^.^21,40^

### Dimerisation of imidazoliumyl-phosphaalkenes

While phosphaalkene 2h[OTf] can be isolated as a monomer from solutions of CH_3_CN, colorless precipitates are obtained in solvents of lower polarity, *i.e.* THF or C_6_H_5_F, after 16 hours at room temperature.

**Scheme 3:**
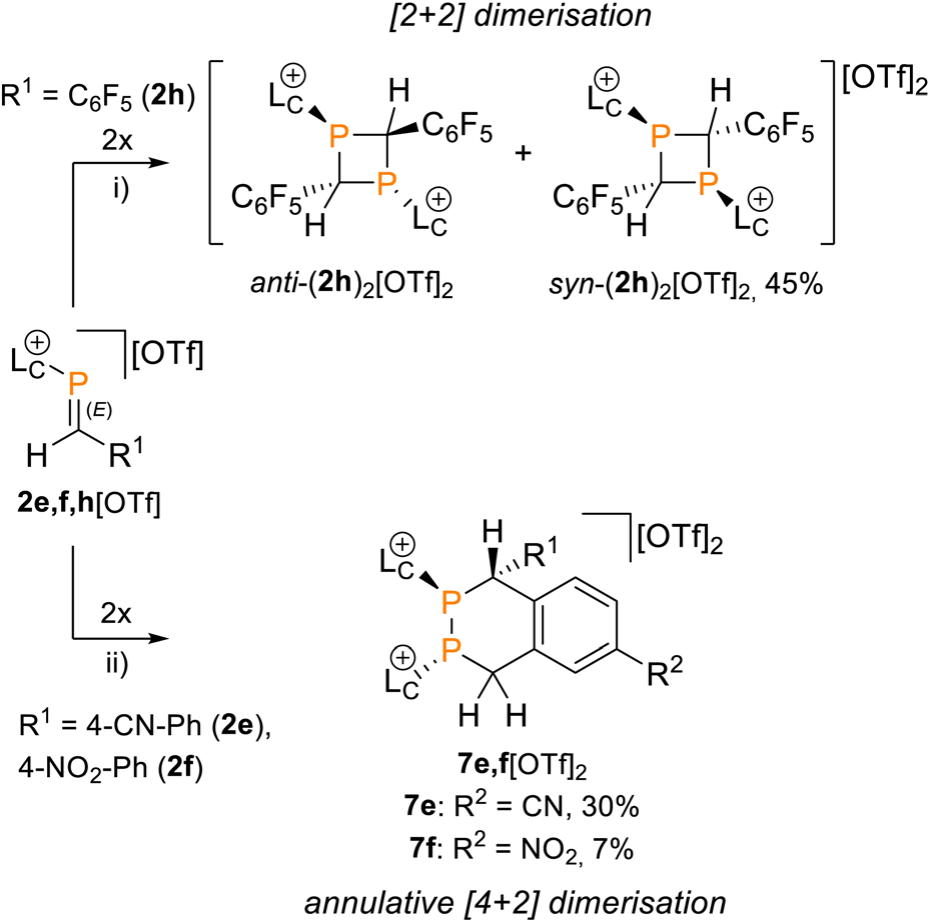
Formation of *syn*/*anti*-(2h)_2_[OTf]_2_*via* [2 + 2] dimerisation of 2h[OTf] in THF or C_6_H_5_F and [4 + 2] dimerisation of 2e,f[OTf] upon work-up to give 7e,f[OTf]_2_; reagents and conditions: (i) THF or C_6_H_5_F, r.t., 16 h, diastereomeric mixture (45% isolated yield for *syn*-(2h)_2_[OTf]_2_ after fractional recrystallisation); (ii) for 7e[OTf]_2_: THF work-up and fractional recrystallisation, r.t., 30%, for 7f[OTf]_2_: work-up over a silica plug and fractional recrystallisation, 7%.

**Fig. 5:**
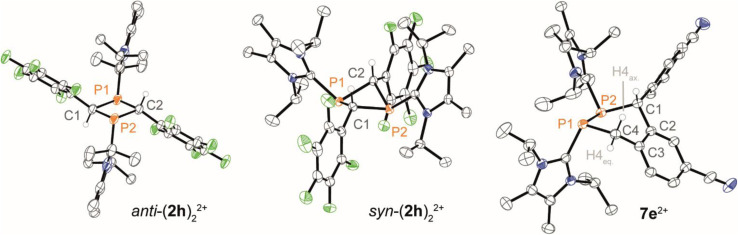
Molecular structure of *syn*/*anti*-(2h)_2_^2+^ in *syn*/*anti*-(2h)_2_[OTf]_2_ and 7e^2+^ in 7e[OTf]_2_·CH_3_CN; hydrogen atoms and anions are omitted for clarity, and thermal ellipsoids are displayed at 50% probability (100 K); selected bond lengths (Å) and angles (°): for *anti*-(2h)_2_^2+^: P1–C1 1.8783(19), P1–C2 1.9027(19), C1–P1–C2 86.30(8), P1–C1–P2 93.70(8), P1⋯P2 2.7586(11); for *syn*-(2h)_2_^2+^: P1–C1 avg. 1.891, P1–C2 avg. 1.894, C1–P1–C2 avg. 84.19, P1–C1–P2 avg. 92.82, P1⋯P2 avg. 2.5724; for 7e^2+^: P1–P2 2.2073(6), P1–C4 1.8636(16), P2–C1 1.8966(17), P1–C4–C3 118.22(12), P2–C1–C2 105.00(11), C1–P1–P2–C4 –25.17(8).

The ^31^P NMR spectrum of this solid in CH_3_CN shows two broadened resonances with low frequencies at *δ*(^31^P) = 19.0 ppm {*anti*-(2h)_2_[OTf]_2_} and *δ*(^31^P) = −10.9 ppm {*syn*-(2h)_2_[OTf]_2_} in a 50 : 50 ratio, indicating formation of dimers of 2h^+^ and loss of P=C double bond character (Scheme 3). Recrystallisation of the solid afforded two sets of colorless crystals suitable for X-ray diffraction analysis, confirming the formation of a *syn*- and *anti*-diastereomer of 1,3-diphosphetane *syn*/*anti*-(2h)_2_[OTf]_2_, respectively (Fig. 5). The *syn*/*anti* nomenclature refers to the relative position of the transannular imidazoliumyl substituents at the P_2_C_2_ core. Selected structural parameters are given in Fig. 5. Most importantly, the central P_2_C_2_ core in *anti*-(2h)_2_[OTf]_2_ adopts a planar geometry [Σ°(P_2_C_2_) = 360°, C1–P1–C1 86.30(8)°, P1–C1–P2 93.70(8)°], while the P_2_C_2_ core in *syn*-(2h)_2_[OTf]_2_ arranges in a butterfly motif with significantly reduced bond angles [C1–P1–C2 avg. 82.07°, P1–C1–P2 avg. 85.95°].

In line with metric parameters found in other structurally characterised 1,3-diphosphetanes, both diastereomers feature long P–C bond lengths [*anti*-(2h)_2_[OTf]_2_: P1–C1 1.8783(19) Å, P1–C2 1.9027(19) Å; *syn*-(2h)_2_[OTf]_2_: P1–C1 avg. 1.891 Å, P1–C2 avg. 1.894 Å, *cf.* P–C 1.855 Å^41^] reflecting strained ring systems.^31,33,38^ Importantly, this dimerisation is driven by the polarity of the solvent, reflecting another dimension of control for the dimerisation of cationic phosphaalkenes, next to steric and electronic stabilisation of the P=C double bond. Although representatives of 1,3-diphosphetanes have been structurally verified in both configurations,^10,11,19,42^ to the best of our knowledge (2h)_2_[OTf]_2_ is the first 1,3-diphosphetane for which both diastereomers are characterised by single crystal X-ray diffraction. Isolated *syn*-(2h)_2_[OTf]_2_ was obtained upon fractional recrystallisation and only mixed fractions were obtained for *anti*-(2h)_2_[OTf]_2_. No interconversions between the diastereomers or to the monomer were observed in a temperature range from 240–340 K using multinuclear NMR spectroscopy (see the SI, Fig. S61).

**Fig. 6:**
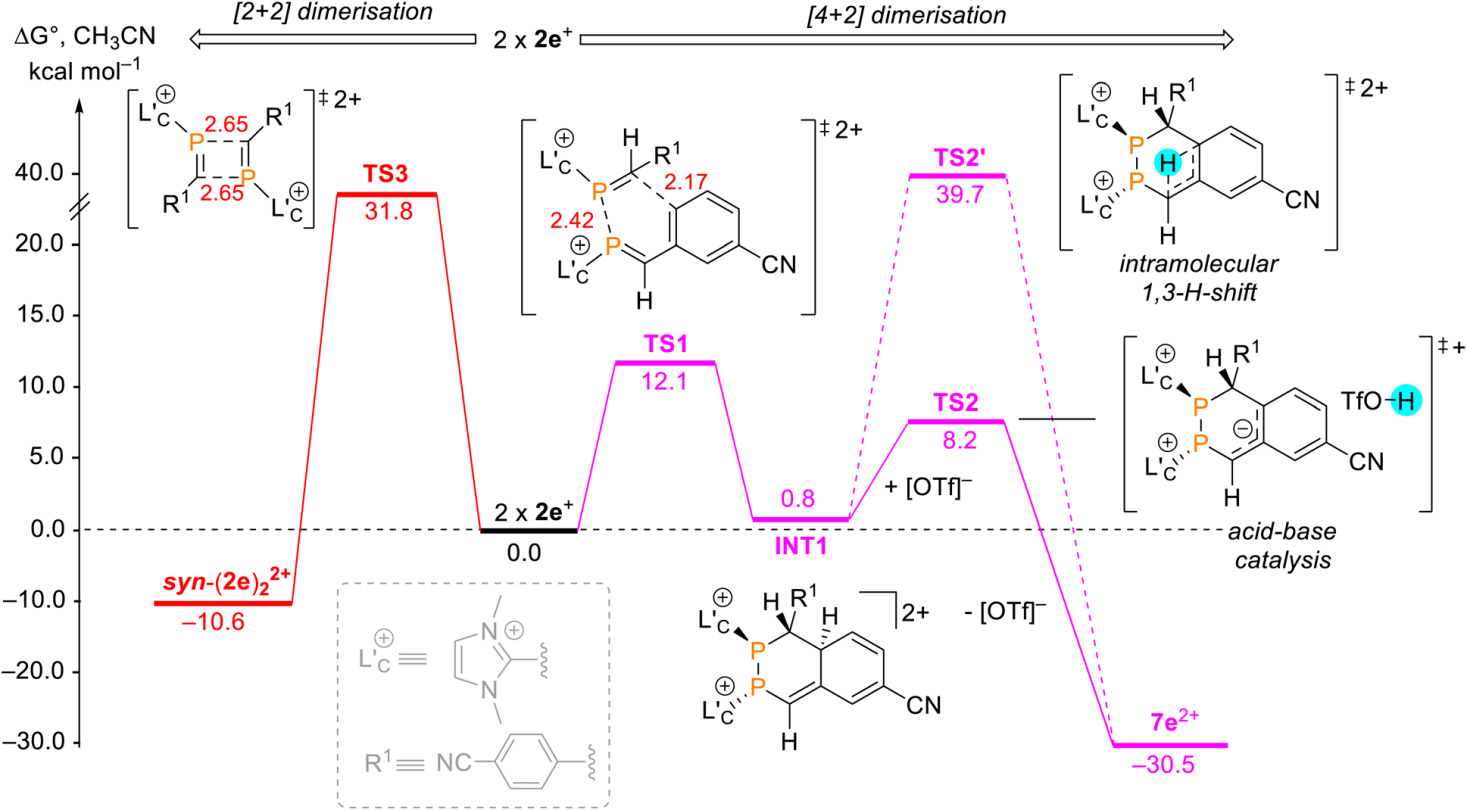
Calculated free energy profile (Δ*G*°, RI-BP86-D4/def2-TZVP, COSMO = CH_3_CN) for the transformation of two equiv. of 2e^+^ to 7e^2+^*via* a [4 + 2] dimerisation followed by acid-base catalysis (magenta pathway). For comparison, the competing [2 + 2] dimerisation to form *syn*-(2e)_2_^2+^ (red pathway) is also shown. All energies are reported in kcal mol^−1^ and are relative to two isolated molecules of 2e^+^. A truncated model for the imidazoliumyl substituent was used for all calculations.

Attempts to isolate the phosphaalkenes 2e,f^+^ from their reaction mixtures led to the serendipitous formation of annulated tetrahydro-1,2-diphosphinines 7e,f[OTf]_2_ (Scheme 3), which were obtained as crude solids in up to 85% (7e[OTf]_2_, 80–90% purity) and 58% yield (7f[OTf]_2_, 95% purity), respectively. The main impurities of the crude compounds were identified as the respective 1,3-diphosphetanes *syn*/*anti*-(2e,f)_2_[OTf]_2_ and the formation of *syn*-(2e)_2_[OTf]_2_ could be verified crystallographically (see the SI, Fig. S65). Analytically pure samples of 7e,f[OTf]_2_ were obtained by recrystallisation of the crude products, allowing the confirmation of their molecular structures by single crystal X-ray diffraction analysis (Fig. 5 and S84 in the SI). In the solid state the central six-membered ring of 7e^2+^ adopts a distorted boat conformation with *anti*-oriented imidazoliumyl substituents. Bond lengths and angles are as expected. Notably, the P–P bond distance [2.2073(6) Å] is consistent with a P–P single bond (Σ*r*_cov._ = 2.22 Å),^24^ the only other structurally characterised annulated tetrahydro-1,2-diphosphinine [P–P 2.2072(7) Å],^43^ and monocyclic 1,2,3,6-tetrahydrodiphosphinines.^44,45^ The diastereoselective formation of the stereogenic centers at P1, P2 and C1 in 7e,f[OTf]_2_ is supported by single sets of ^31^P NMR resonances that were iteratively fitted to AB spin systems [7e^2+^ (CD_2_Cl_2_, 300 K): exp., *δ*(^31^P_AB_): centered at −51.1 ppm; iteratively fitted, *δ*(^31^P_A_) = −52.2 ppm, *δ*(^31^P_B_) = −50.4 ppm, ^1^*J*(PP) = −190 Hz; 7f^2+^ (CD_3_CN, 300 K): exp., *δ*(^31^P_AB_): centered at −53.2 ppm; *δ*(^31^P_A_) = −55.8 ppm, *δ*(^31^P_B_) = −52.2 ppm, ^1^*J*(PP) = −193 Hz]. Additionally, the diastereotopic H4 protons are anisochronous at 300 K [7e^2+^: *δ*(^1^H4_ax_) = 4.52 ppm, *δ*(^1^H4_eq_) = 3.70 ppm, ^2^*J*(HH) = 15.1 Hz, 7f^2+^: *δ*(^1^H4_ax_) = 4.16 ppm, *δ*(^1^H4_eq_) = 3.84 ppm, ^2^*J*(HH) = 15.6 Hz], consistent with the absence of detectable conformational changes in solution (for details see the SI, Section S2.17 and S2.18). The diastereoselective formation of the six-membered ring in 7e,f^2+^ suggests a [4 + 2] dimerisation *via* a Phospha-Diels–Alder type reaction to be the operative pathway. Although auto-Phospha-Diels–Alder processes have been reported for 1-phosphabutadienes^12–14^ and 2*H*-phospholes^46^ they are elusive to *C*-aryl substituted phosphaalkenes as interactions of P=C double bonds with arenes are exceedingly rare.^47^ To rationalize this unusual reactivity we performed quantum chemical calculations (at the RI-BP86-D4/def2-TZVP level of theory) on the formation of 7e^2+^ from two molecules of 2e^+^, using a truncated imidazoliumyl substituent for computational efficiency (Fig. 6).

The reaction initiates with a concerted annulative [4 + 2] dimerisation of two molecules of 2e^+^, proceeding through transition state TS1 with a low activation barrier of 12.1 kcal mol^−1^. The resulting adduct INT1 is nearly isoenergetic with the reactants (Δ*G*° = +0.8 kcal mol^−1^), consistent with a reversible first step. Frontier orbital analysis (see the SI, Fig. S96) supports a normal-electron-demand Phospha-Diels–Alder process, where the HOMO is delocalized over the P=C and C=C bonds (diene) and the LUMO localizes on the P=C unit (dienophile).^48,49^

From INT1, two mechanistic scenarios were evaluated. An intramolecular 1,3 H shift *via* transition state TS2′ is kinetically inaccessible, with a barrier of 38.9 kcal mol^−1^. In contrast, an acid-base pathway mediated by the triflate counterion proved highly favourable. The acidic aryl C–H bond in INT1, destabilised by the electron-withdrawing cyano substituents and overall dicationic framework, is readily deprotonated by [OTf]^−^ (or another weak base in the reaction mixture), giving transition state TS2 (Δ*G*°^‡^ = 8.2 kcal mol^−1^). Subsequent reprotonation at the benzylic position occurs barrierless, directly yielding 7e^2+^, which is strongly stabilised at −30.5 kcal mol^−1^ relative to the starting materials. For comparison, the competing head-to-tail [2 + 2] dimerisation was calculated to be less favourable, with transition state TS3 at 31.8 kcal mol^−1^ for the formation of the corresponding 1,3-diphosphetane *syn*-(2e)_2_^2+^. Although the product is thermodynamically stable (Δ*G*° = −10.6 kcal mol^−1^), the high kinetic barriers render its formation pathway less viable. Notably, the calculations predict the formation of 1,2-diphosphetane to be more feasible than the 1,3-isomer (see the SI, Fig. S97). This discrepancy, along with the generally high absolute values of some calculated barriers (>30 kcal mol^−1^), is attributed to steric and electronic effects not fully captured by the truncated imidazoliumyl substituent used in the computational model. The preference for the annulative pathway is consistent with dominant formation of 7e,f[OTf]_2_ under the mild workup conditions used for 2e,f[OTf].

### Trapping reactions with 1,3-dienes

The Phospha-Diels–Alder reaction is an important tool to demonstrate the existence of thermodynamically unstable phosphaalkenes.^5,6,17,49,50^

**Scheme 4:**
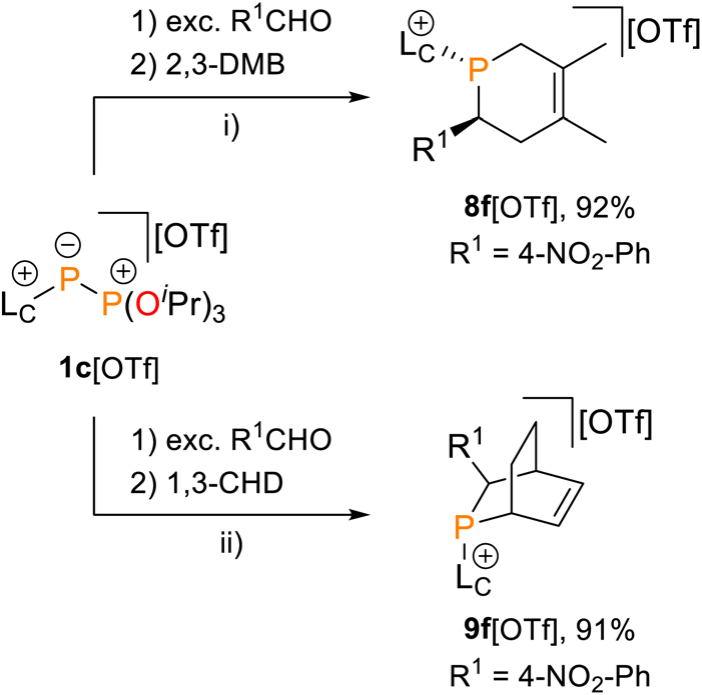
Phospha-Diels–Alder type trapping reactions of *in situ* prepared imidazoliumyl-substituted phosphaalkene 2f[OTf] with 2,3-dimethylbuta-1,3-diene (2,3-DMB) and 1,3-cyclohexadiene (1,3-CHD); reagents and conditions: (i) – OP(O^*i*^Pr)_3_, CH_2_Cl_2_, r.t., 16 h, 92%; (ii) – OP(O^*i*^Pr)3, CH_2_Cl_2_, r.t., 16 h, 91%.

**Fig. 7:**
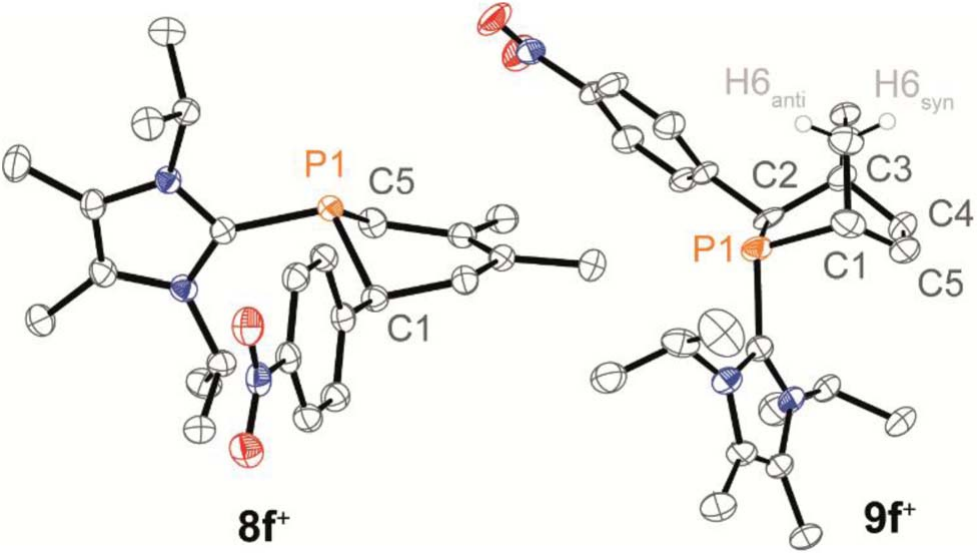
Molecular structures of 8f^+^ and 9f^+^ in 8f[OTf] and 9f[OTf]; selected hydrogen atoms and anions are omitted for clarity, and thermal ellipsoids are displayed at 50% probability (100 K); selected bond lengths (Å) and angles (°): for 8f^+^: P1–C1 1.854(2), P1–C5 1.8341(2), C1–P1–C5 96.9(1); for 9f^+^: P1–C2 1.869(2), P1–C1 1.882(4), C2–C3 1.575(5), P1–C1–C5 111.4(3), P1–C3–C4 109.9(3).

In this context, phosphaalkene 2f^+^ was trapped in reactions with dienes, namely 2,3-dimethylbuta-1,3-diene (2,3-DMB) and 1,3-cyclohexadiene (1,3-CHD), affording the *anti*-1,2,5,6-tetrahydrophosphinine 8f^+^ and *endo*-phosphabicyclo[2.2.2]oct-5-ene 9f^+^, respectively (Scheme 4). Both products were isolated as their triflate salts in excellent yields (92% and 91%), and their molecular structures were confirmed by single-crystal X-ray diffraction analysis (Fig. 7). The solid-state structures show the expected *anti* arrangement of the L_C_- and 4-NO_2_-phenyl substituent in line with the dominant *E*-configuration of 2f^+^. Bond lengths and angles fall within the expected ranges [*e.g.*, 8f^+^: P1–C1 1.854(2) Å, 9f^+^: P1–C2 1.869(2) Å, *cf.* P–C 1.855 Å],^41^ and compare well with structurally related 1,2,3,6-tetrahydrodiphosphinines and diphosphabicyclo[2.2.1]hept-5-enes.^45^ Compound 9f^+^ was identified as the *endo*-diastereomer, and multinuclear NMR spectroscopic investigation supports a diastereoselective formation of both products (see the SI for details, Section S2.20). For instance, the ^31^P NMR spectra of both heterocycles display only a single resonance at 300 K [8f^+^: *δ*(^31^P) = −47.6 ppm; 9f^+^: *δ*(^31^P) = −29.9 ppm].

## Conclusions

To conclude, we present a scalable synthesis of (imidazoliumyl)phosphito-phosphanides 1c,d[OTf] *via* nucleophilic fragmentation of tetraphosphetane 3[OTf]_4_. These reagents facilitate the cationic Phospha-Wittig reaction with a broad range of aldehydes for the first time, providing previously inaccessible *P*-imidazoliumyl-substituted and C–H functionalised phosphaalkenes 2[OTf]. These phosphaalkenes exhibit rich and chemoselective cycloaddition reactivity. They undergo: (I) [2 + 1] cyclopropenation reaction with cationic phosphinidene [L_C_–P]^+^ transfer reagents to afford the diphosphiranes 4[OTf] as well as rare triphosphetane 6h[OTf]_3_; (II) solvent-polarity-controlled [2 + 2] head-to-tail dimerisation to yield 1,3-diphosphetanes (2)_2_[OTf]_2_; (III) Phospha-Diels–Alder reactions with 1,3-dienes to tetrahydrophosphinine 8f[OTf] and phosphabicyclo[2.2.2]oct-5-ene 9f[OTf]; and (IV) a hitherto unknown annulative [4 + 2] dimerisation pathway of 2e,f[OTf], furnishing benzannulated tetrahydro-1,2-diphosphinines 7e,f[OTf]_2_. Mechanistic studies reveal that this transformation proceeds through an auto-Phospha-Diels–Alder step followed by a counter anion-guided proton-transfer and re-aromatisation sequence.

Collectively, these findings significantly expand the reactivity landscape of *C*-aryl-substituted phosphaalkenes and demonstrate that the cationic charge in *P*-imidazoliumyl phosphaalkenes unlocks new synthetic pathways inaccessible to their neutral analogues, a concept that could provide a blueprint for other transformations in main group chemistry. The new cationic heterocycles presented here are promising platforms for post-functionalisation, and further studies in this direction are underway and will be reported separately.

## Author contributions

P. R., K. S., and J. J. W. conceptualized the study; P. R. conducted the experiments and optimized the syntheses, isolations, and purifications; R. G. and A. F. were responsible for mechanistic studies; P. R. and J. J. W. were responsible for X-ray data collection and refinement; K. S. and J. J. W. conceived, oversaw, and directed the project; P. R. prepared the initial draft of the paper; A. F. and J. J. W. secured funding. All authors contributed to data analysis, manuscript review and editing, and discussion.

## Conflicts of interest

There are no conflicts to declare.

## Supplementary Material

SC-OLF-D5SC08693K-s001

SC-OLF-D5SC08693K-s002

## Data Availability

Additional data can be obtained from the corresponding author upon reasonable request. CCDC 2501246–2501257, 2517773 and 2517774 contain the supplementary crystallographic data for this paper.^51*a*–*n*^ Supplementary information (SI): the data supporting the findings of this study, including CIF files, NMR spectra, and computational details. See DOI: https://doi.org/10.1039/d5sc08693k.
