# Supplementary material for: Chemoselectivity in the cationic Phospha-Wittig reaction: accessing phosphorus heterocycles, phosphaalkenes, and their annulated [4 + 2] dimers
Source: Chem Sci. 2025 Dec 26;17(9):4678–87. doi: 10.1039/d5sc08693k (PMC12797139; doi:10.1039/d5sc08693k)
Supplement: SC-017-D5SC08693K-s001 [file SC-017-D5SC08693K-s001.pdf]

Supplementary Information (SI) for Chemical Science.
This journal is © The Royal Society of Chemistry 2025

Supporting Information

Chemoselectivity in the Cationic Phospha-Wittig reaction: Accessing Phosphorus-Heterocycles, Phosphaalkenes, and their annulated [4+2] Dimers

Philipp Royla,[a] Kai Schwedtmann,[a] Rosa M. Gomila,[b] Antonio Frontera,[b] and Jan J. Weigand*,[a]

[a] *Chair of Inorganic Molecular Chemistry, Faculty of Chemistry and Food Chemistry, Technische Universität Dresden, 01069 Dresden, Germany.*

[b] *Department of Chemistry, Universitat de Illes Balears, 07122 Palma de Mallorca, Spain.*

Content

S1. General remarks. 2

S2. Synthetic procedures, spectroscopic data and additional remarks. 3

S2.1 Reaction of 3[OTf]$_4$ with Phosphite Esters P(OR)$_3$ (R = Me, Et) 3

S2.2 Reaction of isolated 2h,i[OTf] with 0.25 equivalents of 3[OTf]$_4$ and catalytic amounts of Ph$_3$P 3

S2.3 Reaction of *in situ*-generated 2h[OTf] with 0.5 equivalents of 3[OTf]$_4$ and catalytic amounts of Ph$_3$P 4

S2.4 Preparation of [(L$_C$)P–P(O^{i}Pr)$_3$][OTf] (1c[OTf]) 5

S2.5 Preparation of [(L$_C$)P–P(OCy)$_3$][OTf] (1d[OTf]) 6

S2.6 General procedure for the reaction of 1c,d[OTf] with aldehydes 8

S2.7 Reaction of 1c[OTf] with 4-Methoxybenzaldehyde and Mesitylaldehyde 9

S2.8 Preparation of 1,2-di(imidazoliumyl)-3-phenyldiphosphirane 4a[OTf]$_2$ 9

S2.9 Preparation of 1,2-di(imidazoliumyl)-3-(4-bromophenyl)diphosphirane 4b[OTf]$_2$ 11

S2.10 Preparation of methyl (1,2-di(imidazoliumyl)diphosphiran-3-yl)benzoate 4c[OTf]$_2$ 13

S2.11 Preparation of [L$_C$P(OEt)C(H)(4-COOMePh)PH(L$_C$)][OTf]$_2$ 5c[OTf]$_2$ 15

S2.12 Preparation of [*(E)*-(L$_C$)P=C(H)C$_6$F$_5$][OTf] (2h[OTf]) 17

S2.13 Preparation of [*(E)*-(L$_C$)P=C(H)^{t}Bu][OTf] (2i[OTf]) 19

S2.14 Preparation of 1,2-di(imidazoliumyl)-3-pentafluorophenyldiphosphirane 4h[OTf]$_2$ 20

S2.15 Preparation of 1,2,3-tri(imidazoliumyl)-4-pentafluorophenyltriphosphetane 6h[OTf]$_3$ 24

S2.16 Preparation of [*syn*/*anti*-((L$_C$)P=C(H)C$_6$F$_5$)$_2$][OTf] (*syn*/*anti*-(2h)$_2$[OTf]$_2$) 27

S2.17 Preparation of 2,3-Di(imidazoliumyl)-7-cyano-1-(4-cyanophenyl)-1,2,3,4-tetrahydrobenzo[d][1,2]diphosphinine (7e[OTf]$_2$) 31

S2.18 Preparation of 2,3-Di(imidazoliumyl)-7-nitro-1-(4-nitrophenyl)-1,2,3,4-tetrahydrobenzo[d][1,2]diphosphinine (7f[OTf]$_2$) 36

S2.19 Preparation of *anti*-1-Imidazoliumyl-4,5-dimethyl-2-(4-nitrophenyl)-1,2,3,6-tetrahydrophosphinine (8f[OTf]) 40

S2.20 Preparation of *endo*-2-Imidazoliumyl-3-(4-nitrophenyl)-2-phosphabicyclo[2.2.2]oct-5-ene (9f[OTf]) 42

S3 Single Crystal X-ray Diffraction Data 45

S3.1 General remarks ... 45
S3.2 Refinement details ... 47
S4 Computational Details ... 52
S5 References ... 54
References ... 54
S6 Cartesian Coordinates ... 54

S1. General remarks.

All manipulations were performed in a Glovebox or using Schlenk techniques under an atmosphere of purified argon or nitrogen. Dry, oxygen-free solvents were distilled either from CaH_2 or from potassium. Deuterated solvents were purchased from Merck, Deutero or Eurisotop. Anhydrous deuterated acetonitrile (CD_3CN), dichloromethane (CD_2Cl_2) were purchased from Sigma-Aldrich. All solvents were stored over molecular sieves (4 Å: CH_2Cl_2, C_6H_5F, *o*-$C_6H_5F_2$, *n*-pentane, *n*-hexane, THF, THF-d_8, Et_2O, CD_2Cl_2, C_6D_6; 3 Å: CD_3CN, CH_3CN). All glassware was oven-dried at 160 °C prior to use. **3**[OTf]$_4$[1] and P(OCy)$_3$[2] were prepared according to literature reported procedures. 4-Bromobenzaldehyde, methyl 4-formylbenzoate, 3-cyanobenzaldehyde, 4-cyanobenzaldehyde, 3,5-bis(trifluoromethyl)benzaldehyde and pivalaldehyde were obtained from BLD Pharm and used as received. 4-Nitrobenzaldehyde was obtained from Fluka Chemie AG and used as received. Mesitylaldehyde was obtained from Acros Organics and used as received. 2,3,4,5,6-Pentafluorbenzaldehyd and 1,3-cyclohexadien were obtained from Sigma Aldrich and used as received. 2,3-Dimehtylbutadien was obtained from Thermo Fisher Scientific and used as received. Benzaldehyde, 4-methoxyaldehyde, P(OMe)$_3$, P(OEt)$_3$ and P(O^{i}Pr)$_3$ were obtained from Sigma Aldrich and distilled in an inert atmosphere prior to use. NMR spectra were measured on a Bruker AVANCE III HD Nanobay 400 MHz UltraShield (^{1}H: 400.13 MHz, ^{13}C: 100.61 MHz, ^{31}P: 161.98 MHz, ^{19}F: 376.50 MHz), or on a Bruker AVANCE III HDX, 500 MHz Ascend (^{1}H: 500.13 MHz, ^{13}C: 125.75 MHz, ^{31}P: 202.45 MHz, ^{19}F: 470.59 MHz). Reported numbers assigning atoms in the ^{13}C spectra were indirectly deduced from the cross-peaks in 2D correlation experiments (HMBC, HSQC). Chemical shifts are referenced to $\delta(Me_4Si)$ = 0.00 ppm (^{1}H, ^{13}C, externally), $\delta(CFCl_3)$ = 0.00 ppm (externally) and $\delta(H_3PO_4$, 85%) = 0.00 ppm (externally). Unless stated otherwise, all NMR spectra were measured at 300 K. Chemical shifts (δ) are reported in ppm. Coupling constants (J) are reported in Hz. The designation of the spin systems is performed by convention.[3] The furthest downfield resonance is denoted by the latest letter in the alphabet and the furthest upfield by the earliest letter. Melting points were recorded on an electrothermal melting point apparatus (Büchi Switzerland, Melting point M-560) in sealed capillaries under Nitrogen atmosphere and are uncorrected. Infrared (IR) and Raman spectra were recorded at ambient temperature using a Bruker Vertex 70 instrument equipped with a RAM II module (Nd: YAG laser, 1064 nm). The Raman intensities are reported in percent relative to the most intense peak and are given in parenthesis. An ATR unit (diamond) was used for recording IR spectra. The intensities are reported relative to the most intense peak and are given in parenthesis using the following abbreviations: vw = very weak, w = weak, m = medium, s = strong, vs = very strong. Elemental analyses were performed on a Vario MICRO cube Elemental Analyzer by Elementar Analysatorsysteme GmbH in CHNS modus.

S2. Synthetic procedures, spectroscopic data and additional remarks.

S2.1 Reaction of 3[OTf]$_4$ with Phosphite Esters P(OR)$_3$ (R = Me, Et)

To a solution of **3**[OTf]$_4$ (1.0 equiv.) in CH_3CN (4 ml, R = Me) or CD_2Cl_2 (4 ml, R = Et), a solution of P(OR)$_3$ (4.2 equiv.) CH_3CN (1 ml, R = Me) or CD_2Cl_2 (1 ml, R = Et) was added. The resulting yellow reaction mixture was stirred at room temperature for 16 hours until an aliquot was removed and analyzed by means of multinuclear NMR spectroscopy. The ^{31}P NMR spectrum of the reaction mixture (**Figure S1**) evidenced the formation of **1a**,**b**$^+$. Removal of all volatiles *in vacuo* and stirring over Et_2O (4 ml) gave off-white oily residues. Multinuclear NMR spectroscopic analysis of the oily residue (R = Me) showed a resonance at $\delta(^{31}P)$ = 55.0 ppm consistent with the presence of **3**$^{4+}$ in the solution. Analysis of single crystals obtained from vapor diffusion of Et_2O into a CD_3CN solution of the oily residue at −30°C (R = Me) confirmed the formation of **3**[OTf]$_4$.

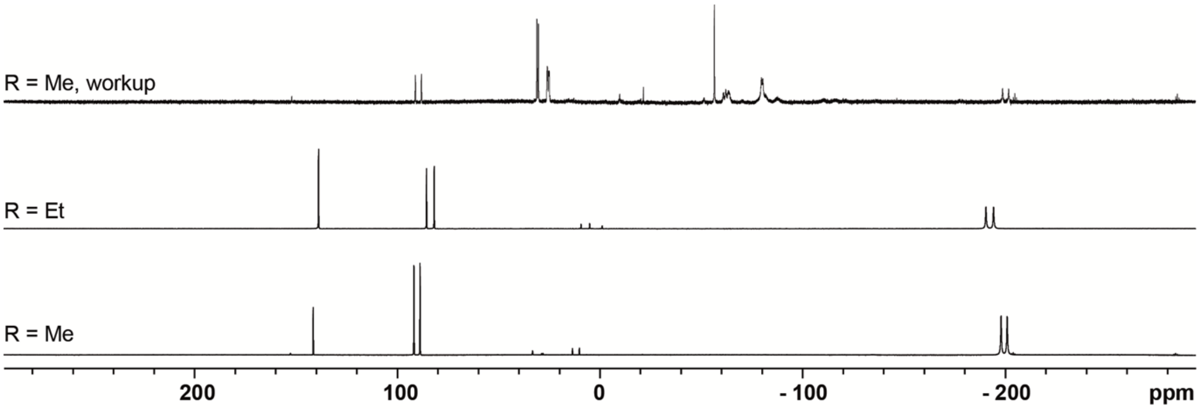

Figure S1. ^{31}P NMR spectra of the reaction mixtures of **3**[OTf]$_4$ with R(OR)$_3$ and ^{31}P NMR spectrum obtained from the oily residue during the work-up of the reaction with P(OMe)$_3$ (C_6D_6 cap. or CD_3CN, 300 K).

The X part of the AX spin systems of **1a-d**[OTf] (see **Table S1**) was observed at substantially higher frequencies compared to the analogous resonances in phosphanylidene-phosphoranes[4] or phosphonio-phosphanides[1]. This pronounced shift can be attributed to the electron-withdrawing nature of the alkoxy substituents, which reduce the electron density at the two-coordinated phosphorus center. The trend parallels observations made in phosphinite-stabilized systems.[5] Consistent with this, relatively small ^{1}J(PP) coupling constants were observed similar to trends known for the ^{1}J(PP) coupling constants in diphosphanes.[6] Moreover, stronger donor effects of the alkyl substitutens R in the phosphite group correlated with shielding of the X part and at the same time deshielding of the A part in **1a-d**$^+$. This reflects the competition between R-group electron donation and negative hyperconjugation into the σ*(P−O) antibonds.

Table S1. Parameters of the AX spin system of **1a-d**$^+$ in their $^{31}P\{^1H\}$ NMR spectra (300K, C_6D_6 cap., CH_3CN) and related compounds.

	$\delta(^{31}P_A)$ in ppm	$\delta(^{31}P_X)$ in ppm	^{1}J(PP) in Hz
**1a**[OTf]	−199.4	90.2	−605
**1b**[OTf]	−192.4	83.6	−608
**1c**[OTf]	−182.4	78.3	−601
**1d**[OTf]	−182.5	79.3	−608
[L_CP–PMe$_3$][OTf][1]	−167.0	12.0	−472
[L_CP–PEt$_3$][OTf][1]	−202.0	36.0	−492
[L_CP–PCy$_3$][OTf][1]	−208.8	38.1	−545
[L_CP–PPh$_3$][OTf][1]	−168.6	31.3	−519
DmpP–PPh$_3$[4]	−138.8	25.2	−639
DmpP–PMe$_3$[4]	−114.7	−2.8	−582

S2.2 Reaction of isolated 2h,i[OTf] with 0.25 equivalents of 3[OTf]$_4$ and catalytic amounts of Ph$_3$P

A solution of **2h**[OTf] (50.0 mg, 0.09 mmol, 1.0 eq.) in CH_3CN (2 ml) was added to a solution of **3**[OTf]$_4$, (33.4 mg, 0.02 mmol, 0.25 eq.) and Ph$_3$P (2.5 mg, <0.01 mmol, 0.1 eq.) in CH_3CN (2 ml). The resulting pale-yellow reaction mixture

was stirred for four days at room temperature and subsequently analyzed by means of ^{31}P NMR spectroscopic analysis (**Figure S2**).

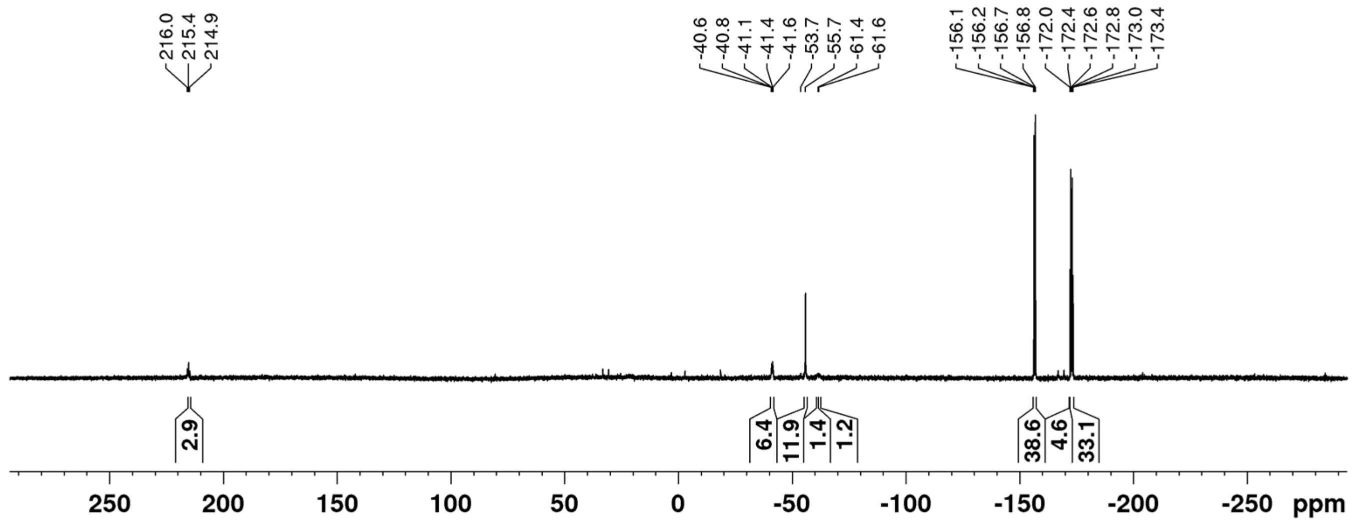

Figure S2. ^{31}P NMR spectrum of the reaction mixture of **2h**[OTf], 0.25 equivalents **3**[OTf]$_4$, and catalytic amounts of Ph_3P after stirring for four days at room temperature (C_6D_6 cap., CH_3CN, 300 K).

A solution of **2i**[OTf] (50.0 mg, 0.16 mmol, 1.0 eq.) in CH_3CN (2 ml) was added to a solution of **3**[OTf]$_4$, (41.9 mg, 0.03 mmol, 0.25 eq.) and Ph_3P (3.0 mg, <0.01 mmol, 0.1 eq.) in CH_3CN (2 ml). The resulting pale-yellow reaction mixture was stirred for four days at room temperature and analyzed by means of ^{31}P NMR spectroscopic analysis (**Figure S2**). Subsequently, the mixture was heated to 70°C for two hours and again subjected to ^{31}P NMR spectroscopic analysis indicating no meaningful formation of diphosphiranes **4i**$^{2+}$.

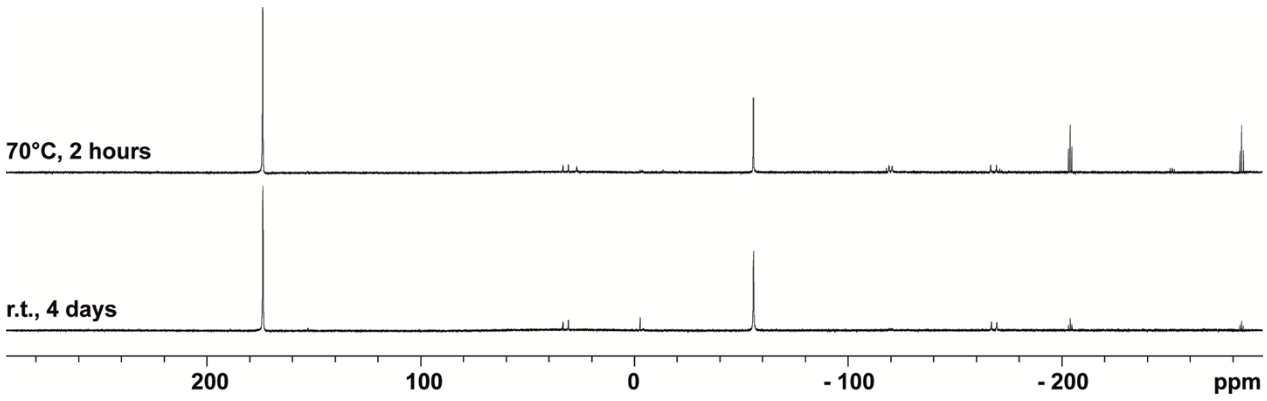

Figure S3. ^{31}P NMR spectrum of the reaction mixture of **2i**[OTf], 0.25 equivalents **3**[OTf]$_4$, and catalytic amounts of Ph_3P after stirring for four days at room temperature and afte heating to 70°C for two hours (C_6D_6 cap., CH_3CN, 300 K).

S2.3 Reaction of *in situ*-generated 2h[OTf] with 0.5 equivalents of 3[OTf]$_4$ and catalytic amounts of Ph_3P

To a solid mixture of **1c**[OTf] (250 mg, 0.44 mmol, 1.0 equiv.), **3**[OTf]$_4$ (380 mg, 0.26 mmol, 0.6 equiv.), Ph_3P (16 mg, 0.04 mmol, 0.15 equiv.) and pentafluorophenylaldehyde (172 mg, 0.88 mmol, 2.0 equiv.) CH_3CN (10 ml) was added. The resulting pale-yellow solution was stirred for three days at room temperature and analyzed by multinuclear NMR analysis (**Figure S4**). Subsequently, the reaction mixture was heated to 50°C for 16 h followed by heating to 70°C for 16 h and analyzed by means of ^{31}P NMR spectroscopic analysis after each heating step indicating no meaningful conversion (**Figure S4**).

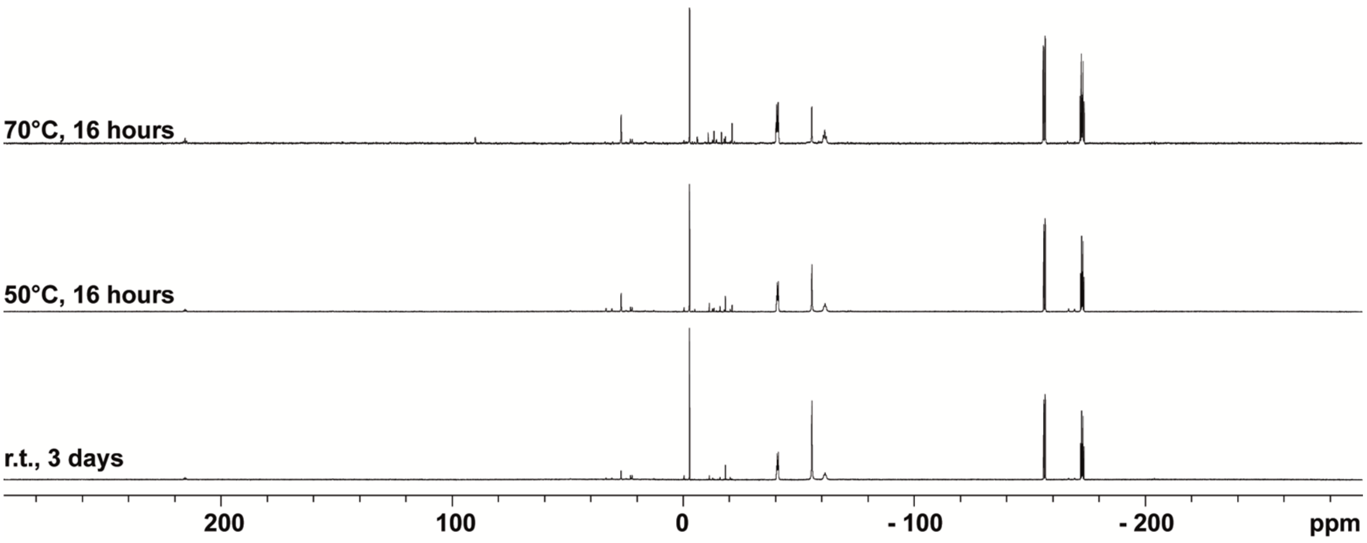

Figure S4. ^{31}P NMR spectrum of the reaction mixture of *in situ*-generated **2h**[OTf], 0.5 equivalents **3**[OTf]$_4$, and catalytic amounts of Ph_3P after stirring at varying temperatures (C_6D_6 cap., CH_3CN, 300 K).

S2.4 Preparation of [(L$_C$)P–P(O^{i}Pr)$_3$][OTf] (1c[OTf])

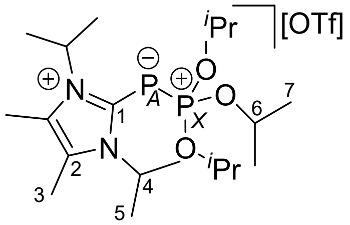

To a solution of **3**[OTf]$_4$ (2.50 g, 1.30 mmol, 1.0 equiv.) in CH_3CN (15 ml), P(O*i*Pr)$_3$ (1.56 ml, 1.46 g, 7.0 mmol, 4.05 equiv.) was added dropwise while stirring. The resulting pale-yellow solution was stirred for 16 hours at room temperature and evaporated to dryness. Subsequent washing of the residue with Et_2O (3x10 ml) and consecutive drying *in vacuo* afforded the product as an analytically pure air- and moisture-sensitive off-white solid.

Yield: 3.58 g (91%); **m.p.**: 92-94 °C (decomp.); **Raman** (100 mW, 500 scans, 298 K, in cm^{-1}): 2985 (62), 2943 (100), 2925 (77), 2878 (31), 2765 (9), 2737 (12), 1620 (34), 1450 (54), 1414 (39), 1390 (21), 1357 (57), 1333 (22), 1294 (85), 1222 (19), 1190 (16), 1143 (28), 1103 (21), 1088 (17), 1031 (60), 1006 (15), 972 (14), 954 (12), 937 (12), 896 (24), 889 (31), 790 (21), 771 (20), 751 (33), 722 (28), 704 (18), 655 (13), 591 (50), 571 (24), 551 (20), 519 (14), 499 (16), 461 (24), 452 (23), 435 (45); **IR** (ATR, 298 K, , in cm^{-1}): 2983 (vw), 2938 (vw), 2879 (vw), 1619 (vw), 1467 (vw), 1454 (vw), 1389 (w), 1377 (w), 1347 (vw), 1265 (vs), 1220 (w), 1177 (vw), 1143 (s), 1101 (w), 1030 (m), 988 (vs), 971 (vs), 908 (vw), 896 (w), 887 (w), 767 (m), 721 (vw), 636 (vs), 588 (m), 570 (w), 551 (vw), 516 (w), 499 (w), 466 (vw), 450 (w), 437 (w); **^{1}H NMR** (400.13 MHz, CD_3CN, 300 K, in ppm): δ = 1.30 (18H, d, ^3J(HH) = 6.2 Hz, H7), 1.51 (12H, d, ^3J(HH) = 7.2 Hz, H5), 2.34 (6H, d, ^5J(HP) = 1.4 Hz, H3), 4.80 (3H, dsept, ^3J(HP) = 6.5 Hz, ^3J(HH) = 6.3 Hz, H6), 5.91 (2H, m, H4); **^{13}C{^{1}H} NMR** (100.61 MHz, CD_3CN, 300 K, in ppm): δ = 10.9 (2C, s, C3), 21.2 (4C, s, C5), 23.9 (6C, d, ^3J(CP) = 3 Hz, C7), 54.4 (2C, d, ^3J(CP) = 7 Hz, C4), 76.7 (3C, dd, ^2J(CP) = 9 Hz, ^3J(CP) = 2 Hz, C6), 122.2 (1C, q, ^1J(CF) = 321 Hz, OTf), 130.2 (2C, s, C2), 146.7 (1C, dd, ^1J(CP) = 85 Hz, ^2J(CP) = 8 Hz, C1); **^{19}F{^{1}H} NMR** (376.5 MHz, CD_3CN, 300 K, in ppm): δ = −79.2 (3F, s, OTf); **^{31}P{^{1}H} NMR** (161.98 MHz, CD_3CN, 300 K, in ppm): δ = −182.4 (1P, d, ^1J(PP) = −599 Hz, P$_A$), 78.3 (1P, d, ^1J(PP) = −599 Hz, P$_X$); **elemental analysis**: calcd. for $C_{21}H_{41}F_3N_2O_6P_2S$: C: 44.36, H: 7.27, N: 4.93, S: 5.64; found: C: 44.01, H: 7.250, N: 4.94, S: 5.556.

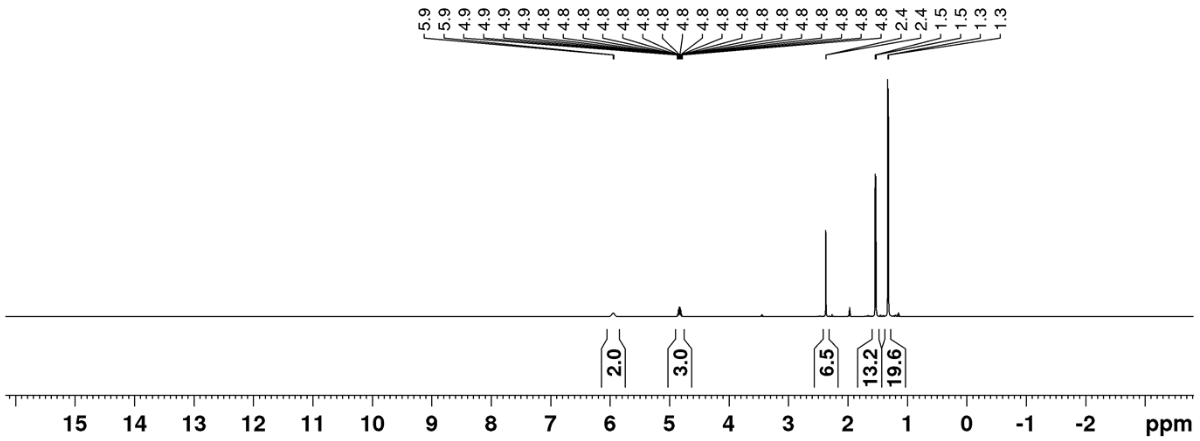

Figure S5. ^{1}H NMR spectrum of **1c**[OTf] (CD_3CN, 300 K).

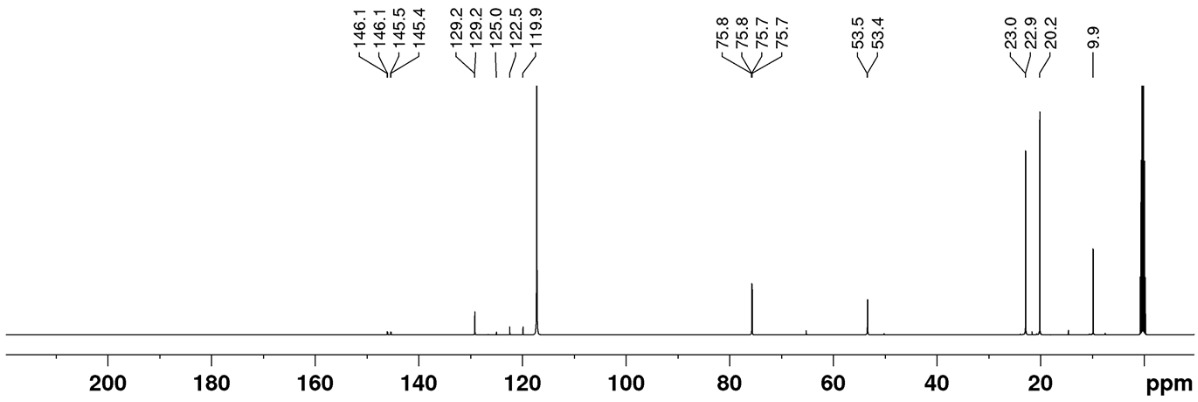

Figure S6. ^{13}C NMR spectrum of **1c**[OTf] (CD_3CN, 300 K).

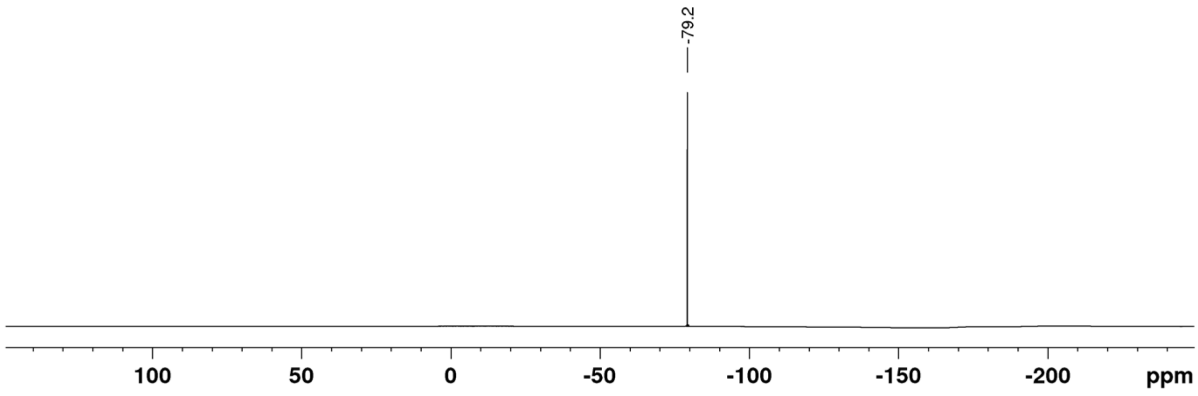

Figure S7. ^{19}F NMR spectrum of **1c**[OTf] (CD_3CN, 300 K).

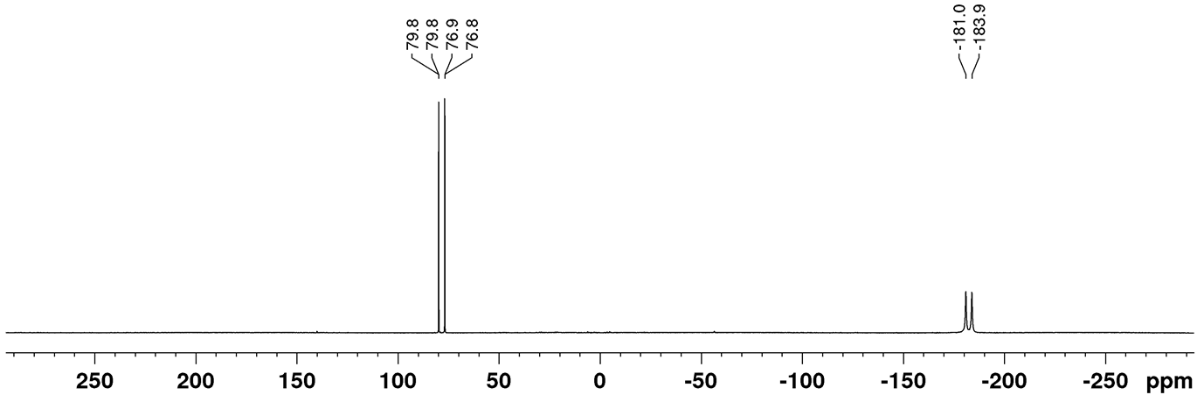

Figure S8. ^{31}P NMR spectrum of **1c**[OTf] (CD_3CN, 300 K).

S2.5 Preparation of [(L_C)P–P(OCy)$_3$][OTf] (1d[OTf])

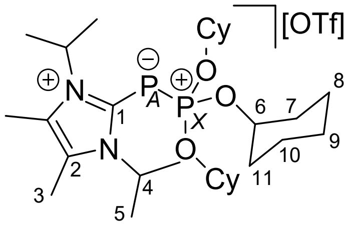

To a solution of **3**[OTf]$_4$ (1.50 g, 1.04 mmol, 1.0 equiv.) in CH_3CN (5 ml), a suspension of $P(OCy)_3$ (1.44 g, 4.37 mmol, 4.2 equiv.) in CH_3CN (5 ml) was added while stirring. The resulting dark-orange solution was stirred for 16 hours at room temperature, filtered, and reduced to a volume of 3 ml *in vacuo*. Subsequent addition of Et_2O (15 ml) led to the precipitation of an off-white solid, which was filtered and dried *in vacuo* to afford the product as an air- and moisture-sensitive off-white solid (595 mg). Storing the filtrate at –30°C for 16 h afforded analytically pure colorless crystals, which were separated, washed with Et_2O (2x2 ml) and dried *in vacuo* (1.75 g). Single crystals suitable for single crystal X-ray diffraction analysis were obtained via the same method. Full characterization was performed using the second crystalline batch of product.

Yield: 2.35 g (82%); **m.p.**: 241-243 °C (decomp.); **Raman** (400 mW, 50 scans, 298 K, in cm^{-1}): 2975 (27), 2946 (39), 1605 (24), 1454 (11), 1410 (23), 1355 (33), 1271 (100), 1149 (14), 1086 (6), 1033 (17), 884 (5), 792 (12), 755 (8),

696 (5), 573 (5), 510 (27), 442 (6), 374 (18), 348 (7), 312 (7), 126 (7); **IR** (ATR, 298 K, in cm^{-1}): 2982 (vw), 2935 (w), 2861 (vw), 1619 (vw), 1462 (vw), 1449 (vw), 1392 (w), 1372 (vw), 1267 (vs), 1220 (w), 1145 (m), 1114 (vw), 1031 (s), 1013 (w), 990 (m), 952 (m), 905 (vw), 886 (w), 870 (vw), 791 (vw), 774 (vw), 751 (vw), 650 (w), 636 (s), 596 (vw), 571 (vw), 562 (vw), 516 (vw), 501 (vw), 430 (vw), 416 (vw), 409 (vw); **^{1}H NMR** (500.13 MHz, CD_3CN, 300 K, in ppm): δ = 1.53 (12H, d, $^{3}J_{HH}$ = 7.2 Hz, H5), 1.70 (6H, m, H8$_{eq}$ and H10$_{eq}$), 1.89 (6H, m, H7$_{eq}$ and H11$_{eq}$), 2.37 (6H, d, $^{5}J_{HP}$ = 1.4 Hz, H3), 4.59 (3H, m, H6$_{ax}$), 5.94 (2H, m, H4), 1.25-1.47 (9H, m, H8$_{ax}$ and H9$_{ax}$ and H10$_{ax}$), 1.48-1.60 (9H, m, H7$_{ax}$ and H9$_{eq}$ and H11$_{ax}$); **^{13}C{^{1}H} NMR** (125.76 MHz, CD_3CN, 300 K, in ppm): δ = 9.9 (2C, s, C3), 20.3 (4C, s, C5), 23.1 (6C, s, C8 and C10), 24.6 (3C, s, C9), 33.0 (6C, d, $^{3}J_{CP}$ = 4 Hz, C7 and C11), 53.5 (2C, d, $^{3}J_{CP}$ = 8 Hz, C4), 80.0 (3C, dd, $^{2}J_{CP}$ = 9 Hz, $^{3}J_{CP}$ = 4 Hz, C6), 121.2 (1C, q, $^{1}J_{CF}$ = 321 Hz, OTf), 129.2 (2C, d, $^{3}J_{CP}$ = 1 Hz, C2), 146.0 (1C, dd, $^{1}J_{CP}$ = 86 Hz, $^{2}J_{CP}$ = 9 Hz, C1); **^{19}F{^{1}H} NMR** (470.59 MHz, CD_3CN, 300 K, in ppm): δ = -79.3 (3F, s, OTf); **^{31}P{^{1}H} NMR** (202.46 MHz, CD_3CN, 300 K, in ppm): δ = –182.5 (1P, d, $^{1}J_{PP}$ = -608 Hz, P$_{A}$), 79.3 (1P, d, $^{1}J_{PP}$ = -604 Hz, P$_{X}$).

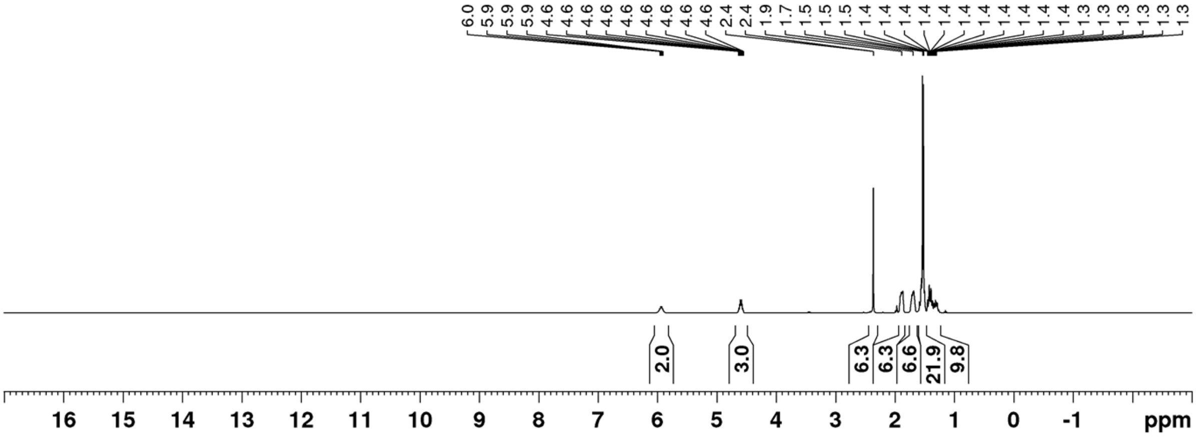

Figure S9. ^{1}H NMR spectrum of **1d**[OTf] (CD_3CN, 300 K).

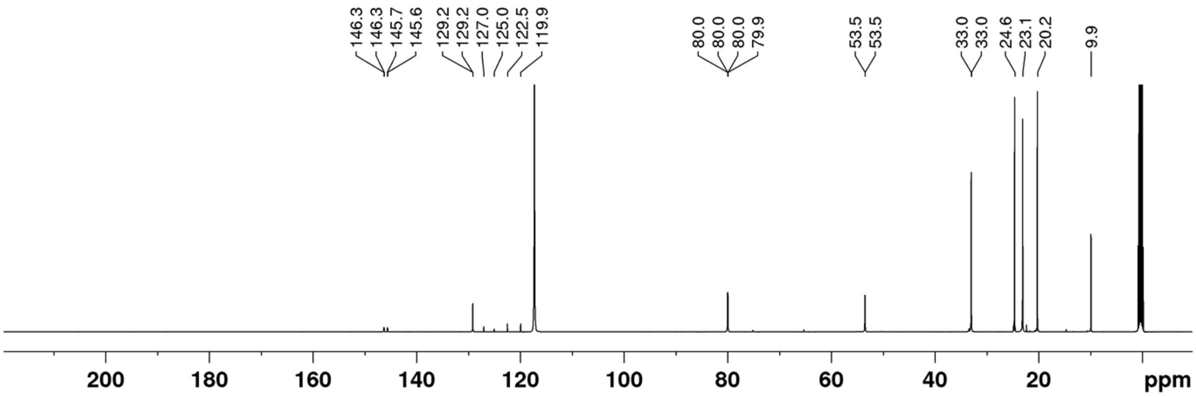

Figure S10. ^{13}C NMR spectrum of **1d**[OTf] (CD_3CN, 300 K).

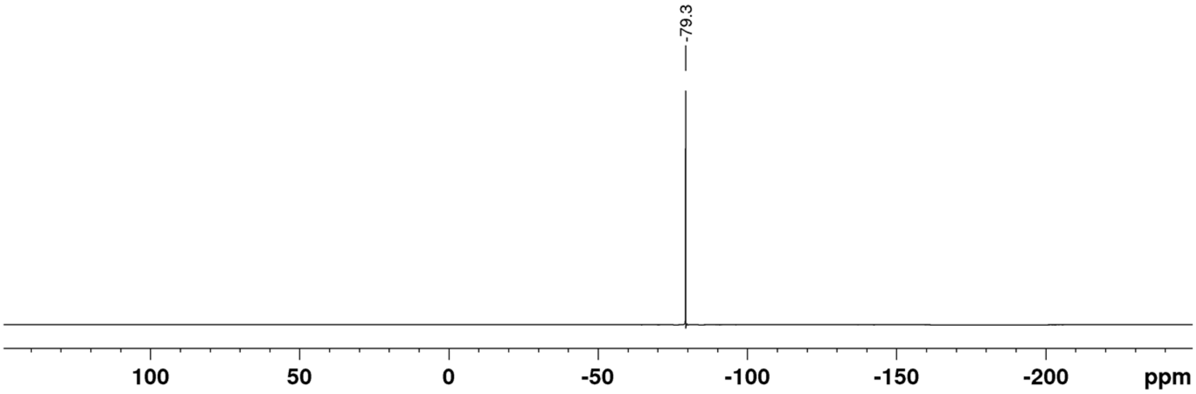

Figure S11. ^{19}F NMR spectrum of **1d**[OTf] (CD_3CN, 300 K).

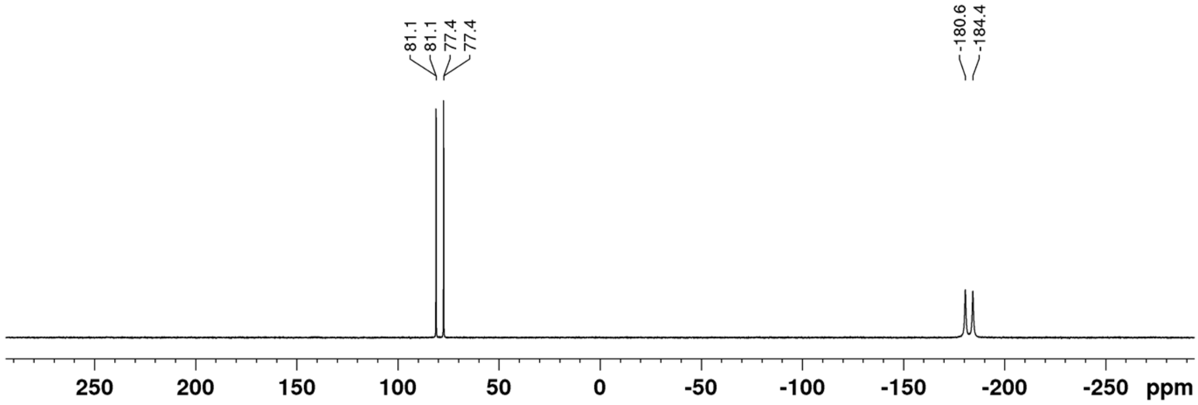

Figure S12. ^{31}P NMR spectrum of **1d**[OTf] (CD_3CN, 300 K).

S2.6 General procedure for the reaction of 1c,d[OTf] with aldehydes

To a solution of **1c**[OTf] (50 mg, 0.09 mmol, 1.0 equiv.) or **1d**[OTf] (50 mg, 0.07 mmol, 1.0 equiv.) in CH_3CN (2 ml) a solution of an aldehyde (5.0 equiv) in CH_3CN (1 ml) was added and the solution was allowed to stir at room temperature until complete conversion of **1c,d**[OTf] was observed by means of ^{31}P NMR spectroscopy (**Figure S13**, for details see **Table 1**).

A representative ^{31}P NMR spectrum for the conversion of **1c**[OTf] and 5 equiv. of PhCHO after 16 h and 4 d is shown in **Figure S14.**

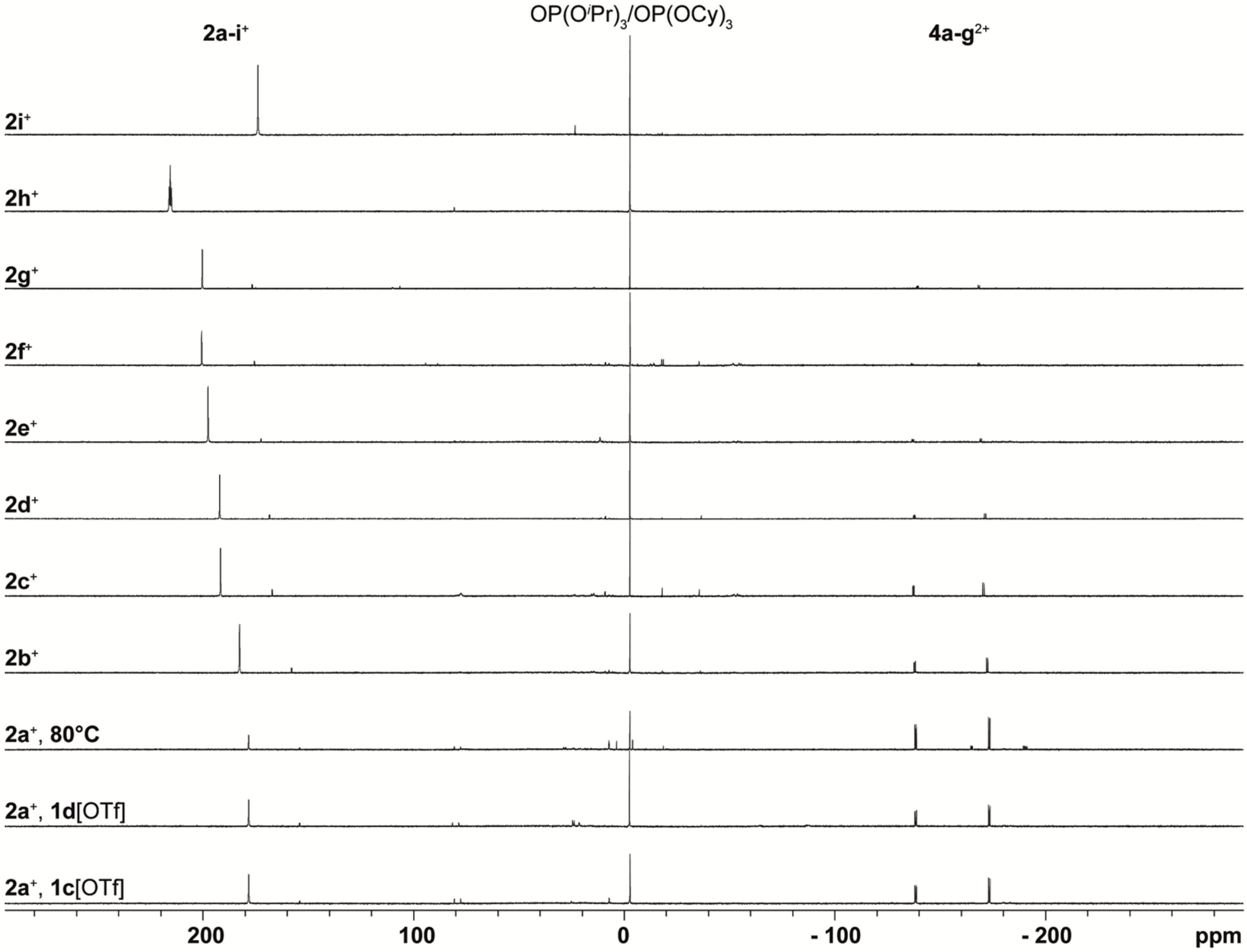

Figure S13. ^{31}P NMR spectra of the fully converted reaction mixtures of **1c,d**[OTf] with 2-10 equivalents of an aldehyde (C_6D_6 cap., 300 K).

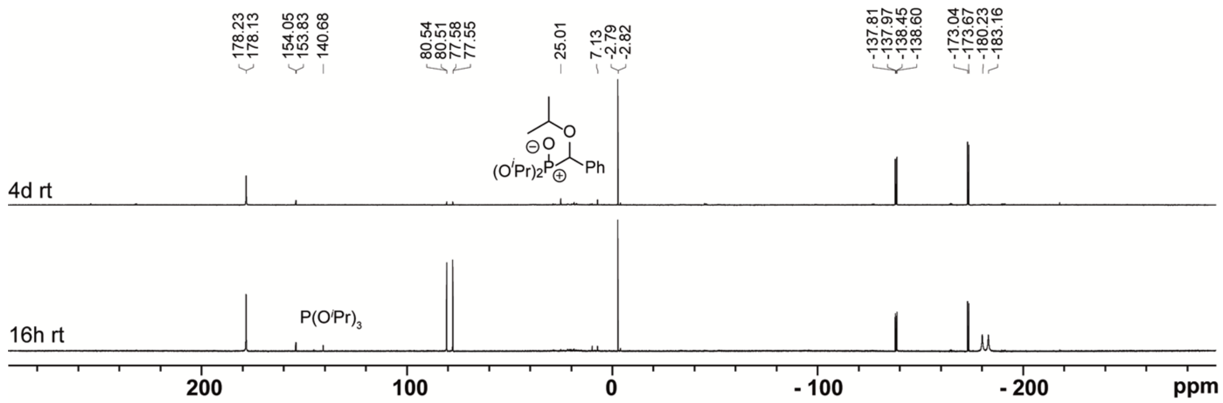

Figure S14. ^{31}P NMR spectra of the conversion progress in the reaction of **1c**[OTf] with a 5-fold excess of benzaldehyde in CH_3CN after 16 h and 4 d (in CH_3CN, C_6D_6 cap., 300 K).

S2.7 Reaction of 1c[OTf] with 4-Methoxybenzaldehyde and Mesitylaldehyde

To a solution of **1c**[OTf] (50.0 mg, 0.09 mmol, 1.0 equiv.) in CH_3CN, an excess of 4-methoxybenzaldehyde (54 µl, 60.0 mg, 0.44 mmol, 5.0 equiv.) or mesitylaldehyde (65 µl, 65.0 mg, 0.44 mmol, 5.0 equiv.) was added, and the resulting pale-yellow reaction mixtures were stirred at room temperature for two days. Subsequently, an aliquot removed from each solution was subjected to multinuclear NMR spectroscopic analysis (**Figure S15**). Next, 15 equivalents of 4-methoxybenzaldehyde or mesitylaldehyde, respectively, were added to the mixtures, and stirred for another three days at room temperature. Subsequently, aliquots of both mixtures were investigated by means of multinuclear NMR spectroscopic analysis (**Figure S15**).

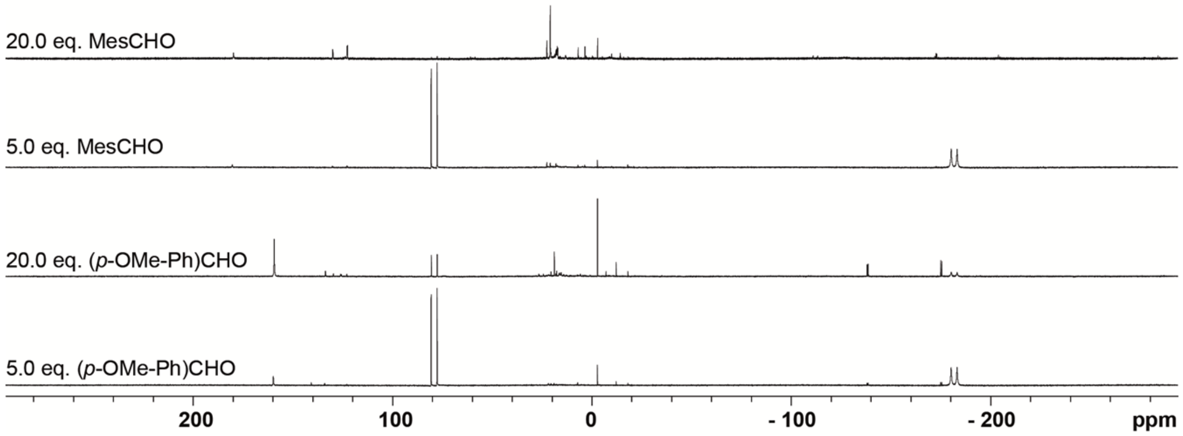

Figure S15. ^{31}P NMR spectra of the reaction solution of **1c**[OTf] with 4-methoxybenzaldehyde and mesitylaldehyde in CH_3CN at room temperature (C_6D_6 cap., 300 K).

S2.8 Preparation of 1,2-di(imidazoliumyl)-3-phenyldiphosphirane 4a[OTf]$_2$

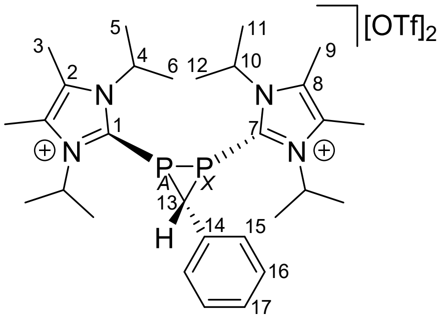

To a solid mixture of **1c**[OTf] (250 mg, 0.44 mmol, 1.0 equiv.), **3**[OTf]$_4$ (222 mg, 0.15 mmol, 0.32 equiv.), and Ph_3P (12 mg, 0.04 mmol, 0.1 equiv.) a solution of benzaldehyde (233 mg, 2.2 mmol, 5.0 equiv.) in CH_3CN (10 ml) was added. The resulting pale-yellow solution was stirred for seven days at room temperature and evaporated to dryness. Subsequently, the sticky residue was stirred over Et_2O (10 ml), the supernatant was removed, and the residue was dried *in vacuo* to afford a pale-yellow foam. The addition of Et_2O (15 ml) to a clear solution of this solid in C_6H_5F (4 ml) afforded a brownish oil, which was again dissolved in C_6H_5F (4 ml) and precipitated by the addition of *n*-hexane (10 ml) for two more times. Drying of the occurring oil *in vacuo* gave a foam, which was again dissolved in C_6H_5F (4 ml) and the solution was layered with *n*-hexane (10 ml) and stored at –30°C. An amorphous precipitate formed within 72 h, which was separated by decantation and stirred over n-hexane for 16 h, and subsequently dried *in vacuo* to afford the crude product as an off-white powder in ~85% purity, determined by ^{31}P NMR spectroscopy.

Note: Further attempts to separate **4a**[OTf]$_2$ from this mixture by washing with different solvent combinations, recrystallization or purification over a silica plug where either unsuccessful or led to the unproductive decomposition of **4a**[OTf]$_2$. The purity of the crude product was sufficient for the unambiguous assignment of all atoms in multinuclear NMR spectroscopy experiments.

Yield: 343 mg (96%, 85% purity); **m.p.**: 154-156°C (decomp.) ; **Raman** (400 mW, 100 scans, 298 K, in cm^{-1}): 3064 (27), 2986 (63), 2946 (100), 2882 (20), 2740 (7), 1619 (27), 1599 (30), 1450 (37), 1417 (40), 1365 (40), 1286 (60), 1223 (7), 1189 (13), 1154 (13), 1088 (7), 1032 (67), 1001 (47), 886 (17), 790 (13), 754 (27), 736 (13), 618 (10), 574 (23), 546 (7), 481 (20), 462 (20), 348 (20), 311 (23), 276 (13), 112 (10); **IR** (ATR, 298 K, in cm^{-1}): 2984 (vw), 2943 (vw), 1618 (vw), 1494 (vw), 1453 (vw), 1398 (vw), 1378 (vw), 1260 (vs), 1222 (w), 1146 (m), 1113 (vw), 1030 (s), 982 (vw), 938 (vw), 904 (vw), 885 (vw), 836 (vw), 787 (vw), 769 (vw), 753 (vw), 724 (vw), 699 (vw), 636 (vs), 594 (vw), 572 (w), 548 (vw), 517 (m), 466 (vw), 439 (vw); **^{1}H NMR** (400.13 MHz, CD_3CN, 300 K, in ppm): δ = 1.12 (6H, d(br), $^3J_{HH}$ = 6.8 Hz, H11), 1.61 (6H, d(br), $^3J_{HH}$ = 6.9 Hz, H12), 1.61 (6H, d, $^3J_{HH}$ = 6.9 Hz, H5), 1.70 (6H, d, $^3J_{HH}$ = 7.0 Hz, H6), 2.30 (6H, s, H3), 2.39 (6H, s, H9), 4.48 (1H, dd, $^2J_{HP}$ = 30.7 Hz, $^2J_{HP}$ = 3.6 Hz, H13), 5.15 (4H, m, H4 and H10), 6.90 (2H, m, H15), 7.36 (3H, m, H16 and H17); **^{13}C{^{1}H} NMR** (100.61 MHz, CD_3CN, 300 K, in ppm): δ = 10.1 (2C, s, C9), 10.3 (2C, s, C3), 19.5 (2C, s, C11), 20.2 (2C, s, C6), 20.3 (2C, d, $^4J_{CP}$ = 3 Hz, C5), 20.6 (2C, d, $^4J_{CP}$ = 2 Hz, C12), 42.4 (1C, dd, $^1J_{CP}$ = 58 Hz, $^1J_{CP}$ = 46 Hz, C13), 54.8 (2C, m, C4), 55.1 (2C, m, C10), 121.2 (1C, q, $^1J_{CF}$ = 321 Hz, OTf), 127.8 (2C, d, $^3J_{CP}$ = 10 Hz, C15), 128.7 (1C, s, C17), 129.7 (2C, s, C16), 131.5 (2C, s, C8), 132.2 (2C, s, C2), 132.8 (1C, dd, $^2J_{CP}$ = 16 Hz, $^2J_{CP}$ = 4 Hz, C14), 137.2 (1C, dd, $^1J_{CP}$ = 92 Hz, $^2J_{CP}$ = 13 Hz, C7), 139.0 (1C, dd, $^1J_{CP}$ = 85 Hz, $^2J_{CP}$ = 9 Hz, C1); **^{19}F NMR** (376.5 MHz, CD_3CN, 300 K, in ppm): δ = -79.2 (3F, s, OTf); **^{31}P NMR** (161.98 MHz, CD_3CN, 300 K, in ppm): δ = -174.1 (1P, d, $^1J_{PP}$ = 128 Hz, P_A), -138.9 (1P, dd, $^1J_{PP}$ = 128 Hz, $^2J_{PH}$ = 31 Hz, P_X).

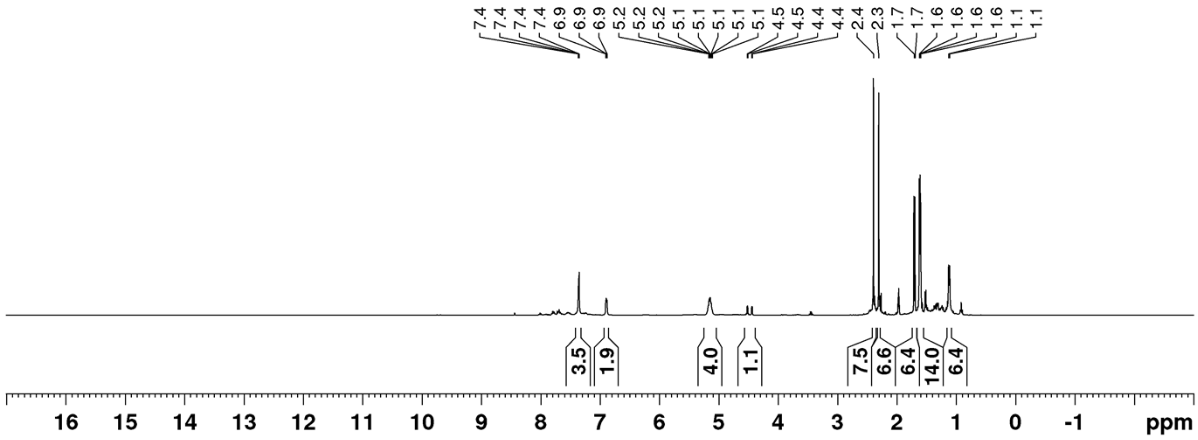

Figure S16. ^{1}H NMR spectrum of crude **4a**[OTf] (CD_3CN, 300 K).

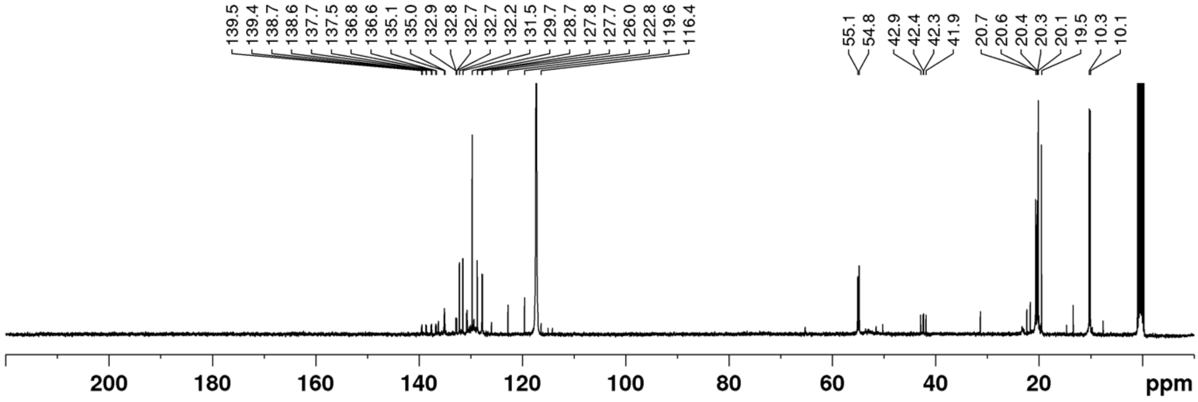

Figure S17. ^{13}C NMR spectrum of crude **4a**[OTf] (CD_3CN, 300 K).

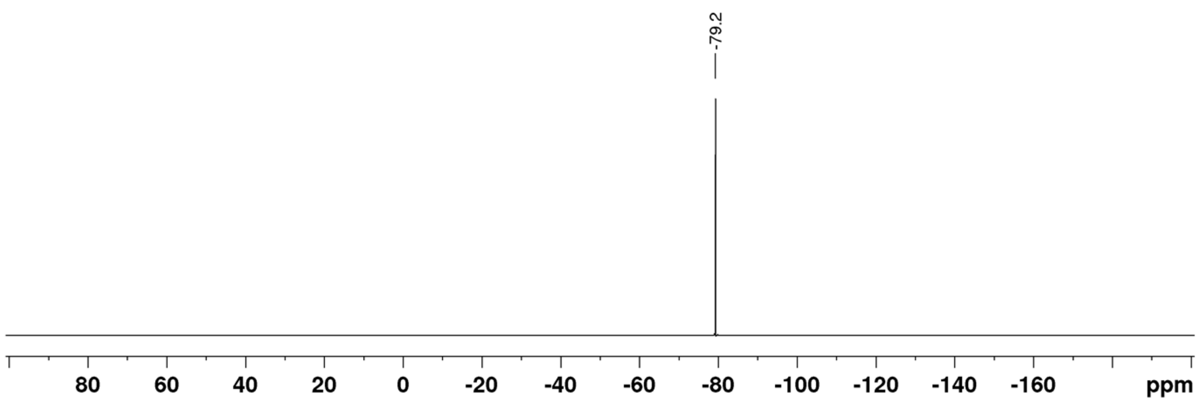

Figure S18. ^{19}F NMR spectrum of crude **4a**[OTf] (CD_3CN, 300 K).

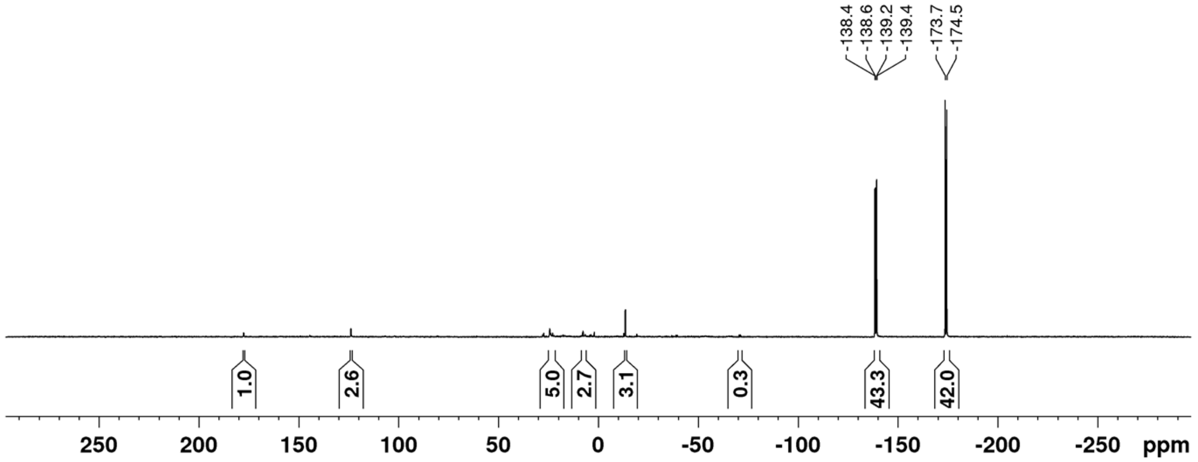

Figure S19. ^{31}P NMR spectrum of crude **4a**[OTf] (CD_3CN, 300 K).

S2.9 Preparation of 1,2-di(imidazoliumyl)-3-(4-bromophenyl)diphosphirane 4b[OTf]$_2$

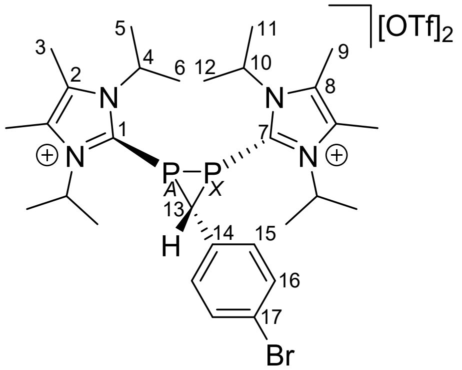

To a solid mixture of **1c**[OTf] (250 mg, 0.44 mmol, 1.0 equiv.), **3**[OTf]$_4$ (222 mg, 0.15 mmol, 0.32 equiv.), Ph_3P (12 mg, 0.04 mmol, 0.1 equiv.) and 4-bromobenzaldehyde (407 mg, 2.2 mmol, 5.0 equiv.) CH_3CN (10 ml) was added. The resulting pale-yellow solution was stirred for four days at room temperature and evaporated to dryness. Subsequent washing of the residue with Et_2O (10 ml) consecutive drying *in vacuo* afforded a pale-yellow solid, which was mainly contaminated with residual aldehyde. Subsequent washing steps with cold C_6H_5F (3x2 ml) afforded a colorless solid that was stirred over n-pentane (10 ml) and dried *in vacuo* to afford the product as an air- and moisture-sensitive off-white solid. Single crystals suitable for single crystal X-ray diffraction analysis were obtained by diffusion of n-hexane into a saturated *o*-$C_6H_4F_2$ solution of the product at room temperature.

Yield: 259 mg (66%); **m.p.**: 172-174°C (decomp.); **Raman** (400 mW, 50 scans, 298 K, in cm^{-1}): 3071 (14), 2977 (69), 2944 (100), 2887 (21), 1620 (34), 1590 (69), 1450 (41), 1419 (76), 1406 (45), 1366 (90), 1287 (97), 1258 (38), 1222 (10), 1183 (28), 1155 (21), 1081 (17), 1031 (69), 887 (21), 876 (17), 789 (21), 753 (31), 744 (21), 702 (7), 662 (7), 641 (7), 583 (24), 493 (24), 475 (17), 454 (31), 400 (7), 384 (7), 371 (7), 346 (24), 309 (21), 274 (14), 223 (7), 110 (17); **IR** (ATR, 298 K, in cm^{-1}): 2988 (vw), 2387 (vw), 1618 (vw), 1490 (vw), 1467 (vw), 1441 (vw), 1415 (vw), 1379 (vw), 1268 (s), 1221 (w), 1178 (vw), 1147 (m), 1117 (vw), 1082 (vw), 1030 (s), 1012 (vw), 1000 (vw), 907 (vw), 827 (vw), 789 (vw), 753 (vw), 716 (vw), 661 (vw), 636 (vs), 572 (w), 547 (vw), 517 (w), 493 (vw), 476 (vw), 458 (vw), 445 (vw); **^{1}H NMR** (500.13 MHz, CD_3CN, 300 K, in ppm): δ = 1.16 (6H, d(br), $^3J_{HH}$ = 6.9 Hz, H11), 1.60 (6H, d(br), $^3J_{HH}$ = 6.9 Hz, H12), 1.60 (6H, d, $^3J_{HH}$ = 7.0 Hz, H5), 1.69 (6H, d, $^3J_{HH}$ = 7.0 Hz, H6), 2.32 (6H, s, H3), 2.39 (6H, s, H9), 4.43 (1H, dd, $^2J_{HP}$ = 30.5 Hz, $^2J_{HP}$ = 3.3 Hz, H13), 5.12 (4H, m, H4 and H10), 6.81 (2H, m, H15), 7.53 (2H, m, H16); **^{13}C{^{1}H} NMR** (125.76 MHz, CD_3CN, 300 K, in ppm): δ = 10.1 (2C, s, C9), 10.3 (2C, s, C3), 19.6 (2C, s, C11), 20.1 (2C, s, C6), 20.3 (2C, d, $^4J_{CP}$ = 3 Hz, C5), 20.6 (2C, d, $^4J_{CP}$ = 2 Hz, C12), 41.7 (1C, dd, $^1J_{CP}$ = 58 Hz, $^1J_{CP}$ = 46 Hz, C13), 54.9 (2C, m, C4), 55.1 (2C, m,

C10), 121.2 (1C, q, $^{1}J_{CF}$ = 321 Hz, OTf), 122.0 (1C, d, $^{5}J_{CP}$ = 2 Hz, C17), 129.7 (2C, d, $^{3}J_{CP}$ = 10 Hz, C15), 131.6 (2C, s, C8), 132.3 (1C, dd, $^{2}J_{CP}$ = 17 Hz, $^{2}J_{CP}$ = 3 Hz, C14), 132.4 (2C, s, C2), 132.7 (2C, s, C16), 136.7 (1C, dd, $^{1}J_{CP}$ = 92 Hz, $^{2}J_{CP}$ = 14 Hz, C7), 138.7 (1C, dd, $^{1}J_{CP}$ = 85 Hz, $^{2}J_{CP}$ = 9 Hz, C1); **^{19}F NMR** (470.59 MHz, CD_3CN, 300 K, in ppm): δ = -79.3 (3F, s, OTf); **^{31}P NMR** (202.46 MHz, CD_3CN, 300 K, in ppm): δ = -173.1 (1P, d, $^{1}J_{PP}$ = 128 Hz, P_A), -138.7 (1P, dd, $^{1}J_{PP}$ = 128 Hz, $^{2}J_{PH}$ = 31 Hz, P_X); **elemental analysis**: calcd. for $C_{31}H_{45}BrF_6N_4O_6P_2S_2$: C: 41.85, H: 5.10, N: 6.30, S: 7.21; found: C: 41.78, H: 4.879, N: 6.25, S: 7.157.

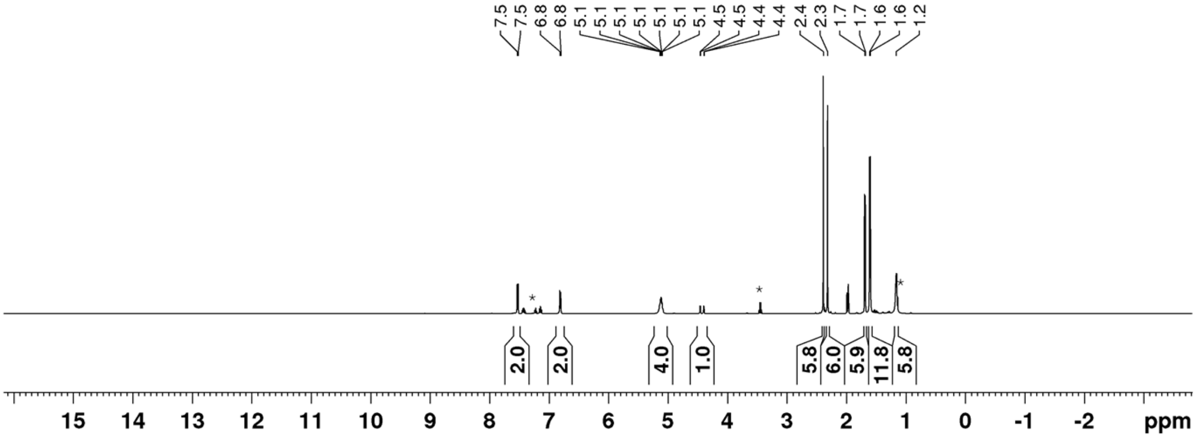

Figure S20. ^{1}H NMR spectrum of **4b**[OTf]$_2$ (CD_3CN, 300 K). Asterisks mark residual amounts of C_6H_5F and Et_2O.

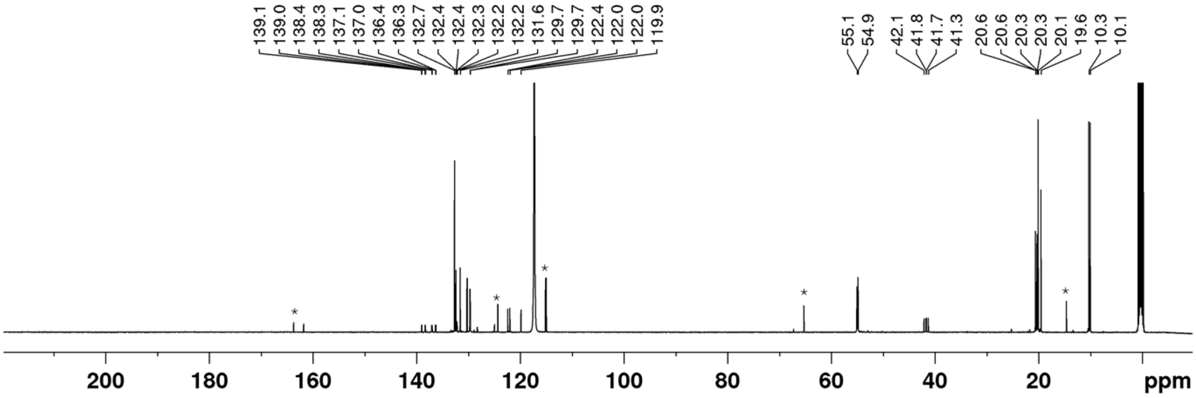

Figure S21. $^{13}C\{^{1}H\}$ NMR spectrum of **4b**[OTf]$_2$ (CD_3CN, 300 K). Asterisks mark residual amounts of C_6H_5F and Et_2O.

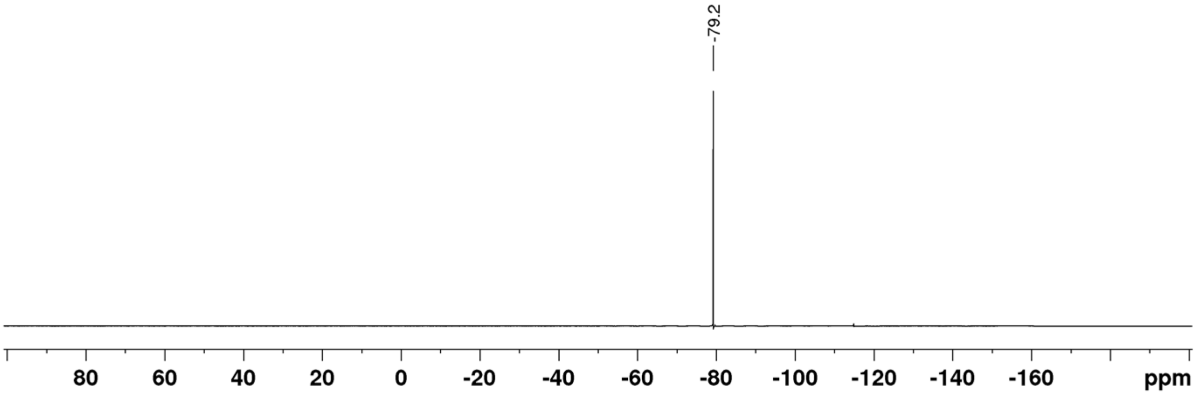

Figure S22. ^{19}F NMR spectrum of **4b**[OTf]$_2$ (CD_3CN, 300 K).

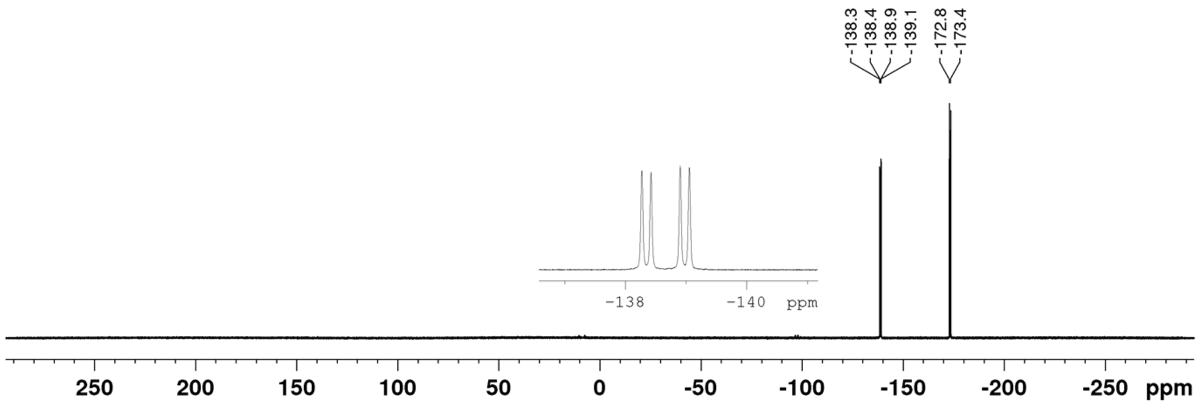

Figure S23. ^{31}P NMR spectrum of **4b**[OTf]$_2$ (CD_3CN, 300 K).

S2.10 Preparation of methyl (1,2-di(imidazoliumyl)diphosphiran-3-yl)benzoate 4c[OTf]$_2$

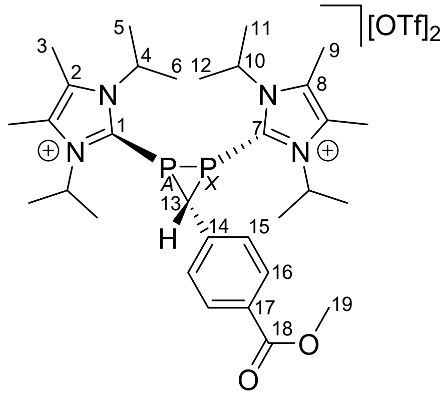

To a solid mixture of **1c**[OTf] (250 mg, 0.44 mmol, 1.0 equiv.), **3**[OTf]$_4$ (222 mg, 0.15 mmol, 0.32 equiv.), Ph_3P (12 mg, 0.04 mmol, 0.1 equiv.) and methyl 4-formylbenzoate (361 mg, 2.2 mmol, 5.0 equiv.) CH_3CN (10 ml) was added. The resulting pale-yellow solution was stirred for four days at room temperature and evaporated to dryness. Subsequently, the residue was stirred over Et_2O (10 ml) and toluene (5 ml) for 16 h before the supernatant was decanted and replaced by fresh Et_2O (15 ml) and stirred for another 16 h at room temperature. Subsequent filtration and washing steps with Et_2O (3x4 ml) afforded a colorless solid that was dried *in vacuo* to afford the product as a crude, air- and moisture-sensitive off-white solid.

Yield: 351 mg (80% purity, 92%); **m.p.**: 152-154°C ; **Raman** (400 mW, 50 scans, 298 K, in cm^{-1}): 3071 (21), 2986 (52), 2946 (87), 2880 (22), 1722 (24), 1608 (100), 1449 (30), 1416 (31), 1365 (30), 1285 (55), 1223 (9), 1185 (15), 1152 (12), 1116 (9), 1088 (6), 1032 (46), 1000 (9), 885 (13), 824 (18), 790 (9), 754 (19), 741 (9), 574 (18), 459 (10), 348 (16), 311 (16), 276 (9), 113 (7); **IR** (ATR, 298 K, in cm^{-1}): 2984 (vw), 2946 (vw), 2880 (vw), 1720 (w), 1607 (vw), 1439 (vw), 1412 (vw), 1398 (vw), 1377 (vw), 1257 (s), 1221 (w), 1184 (vw), 1146 (m), 1110 (w), 1029 (s), 979 (vw), 903 (vw), 861 (vw), 839 (vw), 823 (vw), 776 (vw), 753 (vw), 724 (vw), 704 (vw), 660 (vw), 636 (vs), 571 (w), 540 (vw), 516 (w), 447 (vw); **^{1}H NMR** (500.13 MHz, CD_3CN, 300 K, in ppm): δ = 1.12 (6H, s(br), H11), 1.61 (12H, d(br), $^{3}J_{HH}$ = 6.9 Hz, H5 and H12), 1.70 (6H, d, $^{3}J_{HH}$ = 7.0 Hz, H6), 2.30 (6H, s, H3), 2.39 (6H, s, H9), 3.89 (3H, s, H19), 4.53 (1H, dd, $^{2}J_{HP}$ = 30.5 Hz, $^{2}J_{HP}$ = 3.0 Hz, H13), 5.13 (4H, m, H4 and H10), 7.00 (2H, d, $^{3}J_{HH}$ = 8.1 Hz, H15), 7.96 (3H, d, $^{3}J_{HH}$ = 8.5 Hz, H16); **^{13}C{^{1}H} NMR** (125.76 MHz, CD_3CN, 300 K, in ppm): δ = 10.1 (2C, s, C9), 10.3 (2C, s, C3), 19.6 (2C, s, C11), 20.1 (2C, s, C6), 20.4 (2C, d, $^{4}J_{CP}$ = 3 Hz, C5), 20.6 (2C, d, $^{4}J_{CP}$ = 2 Hz, C12), 41.8 (1C, dd, $^{1}J_{CP}$ = 58 Hz, $^{1}J_{CP}$ = 46 Hz, C13), 52.0 (1C, s, C19), 54.9 (2C, m, C4), 55.1 (2C, m, C10), 121.2 (1C, q, $^{1}J_{CF}$ = 321 Hz, OTf), 128.1 (2C, d, $^{3}J_{CP}$ = 10 Hz, C15), 130.4 (1C, s, C17), 130.4 (2C, s, C16), 131.6 (2C, s, C8), 132.4 (2C, s, C2), 136.7 (1C, dd, $^{1}J_{CP}$ = 92 Hz, $^{2}J_{CP}$ = 13 Hz, C7), 138.3 (1C, dd, $^{2}J_{CP}$ = 16 Hz, $^{2}J_{CP}$ = 3 Hz, C14), 138.6 (1C, dd, $^{1}J_{CP}$ = 84 Hz, $^{2}J_{CP}$ = 9 Hz, C1), 165.9 (1C, s, C18); **^{19}F NMR** (470.59 MHz, CD_3CN, 300 K, in ppm): δ = -79.2 (3F, s, OTf); **^{31}P NMR** (202.46 MHz, CD_3CN, 300 K, in ppm): δ = -171.4 (1P, d, $^{1}J_{PP}$ = 131 Hz, P_A), -138.0 (1P, dd, $^{1}J_{PP}$ = 131 Hz, $^{2}J_{PH}$ = 31 Hz, P_X).

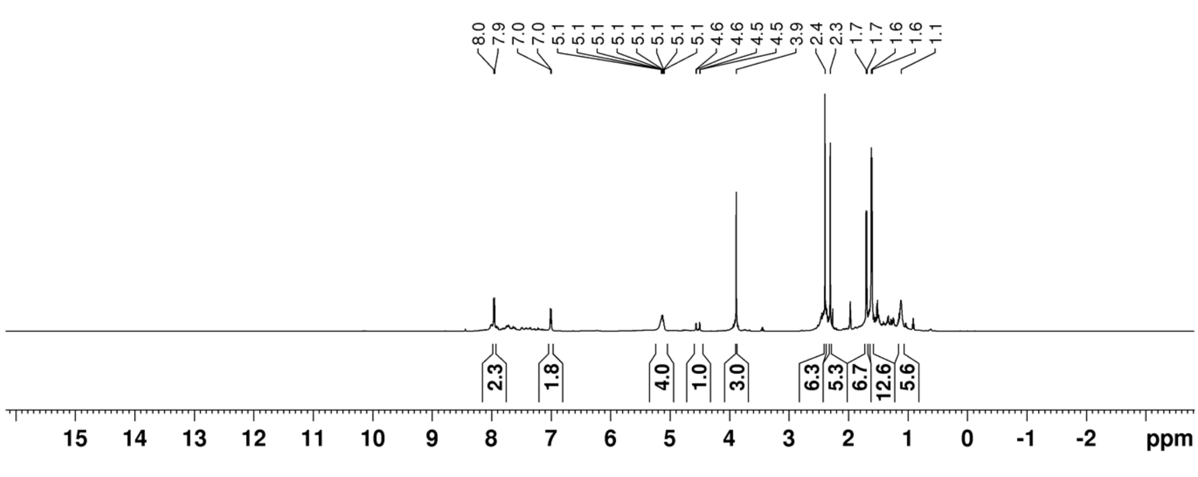

Figure S24. ^{1}H NMR spectrum of **1c**[OTf] (CD_3CN, 300 K).

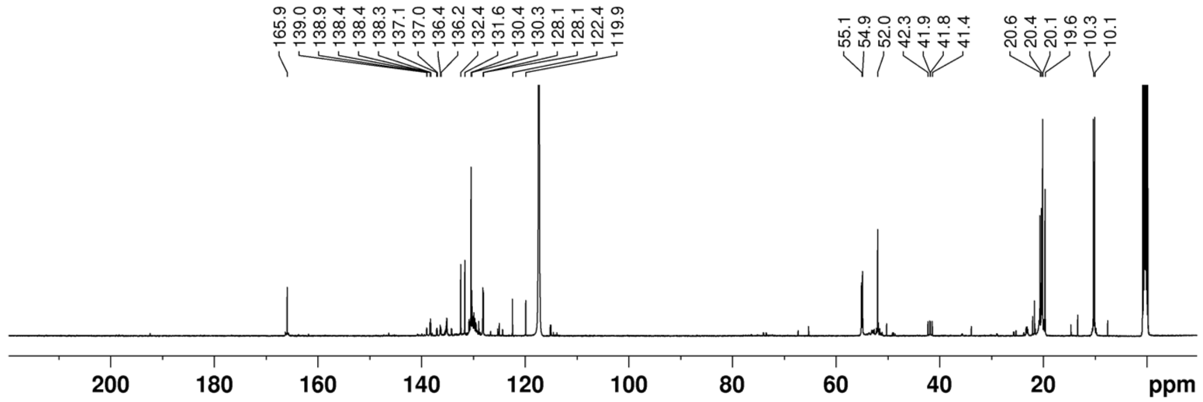

Figure S25. $^{13}C\{^{1}H\}$ NMR spectrum of **1c**[OTf] (CD_3CN, 300 K).

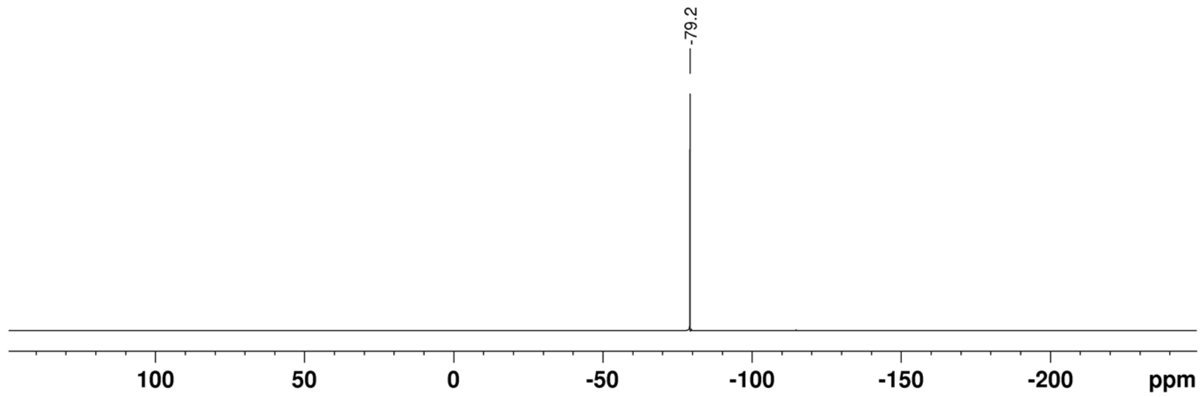

Figure S26. ^{19}F NMR spectrum of **1c**[OTf] (CD_3CN, 300 K).

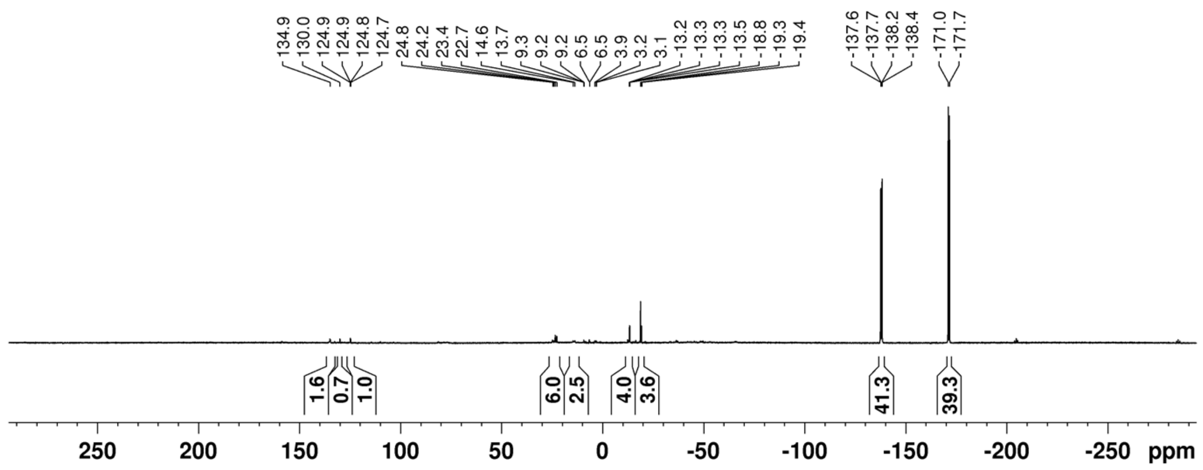

Figure S27. ^{31}P NMR spectrum of **1c**[OTf] (CD_3CN, 300 K).

S2.11 Preparation of [L_CP(OEt)C(H)(4-COOMePh)PH(L_C)][OTf]$_2$ 5c[OTf]$_2$

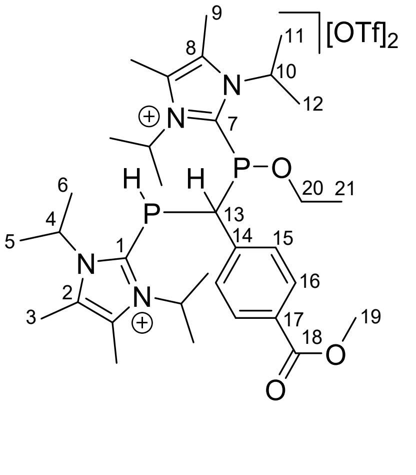

To a solid mixture of **1c**[OTf] (250 mg, 0.44 mmol, 1.0 equiv.), **3**[OTf]$_4$ (222 mg, 0.15 mmol, 0.32 equiv.), Ph_3P (12 mg, 0.04 mmol, 0.1 equiv.) and methyl 4-formylbenzoate (361 mg, 2.2 mmol, 5.0 equiv.) CH_3CN (10 ml) was added. The resulting pale-yellow solution was stirred for four days at room temperature and evaporated to dryness. Subsequently, the residue was stirred over Et_2O (10 ml) and toluene (5 ml) for 16 h before the supernatant was decanted and replaced by fresh Et_2O (15 ml) contaminated with EtOH. After stirring for another 16 h the supernatant was removed and the orange residue was dried *in vacuo*. The sticky solid was then suspended in cold (–30°C) C_6H_5F, filtered, washed with cold (–30°C) C_6H_5F (2x3 ml)

and *n*-hexane (1x10 ml), and dried *in vacuo* to afford the product as an air- and moisture-sensitive colorless solid. Single crystals suitable for single crystal X-ray diffraction analysis were obtained by diffusion of *n*-hexane into a saturated *o*-$C_6H_4F_2$ solution of the product at room temperature.

Note: The two H20 protons are anisochronous determined by crosspeaks found in 2D NMR experiments, but cannot be assigned unambiguously due to overlapping resonances in the ^{1}H NMR spectrum. The two diastereomers were obtained in a 2:1 ratio.

Yield: 192 mg (50%); **m.p.**: 148-150°C ; **Raman** (400 mW, 50 scans, 298 K, in cm^{-1}): 3078 (16), 2980 (59), 2947 (100), 2744 (5), 2334 (9), 1731 (23), 1608 (84), 1450 (33), 1416 (47), 1388 (19), 1367 (27), 1323 (6), 1281 (63), 1220 (19), 1186 (19), 1153 (16), 1112 (8), 1030 (48), 885 (14), 853 (5), 791 (11), 753 (20), 682 (9), 636 (13), 573 (13), 543 (9), 458 (11), 398 (5), 347 (20), 311 (25), 276 (17), 213 (6), 117 (11); **IR** (ATR, 298 K, in cm^{-1}): 2984 (vw), 2397 (vw), 2385 (vw), 1728 (vw), 1608 (vw), 1463 (vw), 1441 (vw), 1413 (vw), 1395 (vw), 1377 (vw), 1266 (s), 1258 (s), 1221 (w), 1184 (vw), 1145 (w), 1111 (vw), 1090 (vw), 1030 (s), 903 (vw), 877 (vw), 853 (vw), 790 (vw), 753 (vw), 696 (vw), 637 (vs), 571 (vw), 557 (vw), 543 (vw), 516 (w), 491 (vw), 478 (vw), 466 (vw), 453 (vw), 441 (vw), 426 (vw), 414 (vw); **Major Diastereomer: ^{1}H NMR** (500.13 MHz, CD_3CN, 300 K, in ppm): δ = 1.16 (2H, t, $^3J_{HH}$ = 7.0 Hz, H21), 1.28 (4H, d, $^3J_{HH}$ = 6.9 Hz, H11), 1.47-1.62 (8H, m(br), H5 and H6), 1.53 (4H, d, $^3J_{HH}$ = 7.0 Hz, H12), 2.29 (4H, s, H9), 2.39 (4H, s, H3), 3.81-3.90 (0.66H, m, H20), 3.88 (2H, s, H19), 3.97-4.06 (0.66H, m, H20), 4.43 (0.66H, *pseudo*-t(br), $^2J_{HP}$ = 5.4 Hz, $^2J_{HP}$ = 5.4 Hz, H13), 4.99 (1.33H, dsept, $^4J_{HP}$ = 6.7 Hz, $^3J_{HH}$ = 6.7 Hz, H10), 5.10 (0.66H, ddd, $^1J_{HP}$ = 250.9 Hz, $^2J_{HP}$ = 5.2 Hz, $^3J_{HH}$ = 5.2 Hz, PH), 5.12 (1.33H, m(br), H4), 7.50 (1.33H, m, H15), 8.00 (1.33H, m, H16); **^{13}C{^{1}H} NMR** (125.76 MHz, CD_3CN, 300 K, in ppm): δ = 10.1 (1.33C, s, C3), 10.3 (1.33C, s, C9), 15.8 (0.66C, d, $^3J_{CP}$ = 8 Hz, C21), 20.2 (1.33C, s, C11), 20.2-20.6 (2.66C, s, C5 and C6), 20.3 (1.33C, s, C12), 43.3 (0.66C, dd, $^1J_{CP}$ = 29 Hz, $^1J_{CP}$ = 20 Hz, C13), 52.0 (0.66C, s, C19), 52.7 (1.33C, s(br), C4), 54.2 (1.33C, d, $^3J_{CP}$ = 10 Hz, C10), 71.0 (0.66C, d, $^2J_{CP}$ = 25 Hz, C20), 121.2 (2C, q, $^1J_{CF}$ = 321 Hz, OTf), 129.5 (1.33C, *pseudo*-t, $^3J_{CP}$ = 6 Hz, $^3J_{CP}$ = 6 Hz, C15), 130.1 (0.66C, *pseudo*-t, $^5J_{CP}$ = 2 Hz, $^5J_{CP}$ = 2 Hz, C17), 130.7 (1.33C, s, C16), 131.9 (1.33C, s, C8), 132.1 (1.33C, s(br), C2), 137.3 (0.66C, dd, $^1J_{CP}$ = 46 Hz, $^3J_{CP}$ = 16 Hz, C7), 139.4 (0.66C, *pseudo*-t, $^2J_{CP}$ = 6 Hz, $^2J_{CP}$ = 6 Hz, C14), 139.8 (0.66C, dd, $^1J_{CP}$ = 73 Hz, $^3J_{CP}$ = 4 Hz, C1), 165.9 (0.66C, s, C18); **^{19}F NMR** (470.59 MHz, CD_3CN, 300 K, in ppm): δ = −79.3 (3F, s, OTf); **^{31}P NMR** (202.46 MHz, CD_3CN, 300 K, in ppm): δ = −98.2 (0.66P, dd, $^1J_{PH}$ = 238 Hz, $^2J_{PP}$ = 180 Hz, P_A), 103.2 (0.66P, d, $^2J_{PP}$ = 174 Hz, P_X); **Minor Diastereomer: ^{1}H NMR** (500.13 MHz, CD_3CN, 300 K, in ppm): δ = 1.06 (1H, t, $^3J_{HH}$ = 7.0 Hz, H21), 1.25 (2H, d, $^3J_{HH}$ = 6.9 Hz, H11), 1.47-1.62 (4H, m(br), H5 and H6), 1.50 (2H, d, $^3J_{HH}$ = 7.0 Hz, H12), 2.30 (2H, s, H9), 2.41 (2H, s, H3), 3.70-3.80 (0.33H, m, H20), 3.90 (1H, s, H19), 3.91-3.99 (0.33H, m, H20), 4.00 (0.33H, dd, $^2J_{HP}$ = 9.8 Hz, $^2J_{HP}$ = 6.9 Hz, H13), 4.69 (0.66H, m, H10), 5.10 (0.33H, ddd, $^1J_{HP}$ = 250.9 Hz, $^2J_{HP}$ = 10.1 Hz, $^3J_{HH}$ = 7.2 Hz, PH), 5.16 (0.66H, m(br), H4), 7.57 (0.66H, m, H15), 8.05 (0.66H, m, H16); **^{13}C{^{1}H} NMR** (125.76 MHz, CD_3CN, 300 K, in ppm): δ = 10.1 (0.66C, s, C3), 10.3 (0.66C, s, C9), 15.7 (0.33C, d, $^3J_{CP}$ = 8 Hz, C21), 20.2 (0.66C, s, C11), 20.2-20.6 (1.33C, s, C5 and C6), 20.3 (0.66C, s, C12), 45.1 (0.33C, dd, $^1J_{CP}$ = 29 Hz, $^1J_{CP}$ = 26 Hz, C13), 52.0 (0.33C, s, C19), 52.8 (0.66C, s(br), C4), 54.2 (0.66C, s(br), C10), 71.0 (0.33C, d, $^2J_{CP}$ = 25 Hz, C20), 129.0 (0.66C, d, $^3J_{CP}$ = 6 Hz, C15), 130.0 (0.33C, d, $^5J_{CP}$ = 1 Hz, C17), 130.7 (0.66C, s, C16), 131.9 (0.66C, s, C8), 132.1 (0.66C, s(br), C2), 137.7 (0.33C, dd, $^1J_{CP}$ = 46 Hz, $^3J_{CP}$ = 19 Hz, C7), 139.4 (0.33C, *pseudo*-t, $^2J_{CP}$ = 6 Hz, $^2J_{CP}$ = 6 Hz, C14), 140.5 (0.33C, dd, $^1J_{CP}$ = 71 Hz, $^3J_{CP}$ = 10 Hz, C1), 166.0 (0.33C, s, C18); **^{19}F NMR** (470.59 MHz, CD_3CN, 300 K, in ppm): δ = −79.3 (3F, s, OTf); **^{31}P NMR** (202.46 MHz, CD_3CN, 300 K, in ppm): δ = −83.0 (0.33P, dd, $^1J_{PH}$ = 254 Hz, $^2J_{PP}$ = 203 Hz, P_A), 100.9 (0.33P, d, $^1J_{PP}$ = 203 Hz, P_X); **elemental analysis**: calcd. for $C_{35}H_{54}F_6N_4O_9P_2S_2$: C: 45.95, H: 5.95, N: 6.12, S: 7.01; found: C: 45.49, H: 5.685, N: 6.01, S: 6.859.

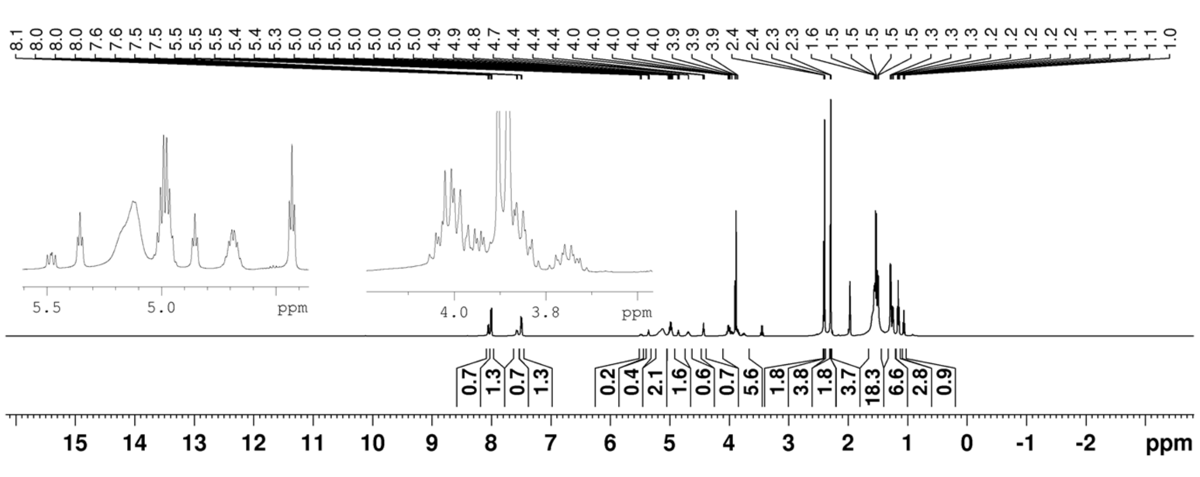

Figure S28. ^{1}H NMR spectrum of **5c**[OTf] (CD_3CN, 300 K).

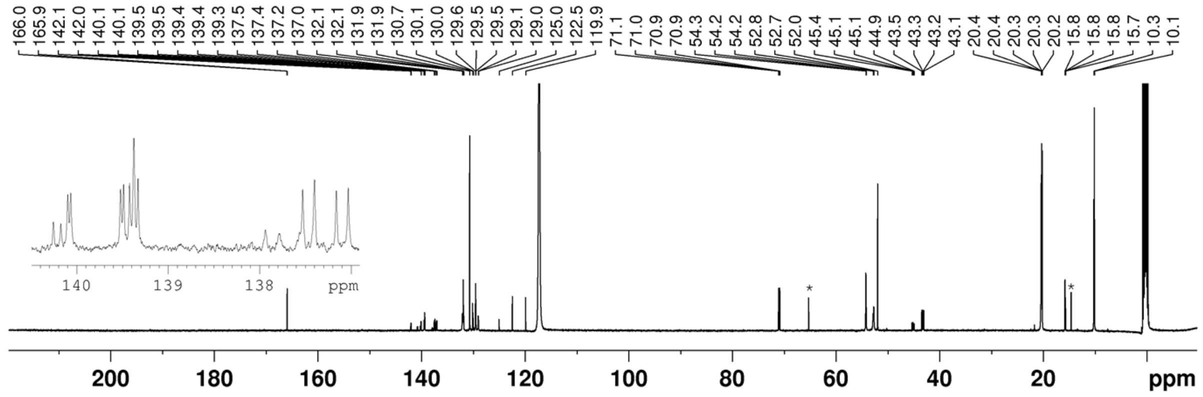

Figure S29. ^{13}C NMR spectrum of **5c**[OTf] (CD_3CN, 300 K). Asterisks mark trace impurities of Et_2O.

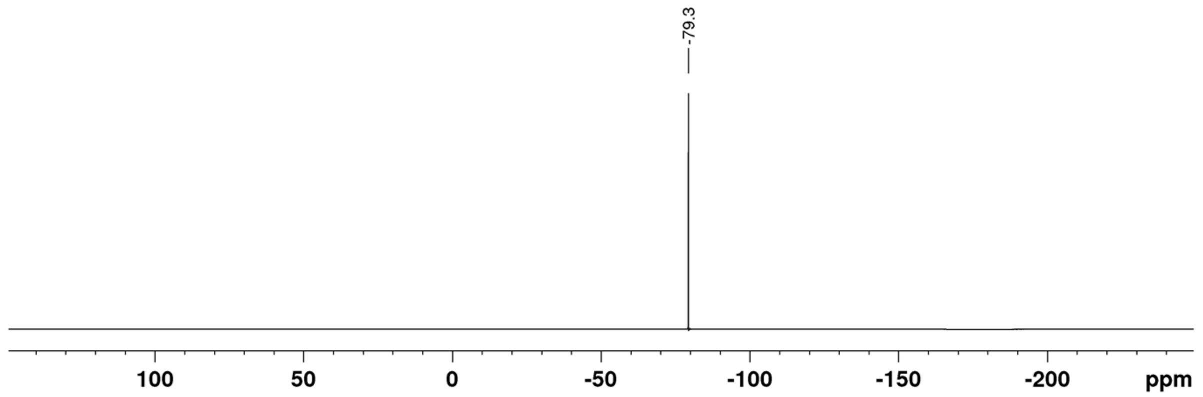

Figure S30. ^{19}F NMR spectrum of **5c**[OTf] (CD_3CN, 300 K).

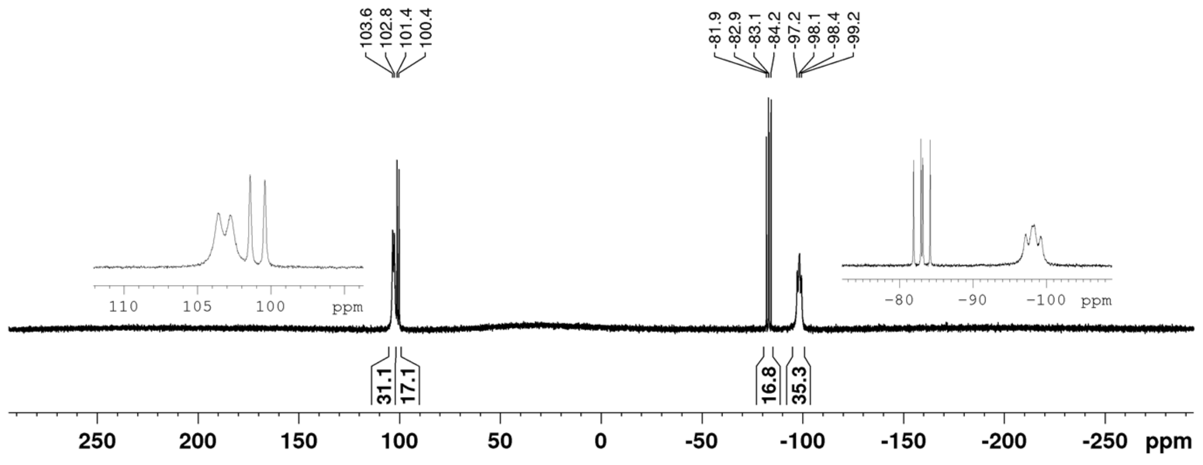

Figure S31. ^{31}P NMR spectrum of **5c**[OTf] (CD_3CN, 300 K).

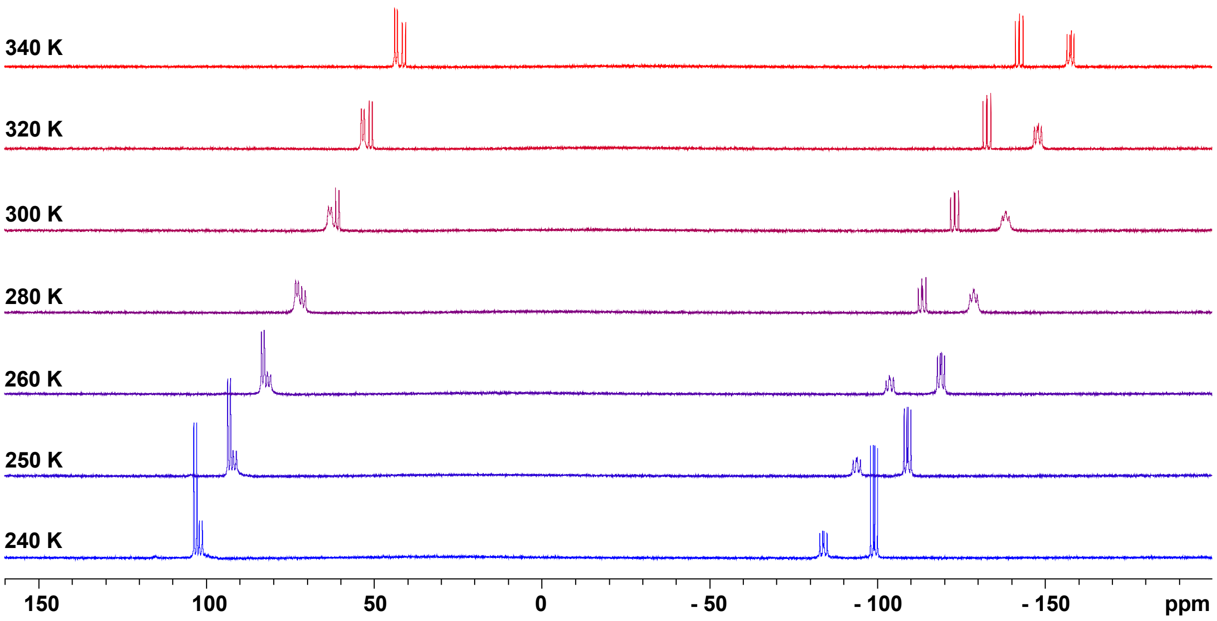

Figure S32. ^{31}P NMR spectra of the diastereomeric mixture of **5c**[OTf] at varying temperatures (240-340 K, CD_3CN). The spectra are offset by 10 ppm.

S2.12 Preparation of [(*E*)-(L_C)P=C(H)C_6F_5][OTf] (2h[OTf])

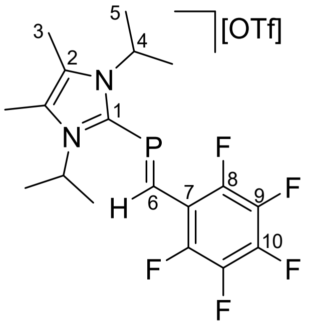

To a solution of **1c**[OTf] (2.00 g, 3.5 mmol, 1.0 equiv.) in CH_3CN (10 ml), a solution of 2,3,4,5,6-pentafluorobenzaldehyde (828 mg, 4.2 mmol, 1.2 equiv.) in CH_3CN (4 ml) was added. The pale-yellow solution was stirred for 16 hours at room temperature and all volatiles were subsequently removed *in vacuo*. The resulting, orange-colored oil was stirred vigorously over Et_2O (3x10 ml) and dried *in vacuo* to obtain the product as an off-white air- and moisture-sensitive solid. Colorless single crystals were obtained by slow diffusion of Et_2O into saturated CH_3CN solution at room temperature or −30°C.

Yield: 1.63 g (86%), **m.p.**: 170-172 °C; **Raman** (100 mW, 100 scans, 298 K, in cm^{-1}): 2992 (10), 2944 (17), 1650 (48), 1521 (19), 1439 (26), 1413 (100), 1341 (33), 1294 (35), 1273 (23), 1254 (22), 1223 (16), 1163 (36), 1068 (15), 1024 (42), 988 (15), 893 (17), 861 (16), 828 (16), 794 (17), 752 (18), 673 (14), 646 (15), 620 (16), 575 (23), 539 (16), 483 (22), 445 (19), 395 (20), 348 (18), 312 (19), 283 (17), 256 (16), 211 (15), 113 (21), 78 (38); **IR** (ATR, 298 K, in cm^{-1}): 3007 (vw), 2941 (vw), 2363 (vw), 2340 (vw), 1648 (vw), 1628 (vw), 1522 (w), 1504 (s), 1471 (vw), 1411 (w), 1376 (vw), 1342 (vw), 1266 (vs), 1223 (w), 1143 (m), 1117 (vw), 1030 (s), 989 (s), 903 (vw), 858 (vw), 829 (vw), 792 (vw), 768 (vw), 751 (vw), 725 (vw), 687 (vw), 636 (vs), 602 (vw), 571 (vw), 537 (vw), 516 (w), 483 (vw), 457 (vw), 446 (vw), 426 (vw); **^{1}H NMR** (500.13 MHz, CD_3CN, 300 K, in ppm): δ = 1.58 (12H, d, ^3J(HH) = 7.0 Hz, H5), 2.41 (6H, s, H3), 4.88 (2H, dsept, ^3J(HH) = 6.9 Hz, ^4J(HP) = 2.9 Hz, H4), 9.50 (1H, d, ^2J(HP) = 23.1 Hz, H6); **^{13}C{^{1}H} NMR** (125.76 MHz, CD_3CN, 300 K, in ppm): δ = 9.7 (2C, s, C3), 20.6 (4C, s, C5), 53.6 (2C, d, ^3J(CP) = 4 Hz, C4), 114.0 (1C, m, C7), 121.2 (1C, q, ^1J(CF) = 321 Hz, OTf), 130.1 (2C, s, C2), 138.8 (2C, m, C9), 143.5 (1C, m, C10), 144.5 (2C, m, C8), 145.3 (1C, dt, ^1J(CP) = 79 Hz, ^5J(CF) = 7 Hz, C1), 175.1 (1C, d, ^1J(CP) = 46 Hz, C6); **^{19}F NMR** (470.59 MHz, CD_3CN, 300 K, in ppm): δ = −163.6 (2F, m, F9), −151.2 (1F, *pseudo*-qt, ^3J(FF) = 20 Hz, ^6J(FP) = 20 Hz, ^4J(FF) = 4 Hz, F10), −134.9 (2F, m, F8), −79.2 (3F, s, OTf); **^{31}P NMR** (202.46 MHz, CD_3CN, 300 K, in ppm): δ = 214.4 (1P, *pseudo*-tt, ^4J(PF) = 167 Hz, ^2J(PH) = 20 Hz, ^6J(PF) = 20 Hz, P); **elemental analysis**: calcd. for $C_{19}H_{21}F_8N_2O_3PS$: C: 42.23, H: 3.92, N: 5.18, S: 5.93; found: C: 42.24, H: 4.085, N: 5.09, S: 5.848.

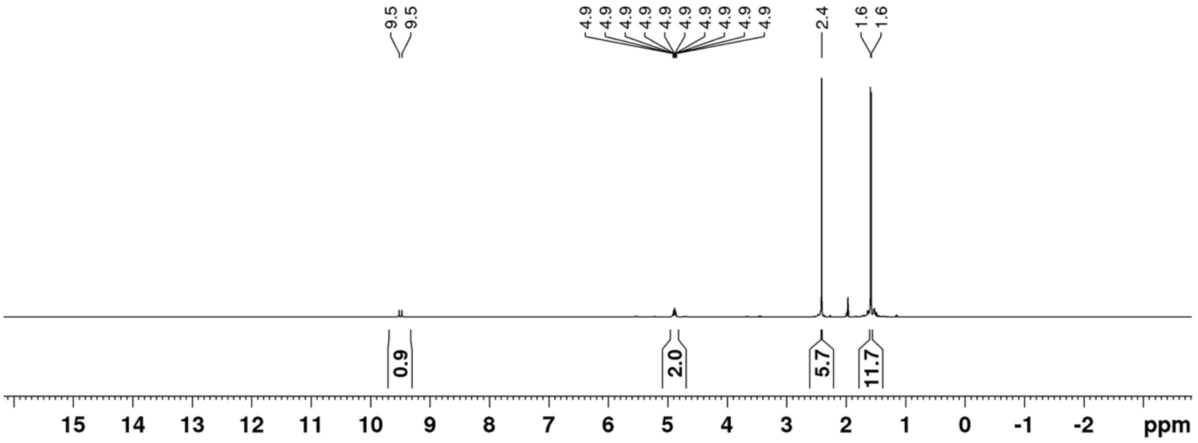

Figure S33. ^{1}H NMR spectrum of **2f**[OTf] (CD_3CN, 300 K).

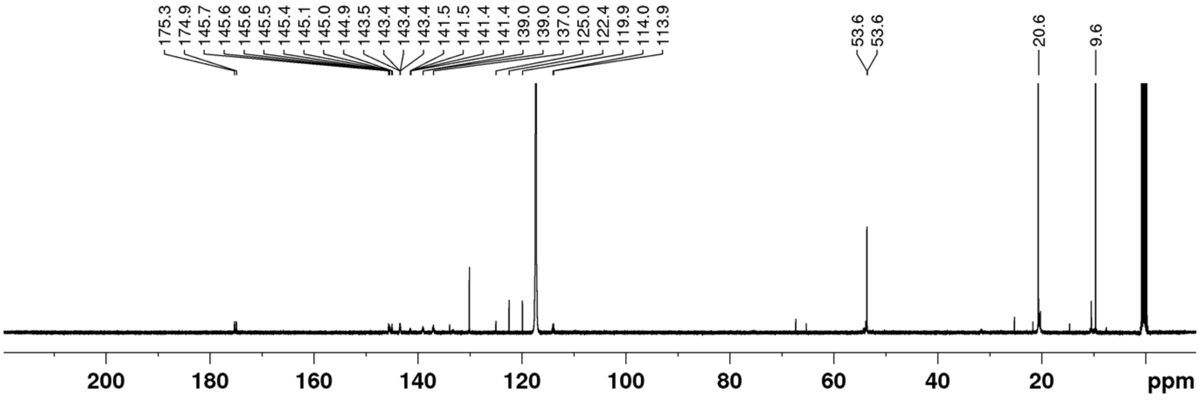

Figure S34. ^{13}C NMR spectrum of **2f**[OTf] (CD_3CN, 300 K).

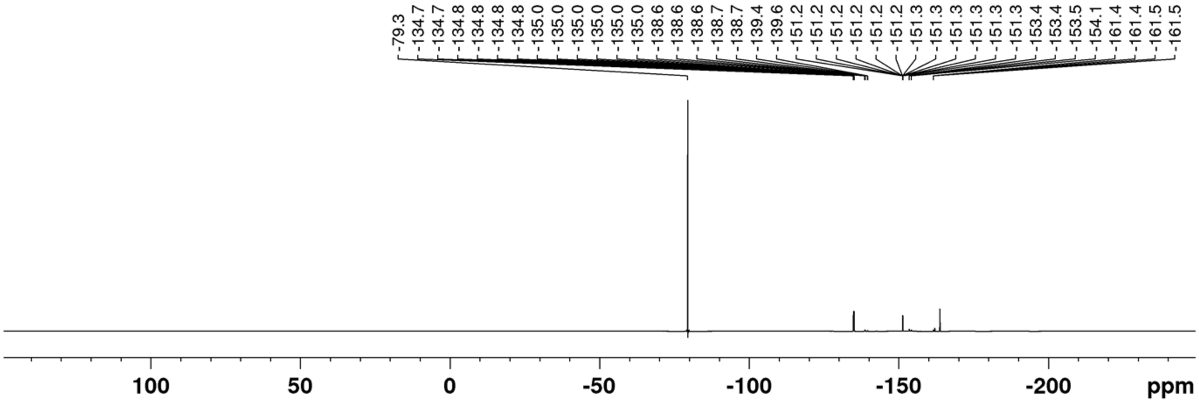

Figure S35. ^{19}F NMR spectrum of **2f**[OTf] (CD_3CN, 300 K).

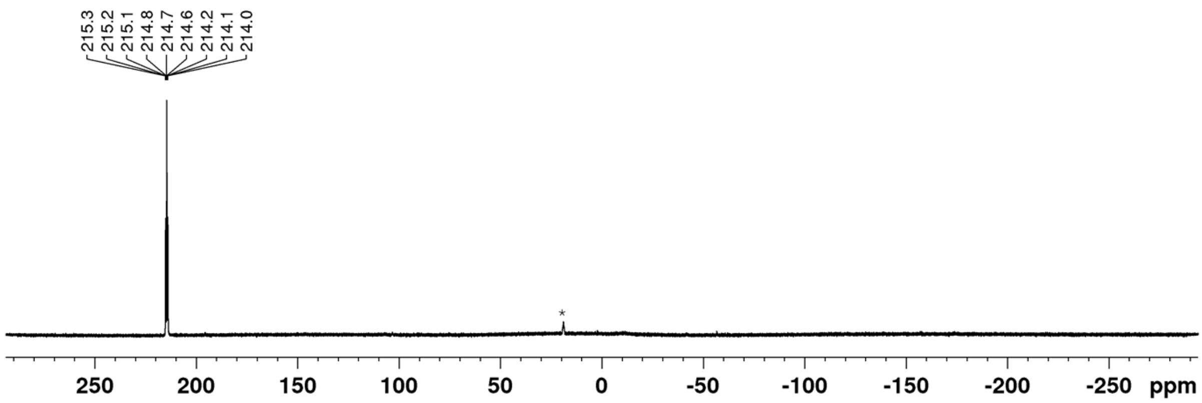

Figure S36. ^{31}P NMR spectrum of **2f**[OTf] (CD_3CN, 300 K). Asterisk marks traces of [*anti*-($\mathbf{2h}$)$_2$]$^{2+}$.

S2.13 Preparation of [(*E*)-(L_C)P=C(H)^{t}Bu][OTf] (2i[OTf])

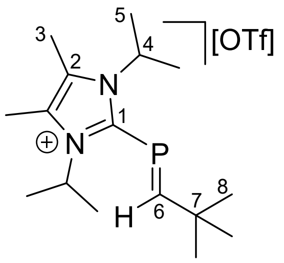

To a solution of **1c**[OTf] (500 mg, 0.88 mmol, 1.0 equiv.) in CH_3CN (5 ml), 2,2-dimethylpropionaldehyde (956 µl, 758 mg, 8.8 mmol, 10.0 equiv.) was added while stirring. The resulting pale-yellow solution was stirred for seven days at room temperature and evaporated to dryness. Subsequent washing of the residue with Et_2O (2x4 ml) and *n*-hexane (2x4 ml) and consecutive drying *in vacuo* afforded the product as an air- and moisture-sensitive off-white solid (287 mg). A second batch of analytically pure colorless product precipitated from the Et_2O/*n*-hexane wash solution after 16 hours at room temperature, was filtered off, and dried *in vacuo* (64.0 mg).

Yield: 352 mg (93%); **m.p.**: 85-87°C ; **Raman** (100 mW, 100 scans, 298 K, in cm^{-1}): 2979 (52), 2950 (78), 2910 (47), 2816 (5), 2783 (9), 2766 (7), 2747 (7), 2715 (7), 1625 (21), 1451 (65), 1431 (56), 1405 (31), 1380 (27), 1344 (11), 1295 (37), 1269 (15), 1223 (14), 1193 (16), 1173 (9), 1154 (14), 1136 (16), 1092 (7), 1033 (100), 1020 (47), 981 (6), 937 (16), 930 (16), 890 (25), 827 (6), 794 (12), 753 (59), 728 (6), 701 (11), 672 (10), 653 (9), 637 (7), 585 (21), 572 (20), 550 (17), 505 (14), 459 (19), 417 (14), 397 (11), 347 (27), 320 (23), 310 (32); **IR** (ATR, 298 K, in cm^{-1}): 2979 (vw), 2962 (vw), 2907 (vw), 2869 (vw), 1622 (vw), 1468 (vw), 1453 (w), 1425 (w), 1394 (vw), 1377 (vw), 1366 (vw), 1264 (vs), 1221 (m), 1173 (vw), 1139 (s), 1116 (w), 1092 (w), 1030 (vs), 938 (vw), 910 (vw), 888 (vw), 872 (vw), 840 (vw), 825 (vw), 792 (vw), 751 (w), 700 (vw), 635 (vs), 570 (w), 516 (m), 458 (vw), 442 (vw), 415 (w); **^{1}H NMR** (500.13 MHz, THF-D_8, 300 K, in ppm): δ = 1.31 (9H, d, ^4J(HP) = 1.9 Hz, H8), 1.61 (12H, d, ^3J(HH) = 7.0 Hz, H5), 2.42 (6H, s, H3), 4.86 (2H, dsept, ^3J(HH) = 7.0 Hz, ^4J(HP) = 2.5 Hz, H4), 9.79 (1H, d, ^2J(HP) = 22.9 Hz, H6); **^{13}C{^{1}H} NMR** (125.76 MHz, THF-D_8, 300 K, in ppm): δ = 9.5 (2C, s, C3), 20.6 (4C, s, C5), 28.5 (3C, d, ^3J(CP) = 14 Hz, C8), 40.9 (1C, d, ^2J(CP) = 16 Hz, C7), 53.1 (2C, d, ^3J(CP) = 3 Hz, C4), 122.2 (1C, q, ^1J(CF) = 321 Hz, OTf), 129.7 (2C, s, C2), 145.7 (1C, d, ^1J(CP) = 83 Hz, C1), 219.3 (1C, d, ^1J(CP) = 46 Hz, C6); **^{19}F{^{1}H} NMR** (470.59 MHz, THF-D_8, 300 K, in ppm): δ = −79.0 (3F, s, OTf); **^{31}P NMR** (202.46 MHz, THF-D_8, 300 K, in ppm): δ = 171.4 (1P, d, ^2J(PH) = 19 Hz, P); **elemental analysis**: calcd. for $C_{17}H_{30}F_3N_2O_3PS$: C: 47.43, H: 7.02, N: 6.51, S: 7.45; found: C: 47.03, H: 6.823, N: 6.69, S: 7.509.

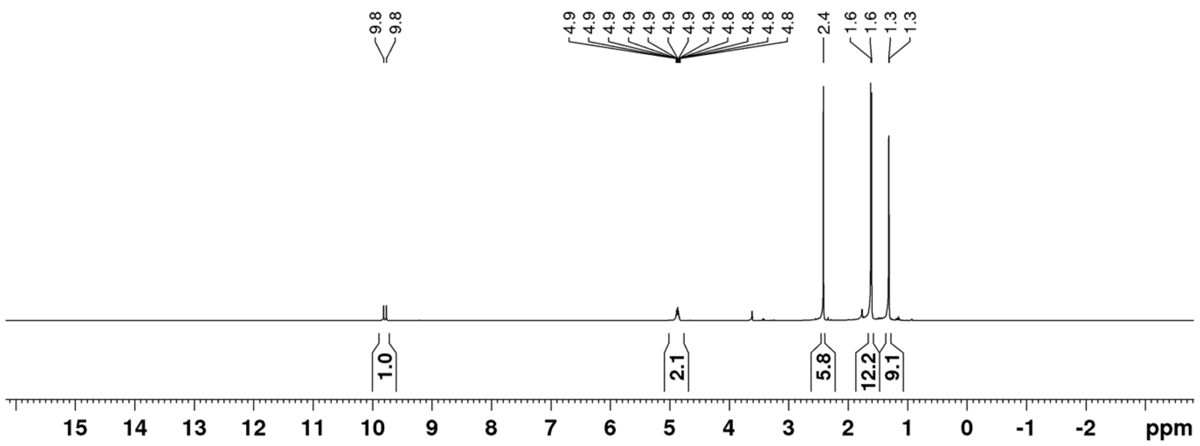

Figure S37. ^{1}H NMR spectrum of **2g**[OTf] (THF-D_8, 300 K).

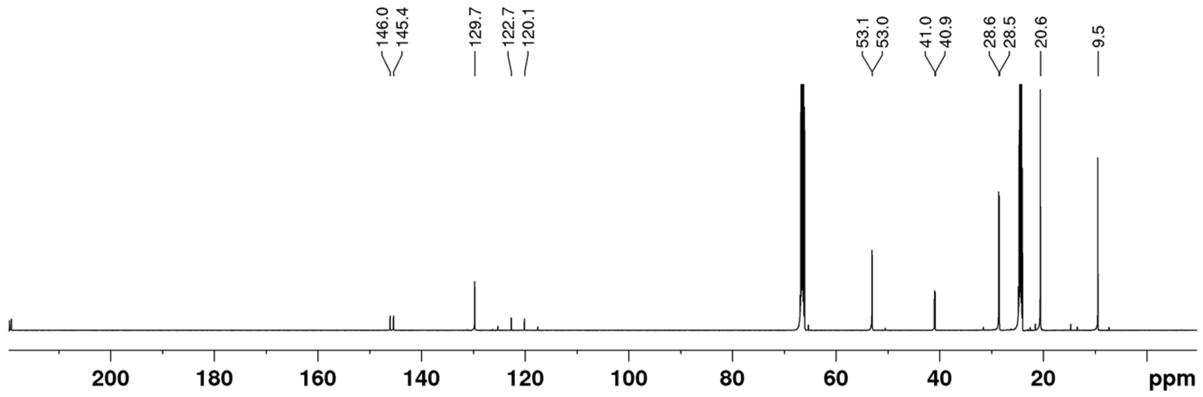

Figure S38. ^{13}C NMR spectrum of **2g**[OTf] (THF-D_8, 300 K).

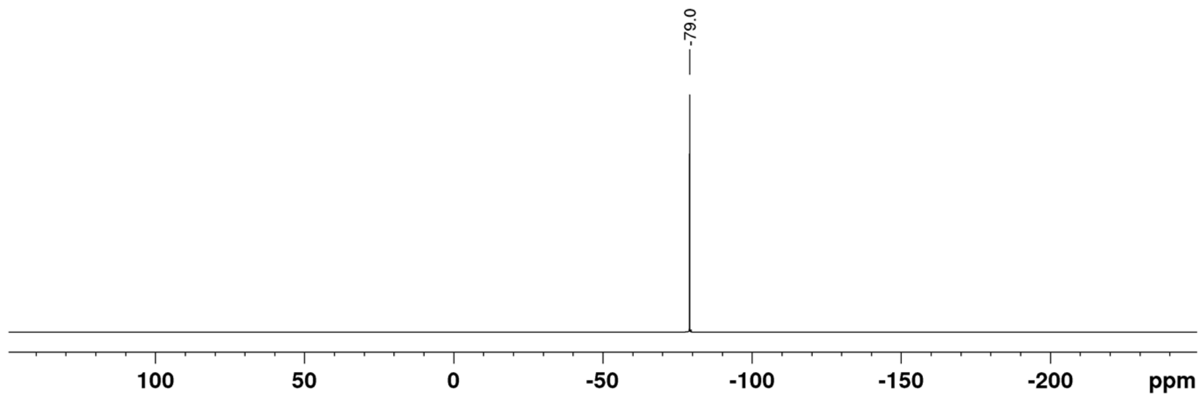

Figure S39. ^{19}F NMR spectrum of **2g**[OTf] (THF-D_8, 300 K).

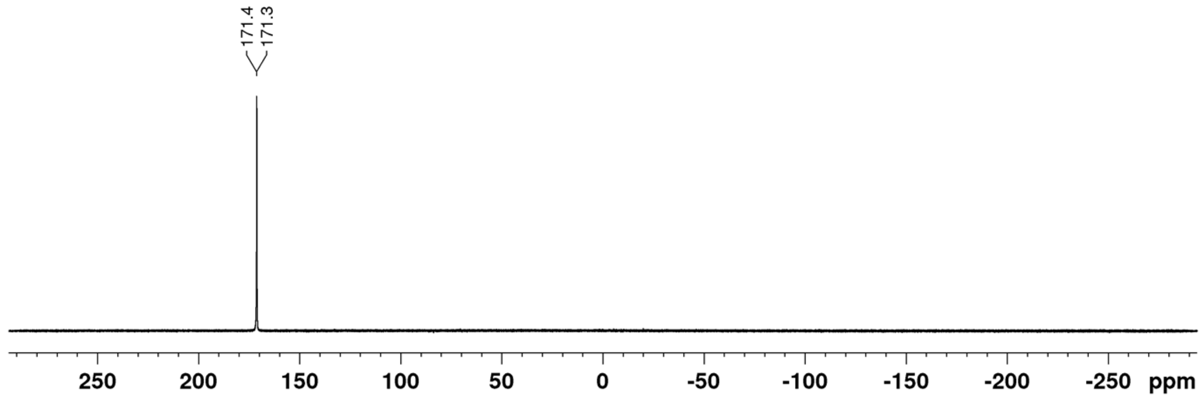

Figure S40. ^{31}P NMR spectrum of **2g**[OTf] (THF-D_8, 300 K).

S2.14 Preparation of 1,2-di(imidazoliumyl)-3-pentafluorophenyldiphosphirane 4h[OTf]$_2$

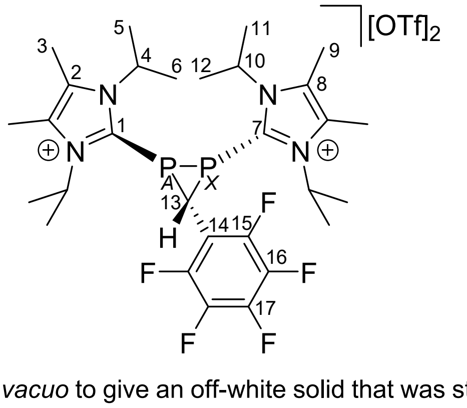

To a solid mixture of **1c**[OTf] (250 mg, 0.44 mmol, 1.0 equiv.), **3**[OTf]$_4$ (159 mg, 0.11 mmol, 0.25 equiv.), Ph_3P (16 mg, 0.04 mmol, 0.15 equiv.) and pentafluorophenylaldehyde (172 mg, 0.88 mmol, 2.0 equiv.) CH_3CN (10 ml) was added. The resulting pale-yellow solution was stirred for three days at room temperature, analyzed by multinuclear NMR analysis (**Figure S41**) and evaporated to dryness. Subsequently, the residue was stirred over Et_2O (10 ml) and toluene (5 ml) for 16 h before the supernatant was decanted and replaced by fresh Et_2O (15 ml) and stirred for another 16 h at room temperature. The resulting solid was suspended in C_6H_5F (5 ml), filtered and washed with C_6H_5F (2 x 4 ml) and *n*-pentane (10 ml) to give a crude solid containing a mixture of **4h**[OTf]$_2$ and **6h**[OTf]$_3$ (239 mg, **Figure S42**). This solid was extracted with THF (3 x 20 ml) and the liquid fractions were combined and evaporated to dryness *in vacuo* to give an off-white solid that was stirred over Et_2O (5 ml) for one hour and again dried *in vacuo* to afford the product

as an air- and moisture sensitive off-white solid (175 mg). The extraction residue was used for the isolation of **6h**[OTf]$_3$ (see **S2.14**). Colorless single crystals were obtained by slow diffusion of *n*-pentane into a saturated THF solution at room temperature.

Yield: 175 mg (44%); **m.p.**: 200-202°C (decomp.); **IR** (ATR, 298 K, in cm^{-1}): 2995 (vw), 2950 (vw), 1657 (vw), 1620 (vw), 1524 (w), 1502 (w), 1466 (vw), 1446 (vw), 1416 (vw), 1397 (vw), 1379 (vw), 1329 (vw), 1262 (vs), 1221 (w), 1148 (s), 1117 (vw), 1089 (vw), 1030 (s), 1004 (w), 977 (w), 937 (vw), 906 (vw), 885 (vw), 806 (vw), 790 (vw), 753 (vw), 702 (vw), 636 (vs), 572 (w), 547 (vw), 517 (m), 490 (vw), 464 (vw), 414 (vw); **^{1}H NMR** (500.13 MHz, CD_3CN, 300 K, in ppm): δ = 1.34 (6H, s(br), H11), 1.60 (6H, d(br), $^3J_{HH}$ = 6.9 Hz, H12), 1.63 (6H, d, $^3J_{HH}$ = 6.9 Hz, H5), 1.67 (6H, d, $^3J_{HH}$ = 7.0 Hz, H6), 2.35 (6H, s, H3), 2.40 (6H, s, H9), 4.43 (1H, dd, $^2J_{HP}$ = 30.5 Hz, $^2J_{HP}$ = 3.3 Hz, H13), 5.11 (2H, dsept, $^3J_{HH}$ = 6.9 Hz, $^4J_{HP}$ = 2.1 Hz, H4), 5.14 (2H, s(br), H10); **^{13}C{^{1}H} NMR** (125.76 MHz, CD_3CN, 300 K, in ppm): δ = 10.1 (2C, s, C9), 10.3 (2C, s, C3), 19.8 (2C, s, C11), 20.0 (2C, s, C6), 20.3 (2C, d, $^4J_{CP}$ = 3 Hz, C5), 20.4 (2C, d, $^4J_{CP}$ = 2 Hz, C12), 31.8 (1C, dd, $^1J_{CP}$ = 61 Hz, $^1J_{CP}$ = 52 Hz, C13), 54.9 (2C, *pseudo*-t, $^3J_{CP}$ = 4 Hz, $^4J_{CP}$ = 4 Hz, C4), 55.1 (2C, dd, $^3J_{CP}$ = 7 Hz, $^4J_{CP}$ = 5 Hz, C10), 108.1 (1C, m, C14), 121.1 (2C, q, $^1J_{CF}$ = 321 Hz, OTf), 131.2 (2C, s, C8), 132.6 (2C, s, C2), 137.0 (1C, dd, $^1J_{CP}$ = 93 Hz, $^2J_{CP}$ = 12 Hz, C7), 138.3 (2C, m, C16), 138.8 (1C, m, C1), 141.1 (1C, m, C17), 145.8 (2C, m, C15); **^{19}F NMR** (470.59 MHz, CD_3CN, 300 K, in ppm): δ = -161.7 (2F, m, F8), -153.9 (1F, t, $^3J_{FF}$ = 21 Hz, F10), -140.7 (2F, m, F9), -79.3 (6F, s, OTf); **^{31}P NMR** (202.46 MHz, CD_3CN, 300 K, in ppm): δ = -173.1 (1P, d, $^1J_{PP}$ = 125 Hz, $^4J_{PF}$ = 78 Hz, P$_A$), -157.2 (1P, dd, $^1J_{PP}$ = 127 Hz, $^2J_{PH}$ = 31 Hz, P$_X$); **elemental analysis**: calcd. for $C_{31}H_{41}F_{11}N_4O_6P_2S_2$: C: 41.34, H: 4.59, N: 6.22, S: 7.12; found: C: 41.34, H: 4.776, N: 6.17, S: 6.822.

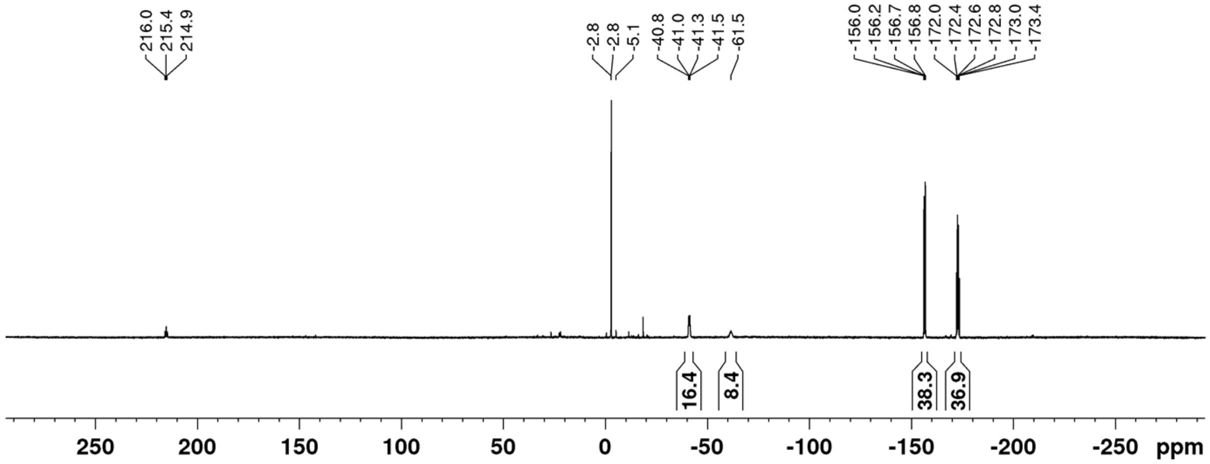

Figure S41. ^{31}P NMR spectrum of the converted reaction mixture after 3 days at room temperature (C_6D_6 cap., CH_3CN, 300 K).

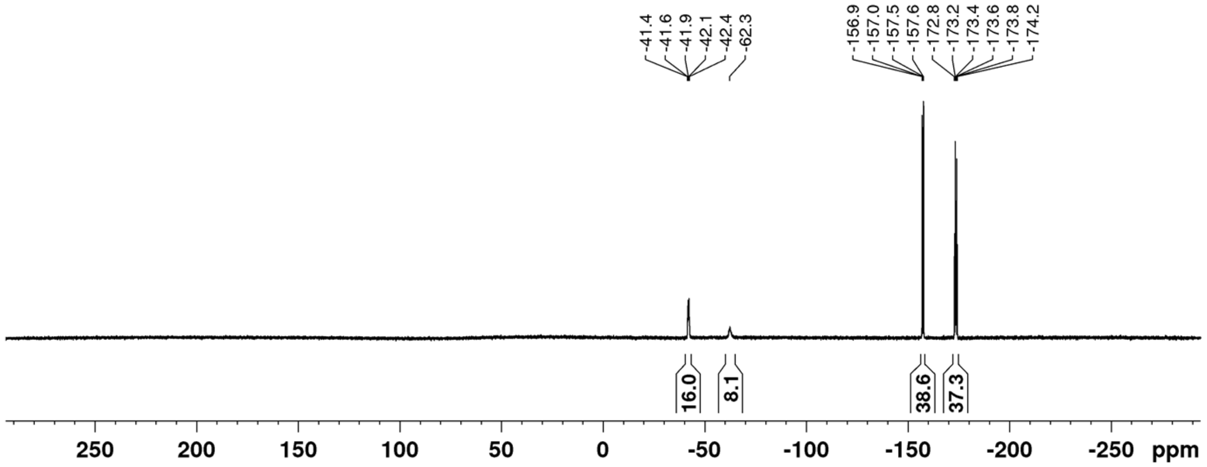

Figure S42. ^{31}P NMR spectrum of the separated crude solid containing **4h**[OTf]$_2$ and **6h**[OTf]$_3$ in a 9:2 ratio (CD_3CN, 300 K).

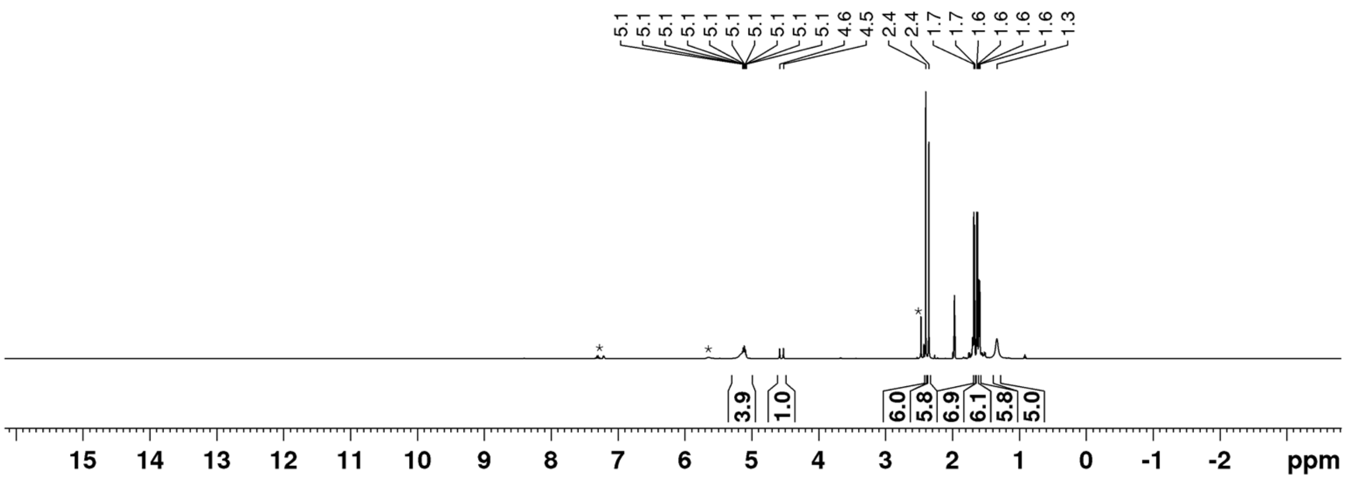

Figure S43. ^{1}H NMR spectrum of **4h**[OTf]$_2$ (CD_3CN, 300 K). Asterisks marks trace amounts of C_6H_5F solvent and impurity with **6h**[OTf]$_3$.

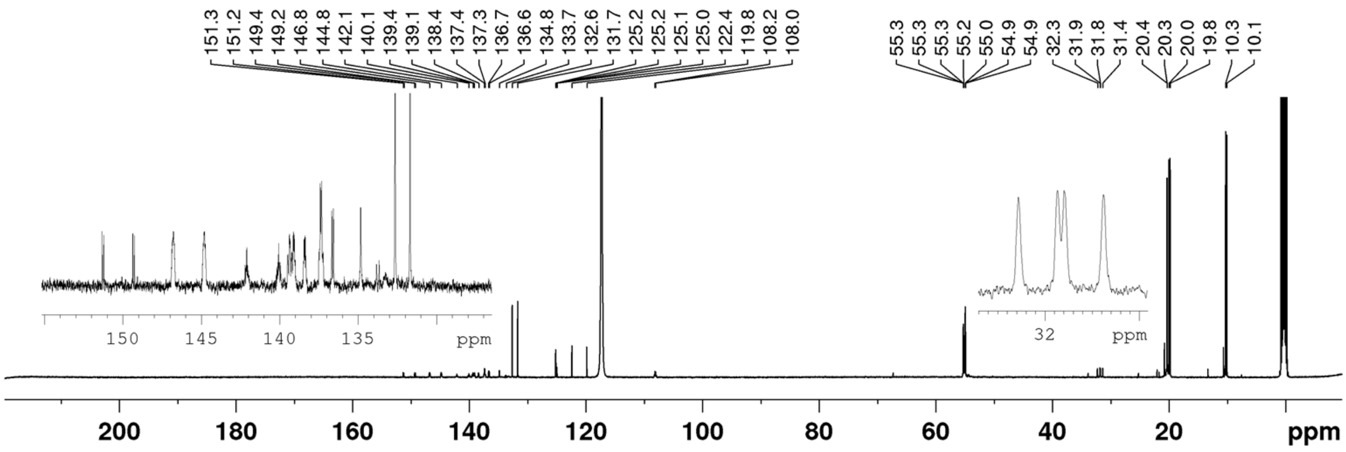

Figure S44. $^{13}C\{^{1}H\}$ NMR spectrum of **4h**[OTf]$_2$ (CD_3CN, 300 K).

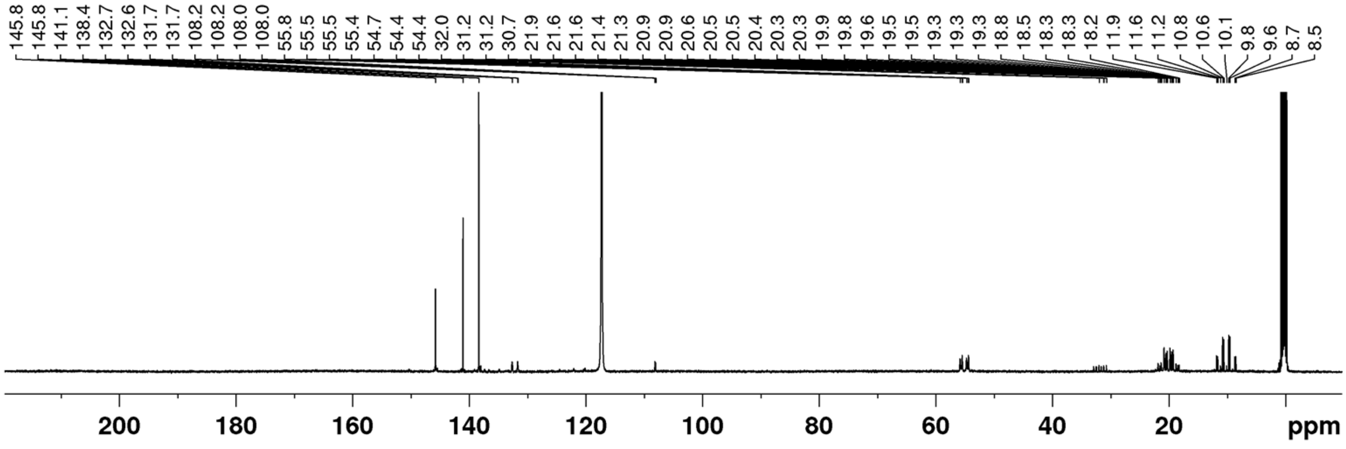

Figure S45. $^{13}C\{^{19}F\}$ NMR spectrum of **4h**[OTf]$_2$ (CD_3CN, 300 K).

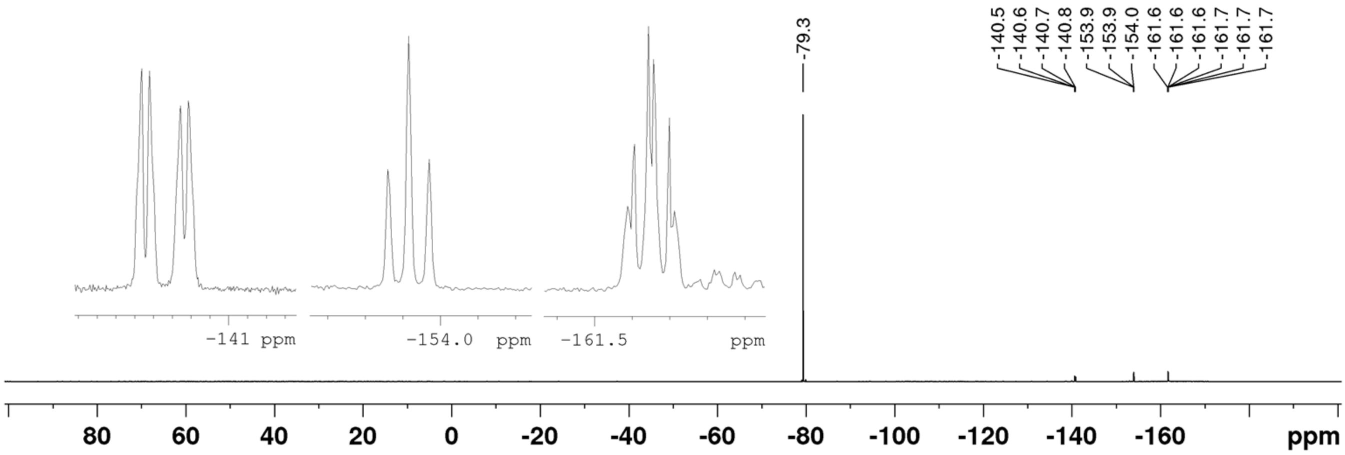

Figure S46. ^{19}F NMR spectrum of **4h**[OTf]$_2$ (CD_3CN, 300 K).

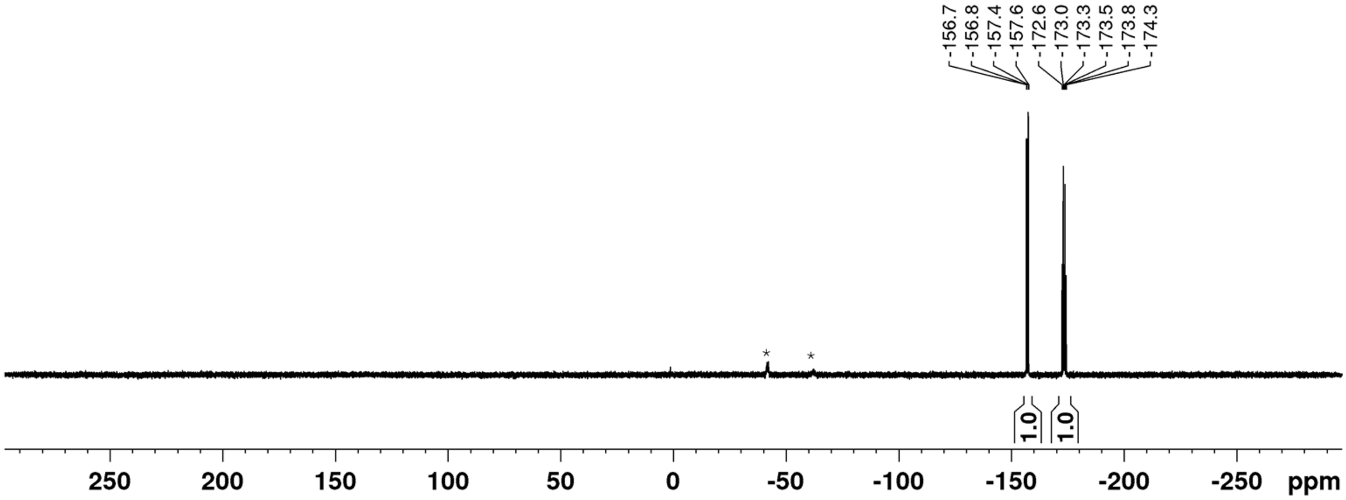

Figure S47. ^{31}P NMR spectrum of **4h**[OTf]$_2$ (CD_3CN, 300 K). Asterisks marks trace impurity with **6h**[OTf]$_3$.

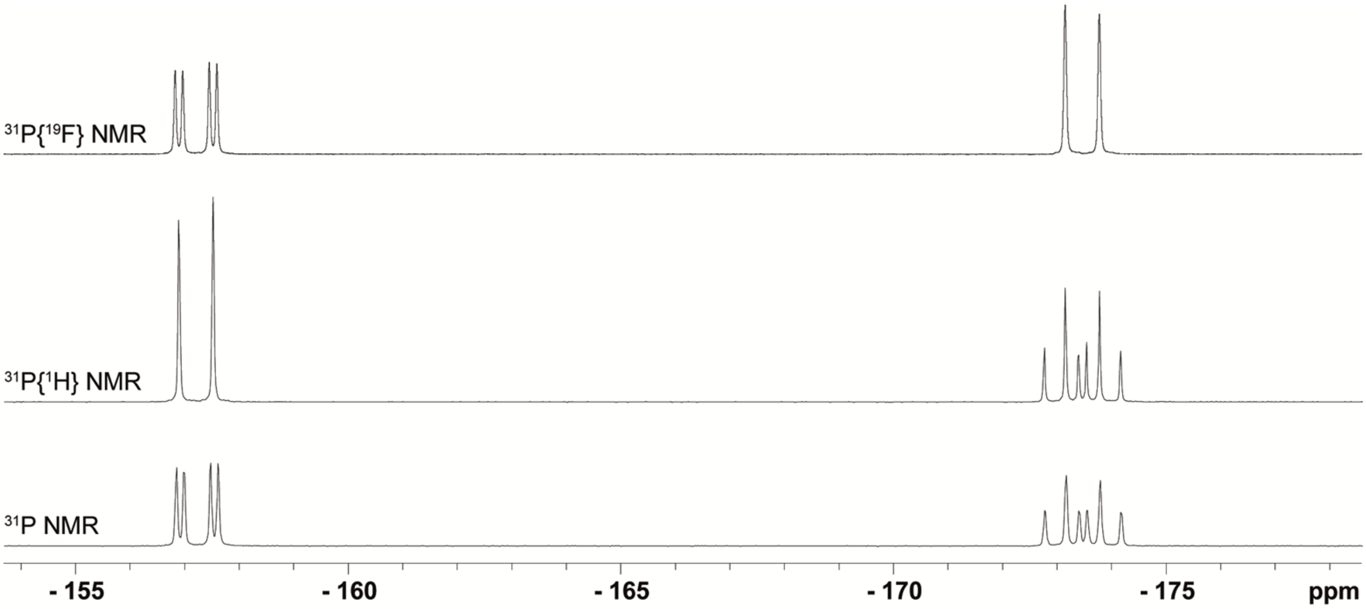

Figure S48. ^{31}P, ^{31}P{^{1}H}, and ^{31}P{^{19}F} NMR spectra of **4h**[OTf]$_2$ (CD_3CN, 300 K).

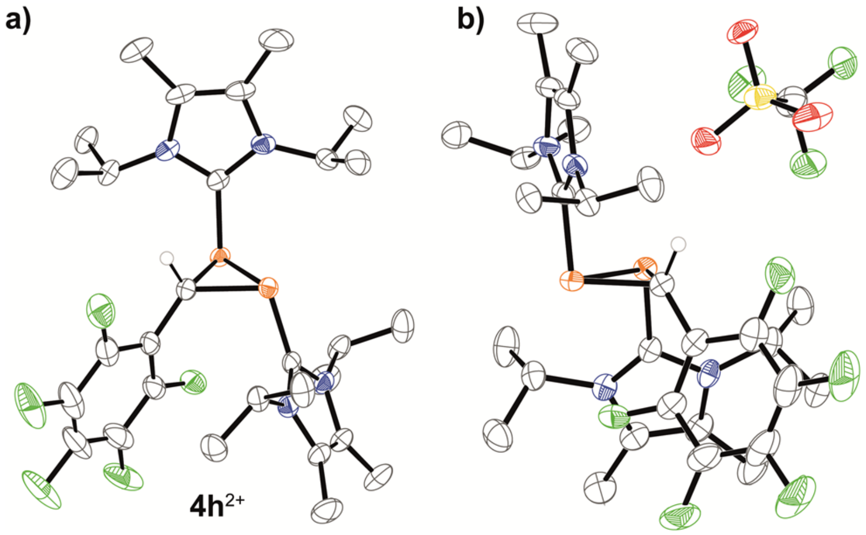

Figure S49. a) Molecular structure of **4h**$^{2+}$ in **4h**[OTf]$_2$ and **b)** OTf contact with the H atom of **4h**$^{2+}$ in the molecular structure of **4h**[OTf]$_2$; selected hydrogen atoms and anions are omitted for clarity, and thermal ellipsoids are displayed at 50% probability (100 K).

S2.15 Preparation of 1,2,3-tri(imidazoliumyl)-4-pentafluorophenyltriphosphetane 6h[OTf]$_3$

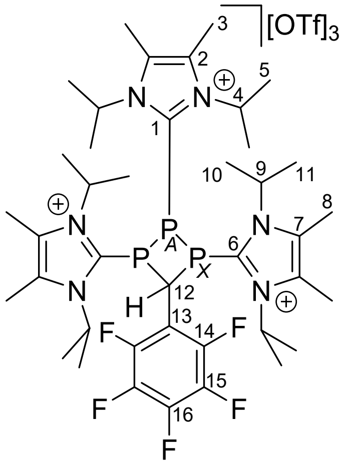

To a solid mixture of **1c**[OTf] (250 mg, 0.44 mmol, 1.0 equiv.), **3**[OTf]$_4$ (159 mg, 0.11 mmol, 0.25 equiv.), Ph_3P (16 mg, 0.04 mmol, 0.15 equiv.) and pentafluorophenylaldehyde (172 mg, 0.88 mmol, 2.0 equiv.) CH_3CN (10 ml) was added. The resulting pale-yellow solution was stirred for three days at room temperature, analyzed by multinuclear NMR analysis (**Figure S41**) and evaporated to dryness. Subsequently, the residue was stirred over Et_2O (10 ml) and toluene (5 ml) for 16 h before the supernatant was decanted and replaced by fresh Et_2O (15 ml) and stirred for another 16 h at room temperature. The resulting solid was suspended in C_6H_5F (5 ml), filtered and washed with C_6H_5F (2 x 4 ml) and *n*-pentane (10 ml) to give a crude solid containing a mixture of **4h**[OTf]$_2$ and **6h**[OTf]$_3$ (239 mg, **Figure S42**). This solid was extracted with THF (3 x 20 ml) and the liquid fractions were combined and used in the isolation of **4h**[OTf]$_2$ (see **S2.13**). The extraction residue was washed with *n*-hexane (10 ml) and dried *in vacuo* to afford the product as an air- and moisture sensitive off-white solid (36 mg). Colorless single crystals were obtained by layering a saturated C_6H_5F solution with *n*-hexane at room temperature.

Yield: 36 mg (20%); 160-162°C (decomp.); **IR** (ATR, 298 K, in cm^{-1}): 2992 (vw), 2946 (vw), 2358 (vw), 1609 (vw), 1523 (vw), 1503 (w), 1464 (vw), 1398 (vw), 1380 (vw), 1359 (vw), 1266 (s), 1251 (s), 1222 (w), 1150 (m), 1123 (vw), 1088 (vw), 1030 (s), 992 (vw), 972 (w), 905 (vw), 792 (vw), 754 (vw), 686 (vw), 637 (vs), 615 (vw), 600 (vw), 572 (vw), 544 (vw), 517 (w), 441 (vw), 412 (vw); **^{1}H NMR** (500.13 MHz, CD_3CN, 300 K, in ppm): δ = 1.56 (12H, d, $^3J_{HH}$ = 7.0 Hz, H10), 1.71 (12H, d, $^3J_{HH}$ = 7.0 Hz, H11), 1.75 (12H, d, $^3J_{HH}$ = 7.0 Hz, H5), 2.43 (12H, s, H8), 2.47 (6H, s, H3), 5.03 (1H, *pseudo*-q, $^2J_{HP}$ = 7.6 Hz, $^3J_{HP}$ = 7.6 Hz, H12), 5.58 (4H, s(br), H9), 5.90 (2H, m(br), H4);**^{13}C{^{1}H} NMR** (125.76 MHz, CD_3CN, 300 K, in ppm): δ = 10.3 (2C, s, C3), 10.5 (4C, s, C8), 20.3 (8C, s, C10 and C11), 20.8 (4C, s, C5), 31.2 (1C, m, C12), 54.4 (4C, m, C9), 54.8 (2C, dt, $^3J_{CP}$ = 14 Hz, $^4J_{CP}$ = 12 Hz, C4), 110.7 (1C, m, C13), 121.0 (3C, q, $^1J_{CF}$ = 321 Hz, OTf), 133.7 (2C, s, C2), 133.8 (4C, s, C7), 134.4 (2C, m, C6), 135.1 (1C, dt, $^1J_{CP}$ = 70 Hz, $^2J_{CP}$ = 18 Hz, C1), 138.0 (2C, m, C15), 141.4 (1C, m, C16), 145.5 (2C, m, C14); **^{19}F NMR** (470.59 MHz, CD_3CN, 300 K, in ppm): δ = -161.8 (2F, m, F8), -152.9 (1F, t, $^3J_{FF}$ = 20 Hz, F10), -140.0 (2F, m, F9), -79.3 (9F, s, OTf); **^{31}P NMR** (202.46 MHz, CD_3CN, 300 K, in ppm): δ = -62.3 (1P, t(br), $^1J_{PP}$ = 91 Hz, P_A), -41.9 (2P, dt, $^1J_{PP}$ = 103 Hz, $^4J_{PF}$ = 45 Hz, P_X); **elemental analysis**: calcd. for $C_{43}H_{61}F_{14}N_6O_9P_3S_3$: C: 40.96, H: 4.88, N: 6.66, S: 7.63; found: C: 40.86, H: 4.783, N: 6.40, S: 7.656.

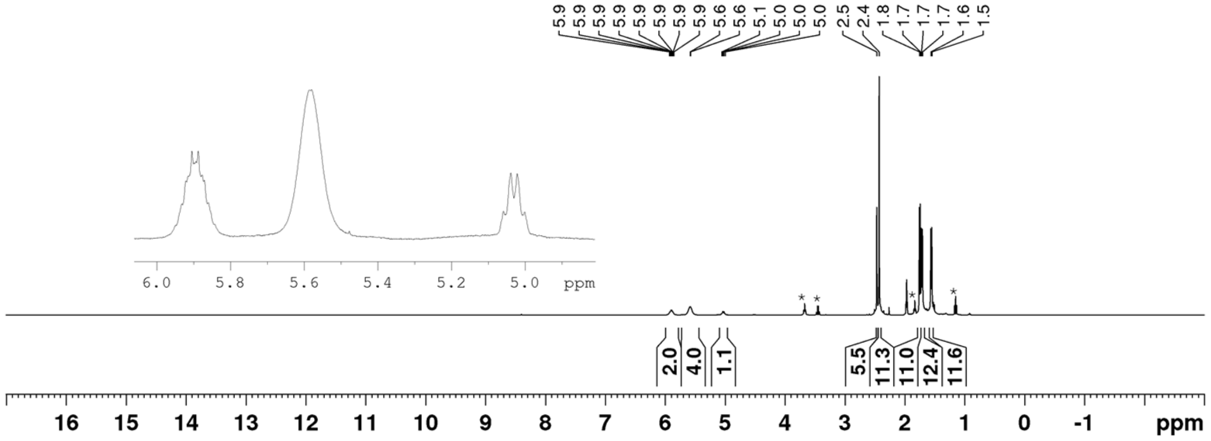

Figure S50. ^{1}H NMR spectrum of **6h**[OTf]$_3$ (CD_3CN, 300 K). Asterisks mark resonances of residual THF and Et_2O solvent impurities.

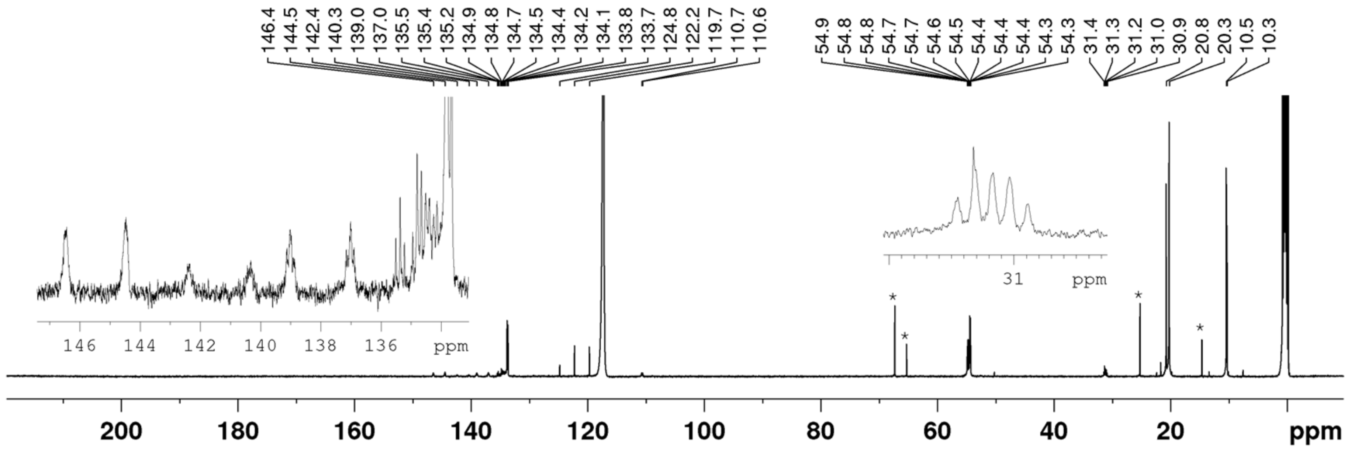

Figure S51. $^{13}C\{^{1}H\}$ NMR spectrum of **6h**[OTf]$_3$ (CD_3CN, 300 K).

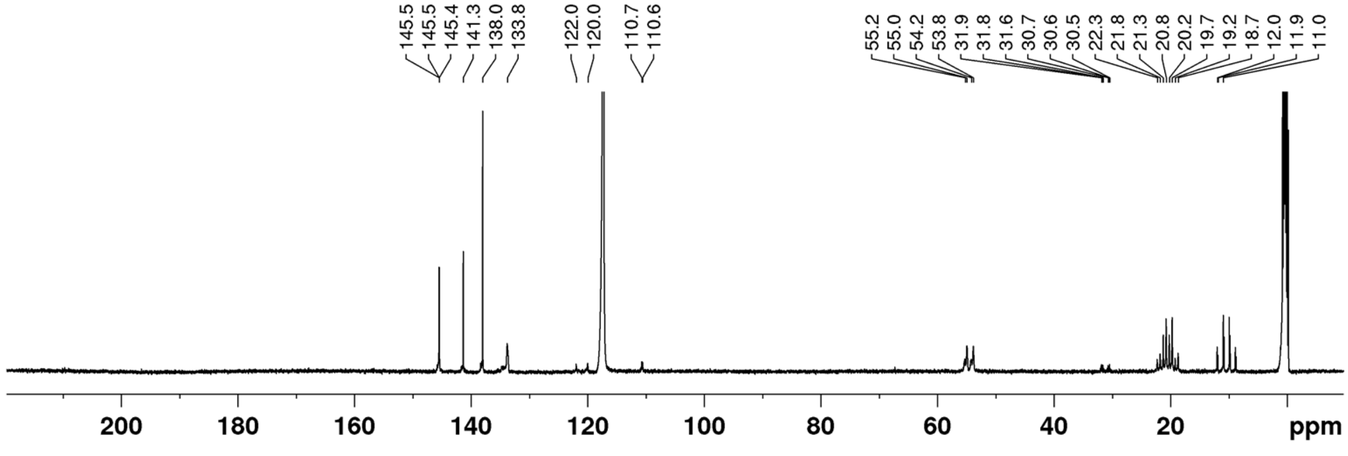

Figure S52. $^{13}C\{^{19}F\}$ NMR spectrum of **6h**[OTf]$_3$ (CD_3CN, 300 K).

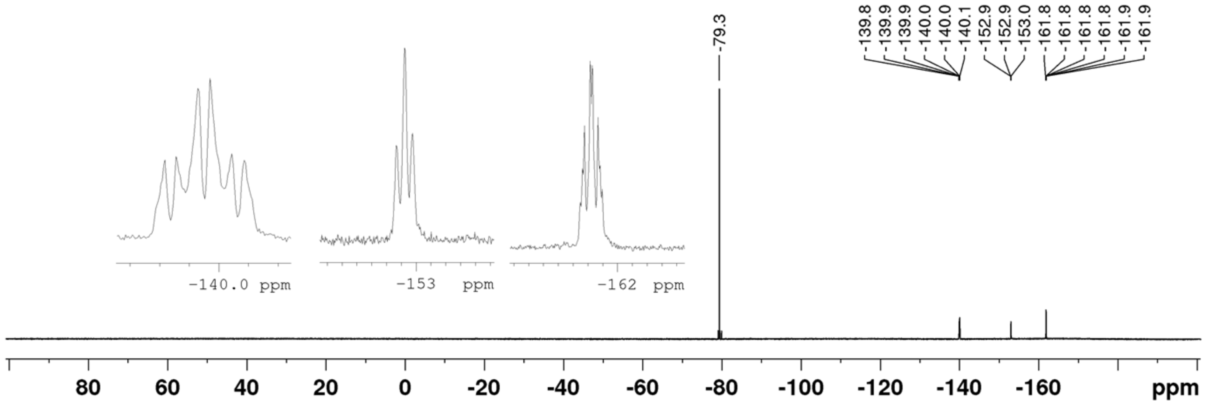

Figure S53. ^{19}F NMR spectrum of **6h**[OTf]$_3$ (CD_3CN, 300 K). Asterisks mark resonances of residual THF and Et_2O solvent impurities.

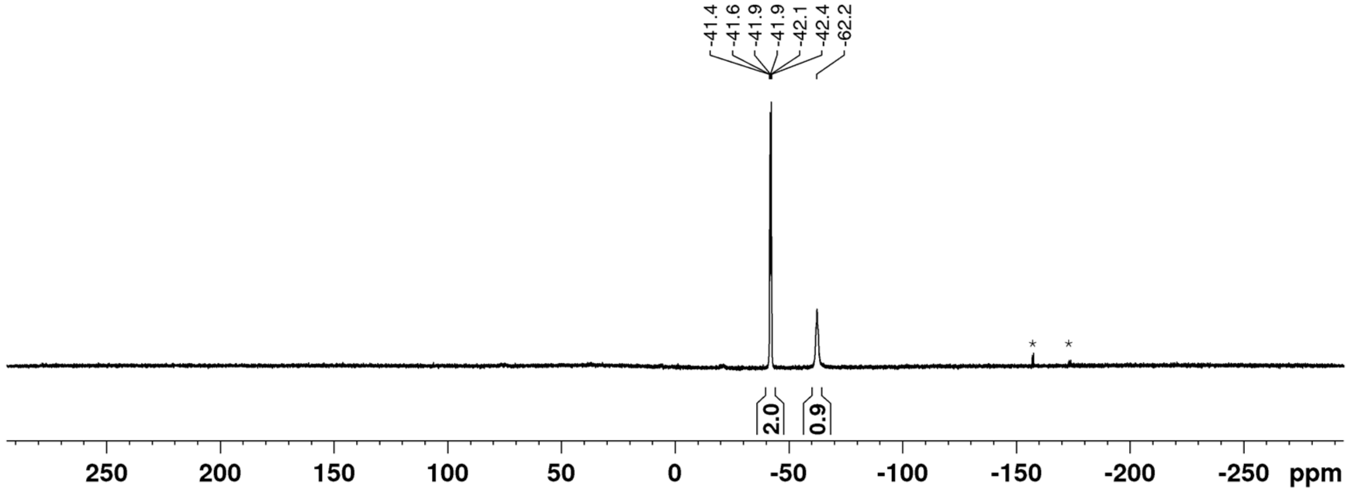

Figure S54. ^{31}P NMR spectrum of **6h**[OTf]$_3$ (CD_3CN, 300 K).

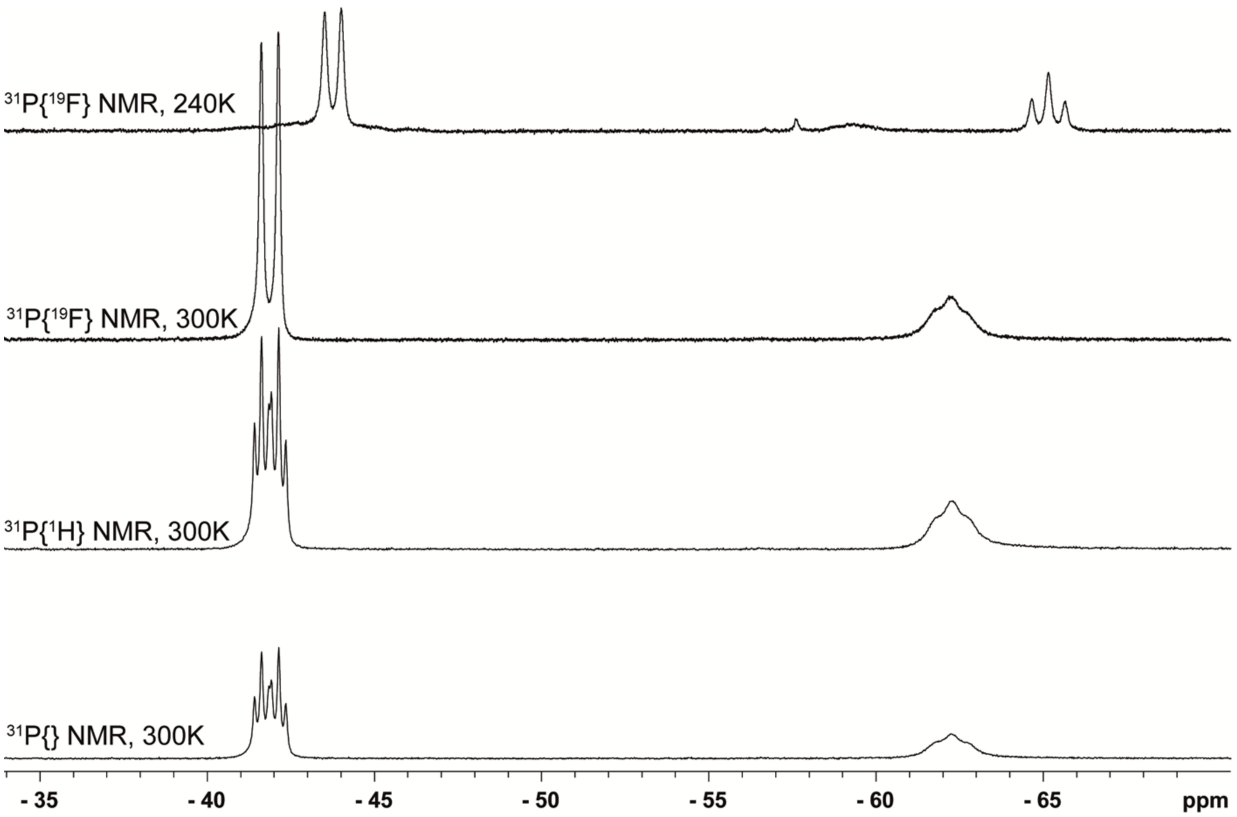

Figure S55. ^{31}P, $^{31}P\{^{1}H\}$, and $^{31}P\{^{19}F\}$ NMR spectra of **6h**[OTf]$_3$ (CD_3CN, 240 K or 300 K).

S2.16 Preparation of [*syn*/*anti*-((L_C)P=C(H)C_6F_5)$_2$][OTf] (*syn*/*anti*-(2h)$_2$[OTf]$_2$)

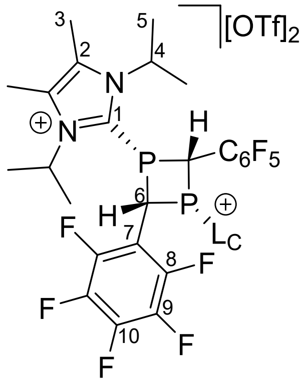

Solid **2h**[OTf] (100 mg, 0.19 mmol, 1.0 equiv.) was suspended in THF (2 ml) and stirred for 16 hours at room temperature. The solid portion was filtered off, washed with THF (2x3 ml) and dried *in vacuo* to afford a colorless solid, of which an analytical sample was dissolved in CD_3CN and subjected to multinuclear NMR analysis at varying temperatures. Fractional crystallization of the solid from slow diffusion of Et_2O into saturated CH_3CN solution for several times afforded an analytically pure fraction of *syn*-(**2h**)$_2$[OTf]$_2$ as an air- and moisture-sensitive colorless solid.

Note: The ^{13}C NMR resonance of C1 was not assigned.

Yield: 45.5 mg (46%); **m.p.**: 212-214 °C (decomp.); **Raman** (100 mW, 100 scans, 298 K, in cm^{-1}): sample showed strong fluorescence; **IR** (ATR, 298 K, , in cm^{-1}): 3002 (vw), 2361 (vw), 1656 (vw), 1611 (vw), 1520 (m), 1505 (s), 1462 (vw), 1399 (w), 1380 (w), 1357 (vw), 1308 (vw), 1262 (vs), 1222 (m), 1137 (s), 1087 (vw), 1031 (vs), 1006 (m), 966 (s), 903 (vw), 789 (vw), 753 (w), 687 (vw), 635 (vs), 600 (w), 571 (w), 563 (w), 543 (vw), 517 (m), 466 (vw), 444 (vw), 408 (w); **^{1}H NMR** (400.13 MHz, CD_3CN, 300 K, in ppm): δ = 1.53 (24H, d, ^{3}J(HH) = 6.8 Hz, H5), 2.40 (12H, s, H3), 4.71 (4H, s(br), H4), 5.54 (2H, t, ^{2}J(HP) = 4.6 Hz, H6); **^{13}C{^{1}H} NMR** (100.61 MHz, CD_3CN, 300 K, in ppm): δ = 10.4 (4C, s, C3), 20.5 (8C, s, C5), 31.7 (2C, t, ^{1}J(CP) = 15 Hz, C6), 53.8 (4C, s, C4), 109.9 (2C, m, C7), 121.1 (2C, q, ^{1}J(CF) = 321 Hz, OTf), 133.2 (4C, s, C2), 138.2 (4C, m, C9), 141.5 (2C, m, C10), 145.7 (4C, m, C8); **^{19}F NMR** (376.5 MHz, CD_3CN, 300 K, in ppm): δ = −163.6 (4F, m, F9), −151.2 (2F, t, ^{4}J(FF) = 20 Hz, F10), −138.7 (4F, dt, ^{4}J(FP) = 47 Hz, ^{4}J(FF) = 18 Hz, F8), −79.3 (3F, s, OTf); **^{31}P NMR** (161.98 MHz, CD_3CN, 300 K, in ppm): δ = −10.9 (2P, s(br), P); **elemental analysis**: calcd. for $C_{38}H_{42}F_{16}N_4O_6P_2S_2$: C: 42.23, H: 3.92, N: 5.18, S: 5.93; found: C: 42.07, H: 3.704, N: 5.26, S: 5.945.

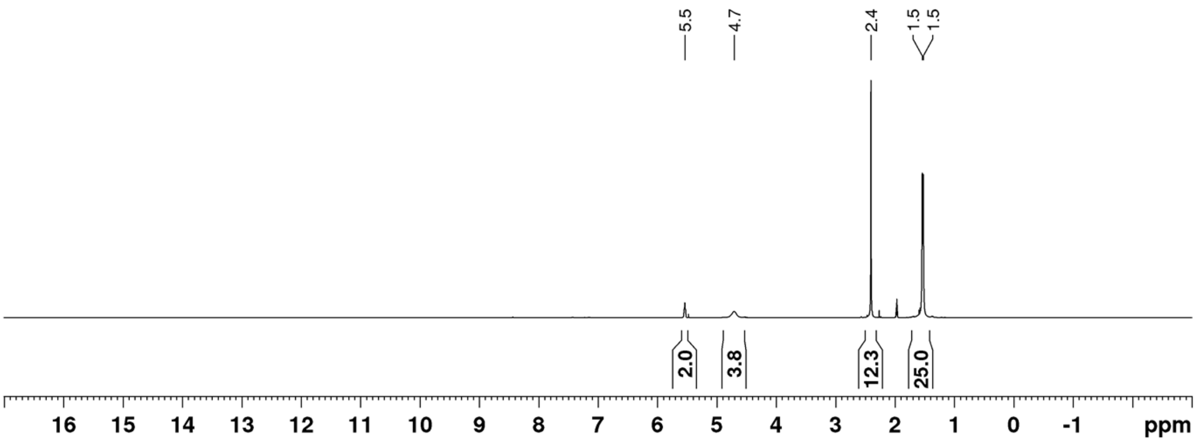

Figure S56. ^{1}H NMR spectrum of *syn*-(**2f**)$_2$[OTf]$_2$ (CD_3CN, 300 K).

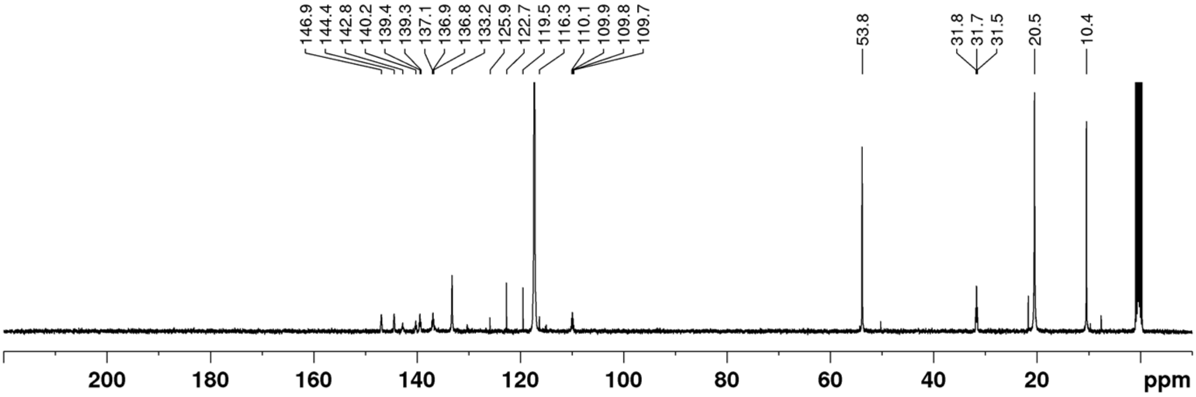

Figure S57. ^{13}C NMR spectrum of *syn*-(**2f**)$_2$[OTf]$_2$ (CD_3CN, 300 K).

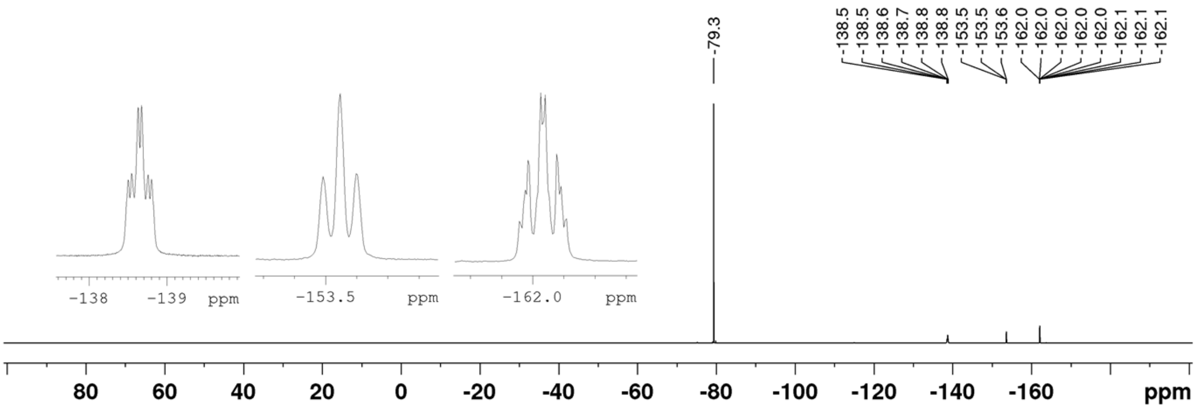

Figure S58. ^{19}F NMR spectrum of *syn*-(**2f**)$_2$[OTf]$_2$ (CD_3CN, 300 K).

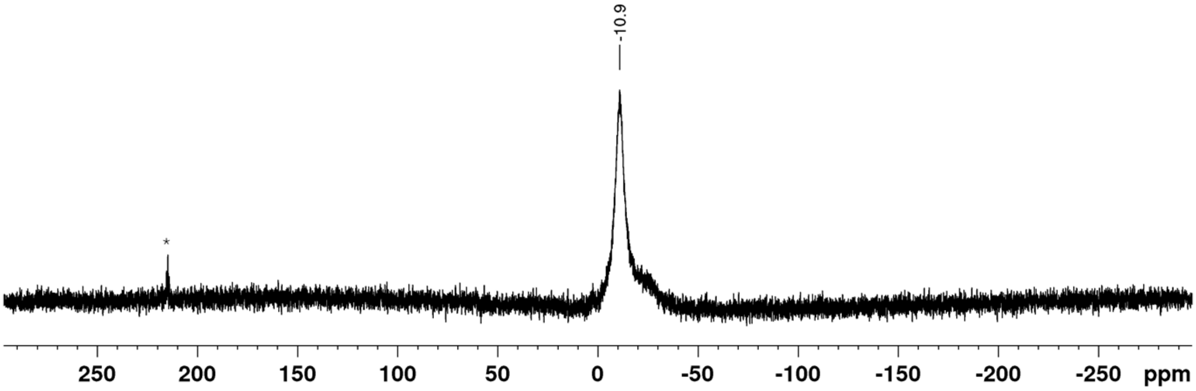

Figure S59. ^{31}P NMR spectrum of *syn*-(**2f**)$_2$[OTf]$_2$ (CD_3CN, 300 K).

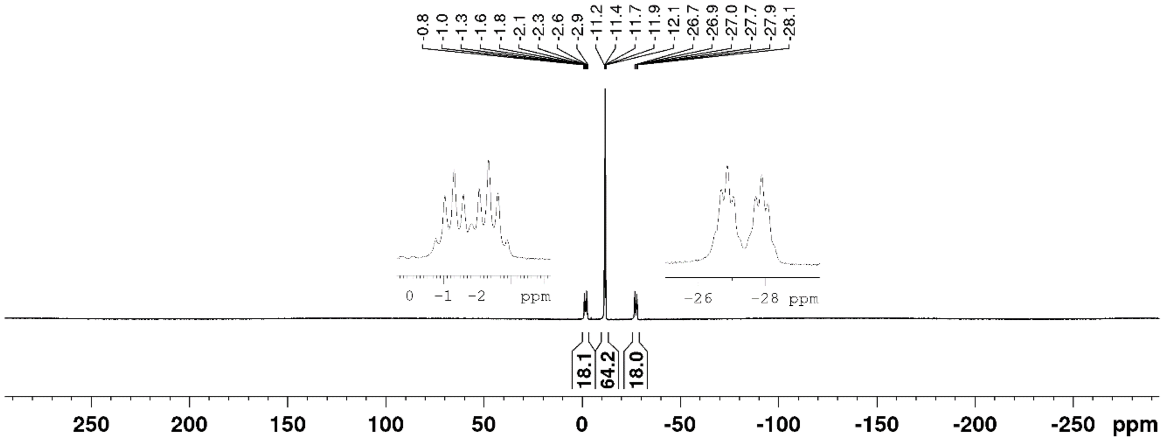

Figure S60. ^{31}P NMR of isolated *syn*-(**2f**)$_2$[OTf]$_2$ (CD_3CN, 240 K).

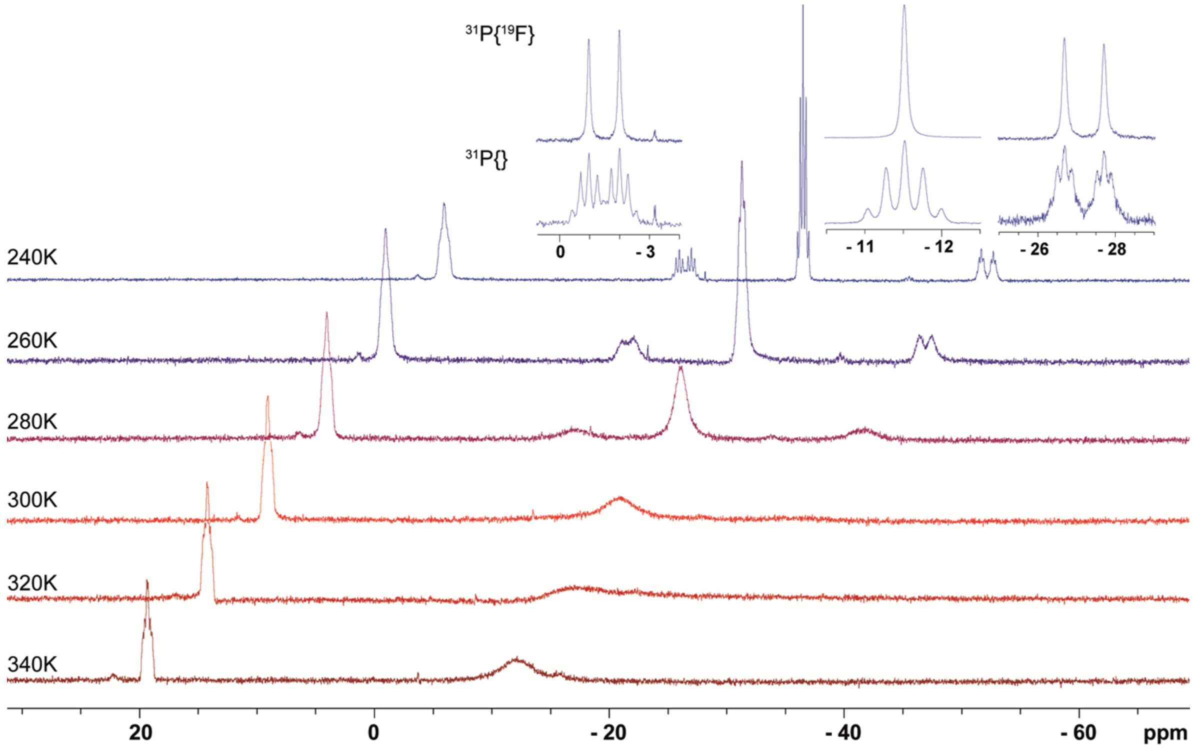

Figure S61. ^{31}P NMR of a mixture of *syn*/*anti*-(**2h**)$_2$[OTf]$_2$ at 240-340 K (CD_3CN).

At 240 K the ^{31}P NMR spectra of the diastereomeric mixture and of isolated *syn*-(**2h**)$_2$[OTf]$_2$ (**Figure S61**) display a major well-resolved *quintet* resonance for *syn*-(**2h**)$_2^{2+}$ [^4J(PF) = 49 Hz] and two new minor resonances of an AX spin system, with similar P–F coupling patterns and constants [$\delta(^{31}P_A)$ = −1.8 ppm, ^4J(PF) = 55 Hz and $\delta(^{31}P_X)$ = −27.3 ppm, ^4J(PF) = 36 Hz]. These resonances are simplified to two *doublets* in the $^{31}P\{^{19}F\}$ NMR spectrum due to scalar P–P coupling [^1J(PP) = 207 Hz], suggesting the presence of a third diastereomer with no C_2 symmetry in a dynamic equilibrium with *syn*-(**2h**)$_2^{2+}$. This is supported by the observation of new resonances in the ^1H NMR spectrum at 240 K, indicating two inequivalent imidazoliumyl-substituents (**Figure S62**).

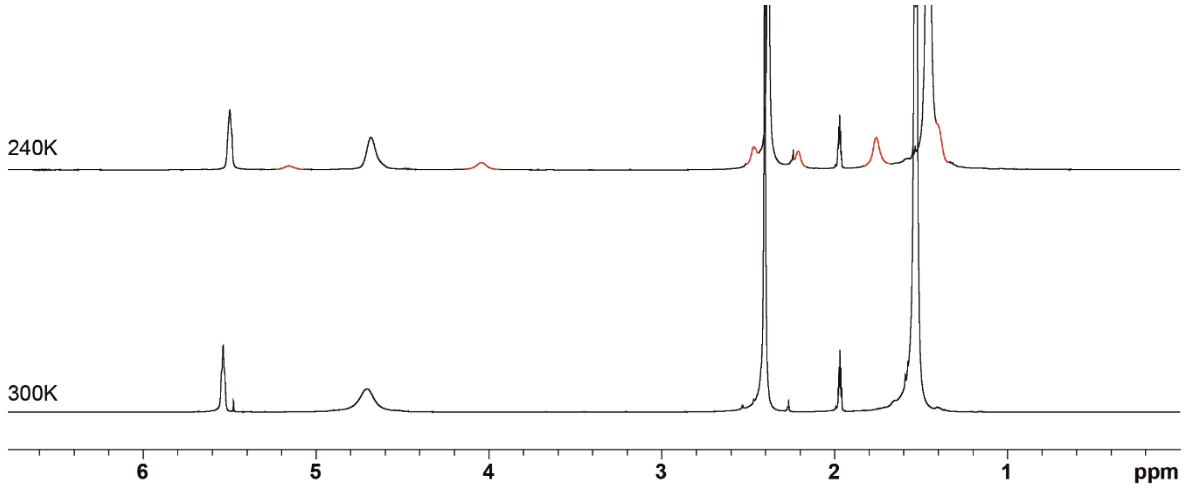

Figure S62. ^{1}H NMR of isolated *syn*-(**2f**)$_2$[OTf]$_2$ at 300 K and 240 K (CD_3CN); resonances of minor isomer at 240 K are marked in red.

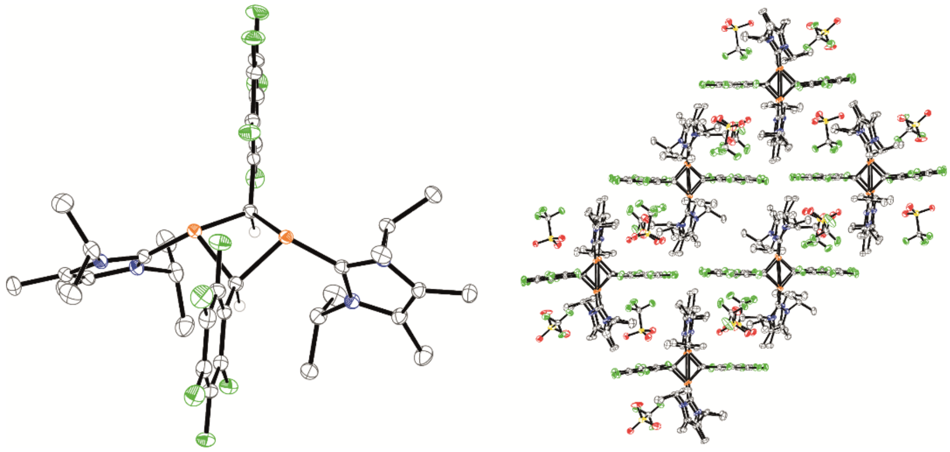

Figure S63. Molecular structure of [*syn*-(**2f**)$_2$]$^{2+}$ in [*syn*-(**2f**)$_2$][OTf]$_2$ and packing order in the crystal structure of [*syn*-(**2f**)$_2$][OTf]$_2$; hydrogen atoms and anions are omitted for clarity, and thermal ellipsoids are displayed at 50% probability.

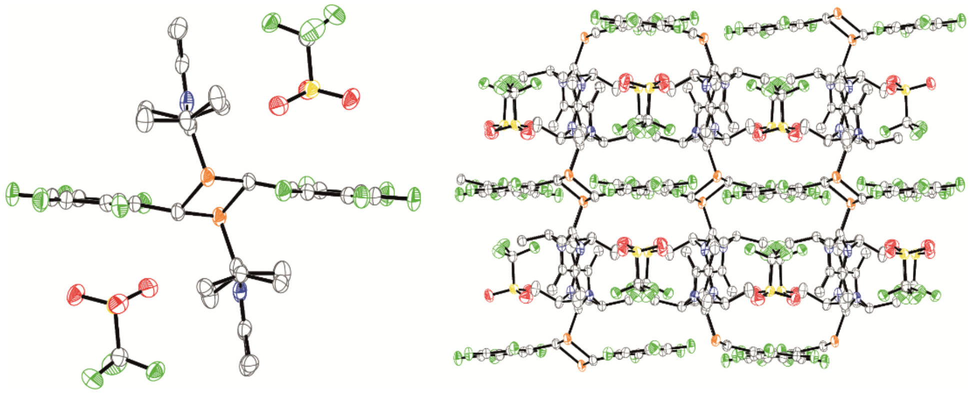

Figure S64. Molecular structure of [*anti*-(**2f**)$_2$]$^{2+}$ in [*anti*-(**2f**)$_2$][OTf]$_2$ and packing order in the crystal structure of [*anti*-**2f**)$_2$][OTf]$_2$; hydrogen atoms and anions are omitted for clarity, and thermal ellipsoids are displayed at 50% probability.

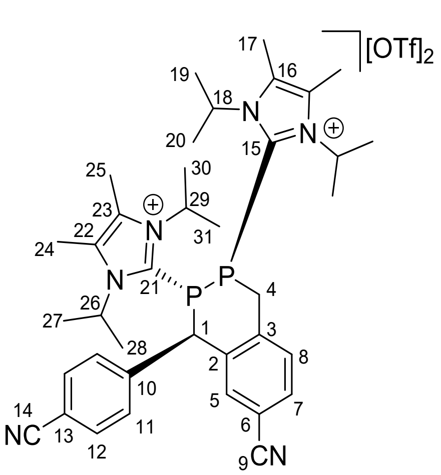

S2.17 Preparation of 2,3-Di(imidazoliumyl)-7-cyano-1-(4-cyanophenyl)-1,2,3,4-tetrahydrobenzo[d][1,2]diphosphinine (7e[OTf]$_2$)

Method A: To a solution of **1c**[OTf] (500 mg, 0.88 mmol, 1.0 equiv.) in CH_3CN (10 ml), 4-cyanobenzaldehyde (576 mg, 4.4 mmol, 5.0 equiv.) was added as a solid while stirring. The resulting yellow to orange-colored solution was stirred for 20 hours at room temperature. Subsequently, all volatiles were removed from the reaction mixture *in vacuo*, and the resulting yellow-to-orange residue was

stirred over a mixture of Et_2O (10 ml) and toluene (5 ml) for three days, after which the supernatant was replaced by fresh Et_2O (10 ml). The suspension was then stirred until a free flowing solid was obtained, which was filtered and dried *in vacuo* to give the crude product as an air- and moisture sensitive off-white powder (90% purity, 354 mg).

Method B: To a solution of **1c**[OTf] (250 mg, 0.44 mmol, 1.0 equiv.) in CH_3CN (5 ml), 4-cyanobenzaldehyde (288 mg, 2.2 mmol, 5.0 equiv.) was added as a solid while stirring. The resulting yellow to orange-colored solution was stirred for seven days at room temperature. Subsequently, all volatiles were removed from the reaction mixture *in vacuo*, and the resulting yellow-to-orange residue was stirred over THF (3 ml), filtered, washed with minimal amounts of THF (2x2 ml), and dried *in vacuo* to obtain an air- and moisture-sensitive off-white solid (yield = 112 mg). The solid was analyzed by means of multinuclear NMR analysis (**Figure S67**) and recrystallized twice from vapor diffusion of Et_2O into saturated CH_3CN solutions at room temperature to obtain the analytically pure product as colorless crystals. Crystals suitable for X-ray diffraction analysis of **7e**[OTf]$_2$ and *syn*-(**2e**)$_2$[OTf]$_2$ (**Figure S65**) were obtained by the same method.

Note: The two L_C substituents exhibited resonances with only slightly different chemical shifts causing severe overlapping in the ^{1}H and ^{13}C NMR spectra, which prevented unambiguous assignment of all atoms.

Yield: 63.0 mg (30%, Method B); **m.p.**: 257-259 °C; **Raman** (100 mW, 100 scans, 298 K, in cm^{-1}): 3071 (8), 2980 (16), 2950 (25), 2898 (11), 2795 (6), 2745 (6), 2690 (6), 2506 (7), 2445 (8), 2400 (8), 2378 (8), 2309 (8), 2284 (9), 2262 (10), 2229 (100), 2179 (10), 2158 (9), 2114 (11), 2074 (10), 2061 (11), 1690 (16), 1675 (16), 1605 (78), 1564 (20), 1448 (26), 1407 (31), 1386 (24), 1359 (32), 1278 (53), 1234 (29), 1198 (27), 1180 (45), 1150 (26), 1088 (20), 1032 (33), 983 (18), 950 (21), 883 (23), 849 (18), 832 (19), 792 (24), 772 (25), 755 (25), 745 (22), 722 (26), 699 (23); **IR** (ATR, 298 K, in cm^{-1}): 3091 (vw), 3044 (vw), 2986 (vw), 2948 (vw), 2227 (vw), 1604 (vw), 1503 (vw), 1490 (vw), 1462 (vw), 1397 (w), 1378 (vw), 1358 (vw), 1329 (vw), 1258 (vs), 1223 (m), 1150 (m), 1140 (m), 1116 (w), 1086 (vw), 1029 (s), 982 (vw), 947 (vw), 902 (vw), 859 (w), 835 (vw), 789 (vw), 771 (vw), 753 (w), 698 (vw), 687 (vw), 636 (vs), 572 (w), 556 (w), 516 (m), 491 (vw), 470 (vw), 452 (vw), 428 (w), 410 (vw); **^{1}H NMR** (500.13 MHz, CD_2Cl_2, 300 K, in ppm): δ = 1.28-1.60 (18H, m, H19 and H27 and H28 and H30 and H31), 1.77 (3H, d(br), $^3J_{HH}$ = 5.9 Hz, H20), 2.35 (3H, s(br), H24), 2.37 (6H, s(br), H17), 2.41 (3H, s(br), H25), 3.70 (1H, d, $^2J_{HH}$ = 15.0 Hz, H4$_{eq}$), 4.52 (1H, dd, $^2J_{HP}$ = 27.1 Hz, $^2J_{HH}$ = 15.1 Hz, H4$_{ax}$), 4.89 (2H, s(br), H18), 5.02 (1H, s(br), H26), 5.62 (1H, s(br), H29), 5.89 (1H, s, H1), 7.47 (1H, s, H5), 7.71 (3H, m, H8 and H11), 7.75 (1H, d, $^3J_{HH}$ = 7.6 Hz, H7), 7.84 (2H, d, $^3J_{HH}$ = 7.3 Hz, H12); **^{13}C{^{1}H} NMR** (125.76 MHz, CD_2Cl_2, 300 K, in ppm): δ = 10.8 (3C, s, C17 and C24), 11.0 (1C, s, C25), 20.9-21.8 (8C, s, C19 and C20 and C27 and C28 and C30 and C31), 28.0 (1C, d, ^1J(CP) = 22 Hz, C4), 45.3 (1C, d, ^1J(CP) = 20 Hz, C1), 54.1 (2C, m, C26 and C29), 55.4 (2C, d, ^3J(CP) = 15 Hz, C18), 112.8 (1C, s, C13), 112.9 (1C, s, C6), 117.7 (1C, s, C9), 117.8 (1C, s, C14), 120.7 (1C, q, ^1J(CF) = 321 Hz, OTf), 130.4 (2C, d, ^3J(CP) = 9 Hz, C11), 131.4 (1C, s, C8), 132.1 (1C, s, C5), 132.3 (2C, s(br), C16), 132.8 (1C, s, C7), 133.5 (2C, s(br), C22 and C23), 133.7 (2C, s, C12), 136.3 (1C, m, C21), 137.1 (1C, m, C3), 138.1 (1C, m, C15), 140.3 (1C, s, C2), 141.4 (1C, s(br), C10); **^{19}F NMR** (470.59 MHz, CD_2Cl_2, 300 K, in ppm): δ = −79.2 (6F, s, OTf); **^{31}P{^{1}H} NMR** (202.46 MHz, CD_2Cl_2, 300 K, in ppm): AB spin system centered at $\delta(P_{AB})$ = −51.1 ppm, iteratively fitted: $\delta(^{31}P_A)$ = −52.2 ppm, $\delta(^{31}P_B)$ = −50.4 ppm, ^1J(PP) = −190 Hz; **elemental analysis**: calcd. for $C_{40}H_{50}F_6N_6O_6P_2S_2$: C: 50.52, H: 5.30, N: 8.84, S: 6.74; found: C: 50.43, H: 4.907, N: 8.88, S: 6.781.

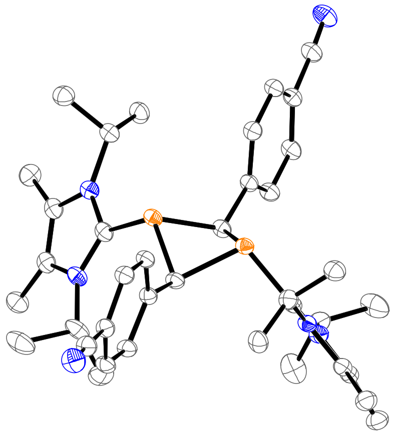

Figure S65. Molecular structures of *syn*-(**2e**)$_2^{2+}$ in *syn*-(**2e**)$_2$[OTf]$_2$; hydrogen atoms, anions, and solvent molecules are omitted for clarity, and thermal ellipsoids are displayed at 50% probability.

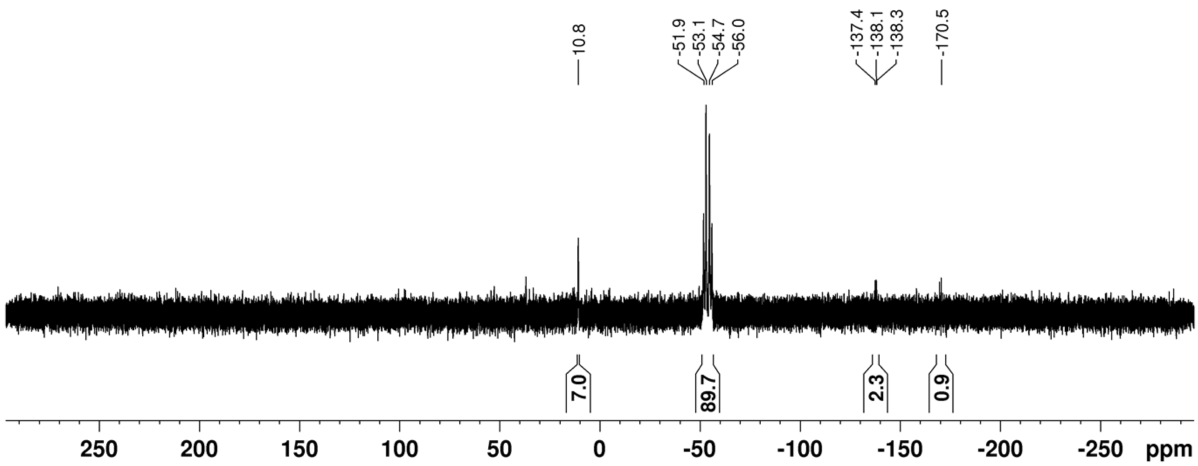

Figure S66. ^{31}P NMR spectrum of the crude solid obtained during work-up of **6e**[OTf]$_2$ using method A (CD_3CN, 300 K); the major resonances are assigned to **7e**[OTf]$_2$ [$\delta(^{31}P_{AB}) = -54.0$, $^1J(P_AP_B) = -194$ Hz], minor resonance at $\delta(^{31}P) = 10.7$ ppm is assigned to *anti*-(**2e**)$_2$[OTf]$_2$.

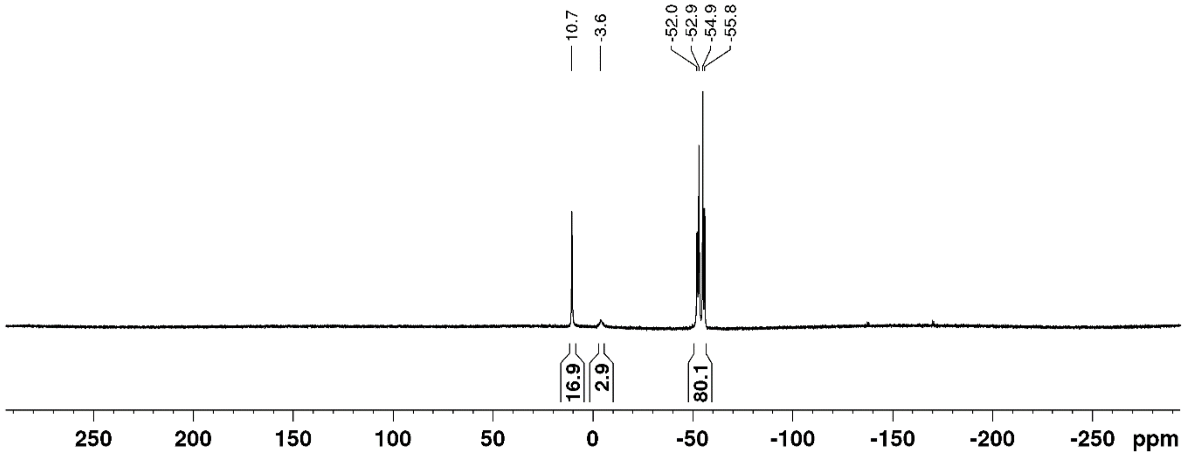

Figure S67. ^{31}P NMR spectrum of the crude solid obtained during work-up of **7e**[OTf]$_2$ using method B (CD_3CN, 300 K); the major resonances are assigned to **7e**[OTf]$_2$ [$\delta(^{31}P_{AB}) = -54.0$, $^1J(P_AP_B) = -194$ Hz], minor resonances at $\delta(^{31}P) = -3.6$ ppm and $\delta(^{31}P) = 10.7$ ppm are assigned to *syn*-(**2e**)$_2$[OTf]$_2$ and *anti*-(**2e**)$_2$[OTf]$_2$, respectively.

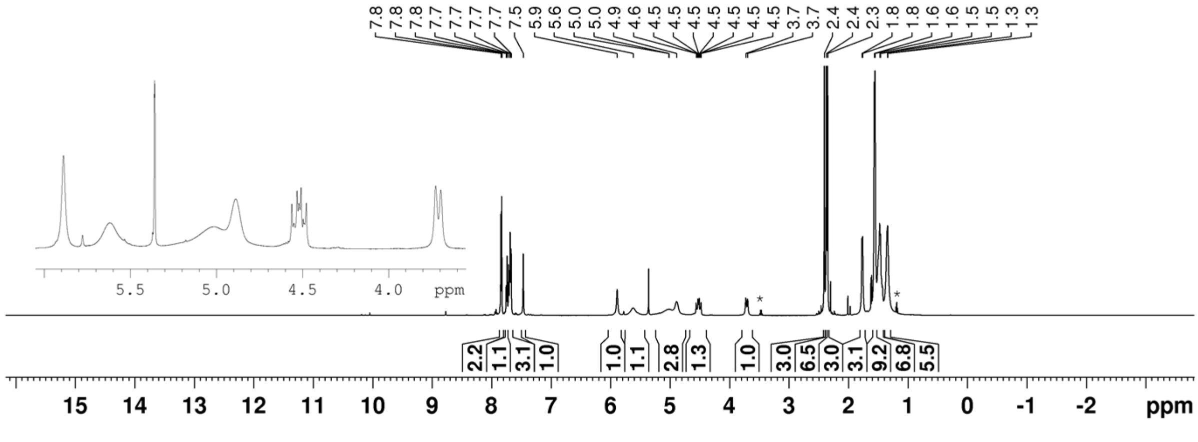

Figure S68. ^{1}H NMR spectrum of **7e**[OTf]$_2$ (CD_2Cl_2, 300 K). Asterisks mark trace amounts of Et_2O.

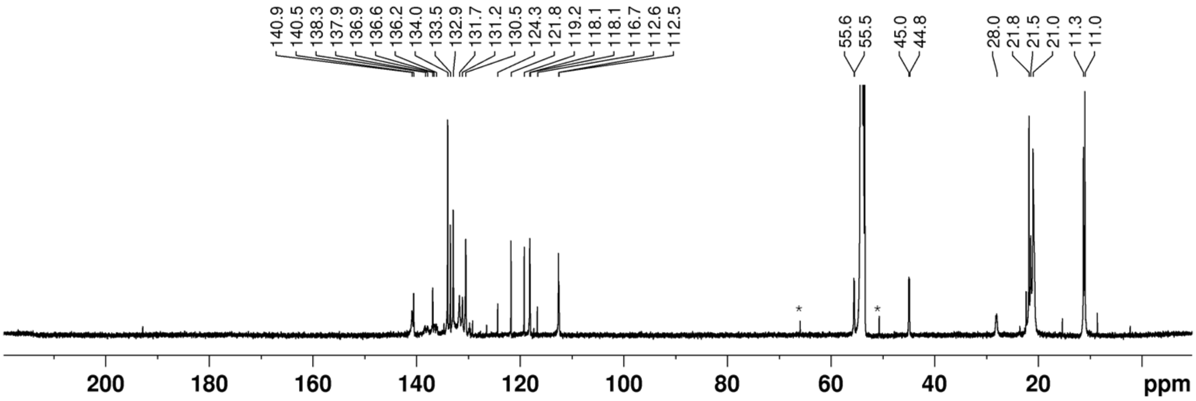

Figure S69. ^{13}C NMR spectrum of **7e**[OTf]$_2$ (CD_2Cl_2, 300 K). Asterisks mark trace amounts of Et_2O.

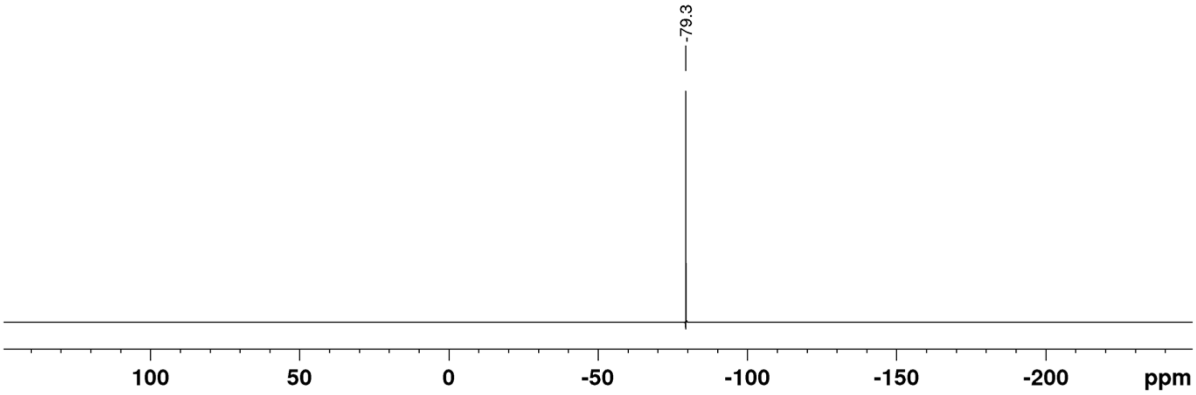

Figure S70. ^{19}F NMR spectrum of **7e**[OTf]$_2$ (CD_2Cl_2, 300 K).

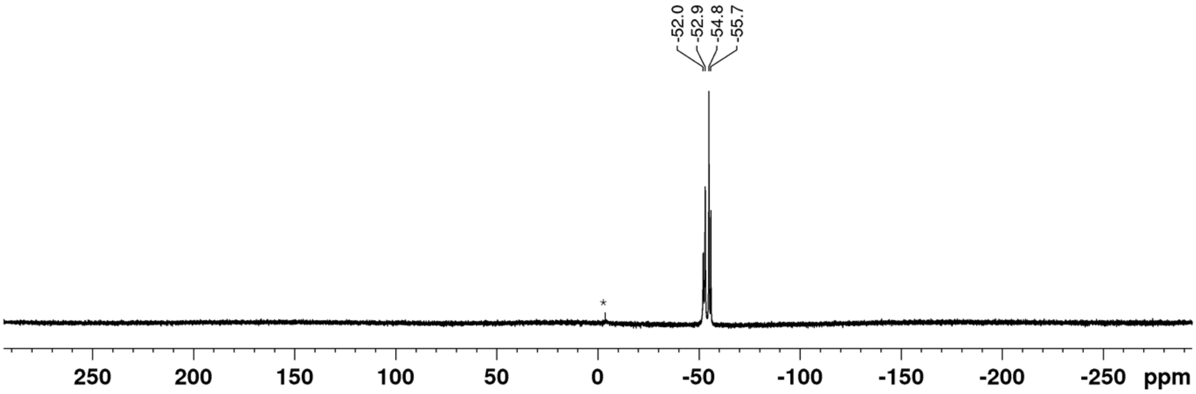

Figure S71. ^{31}P NMR spectrum of **7e**[OTf]$_2$ (CD_2Cl_2, 300 K).

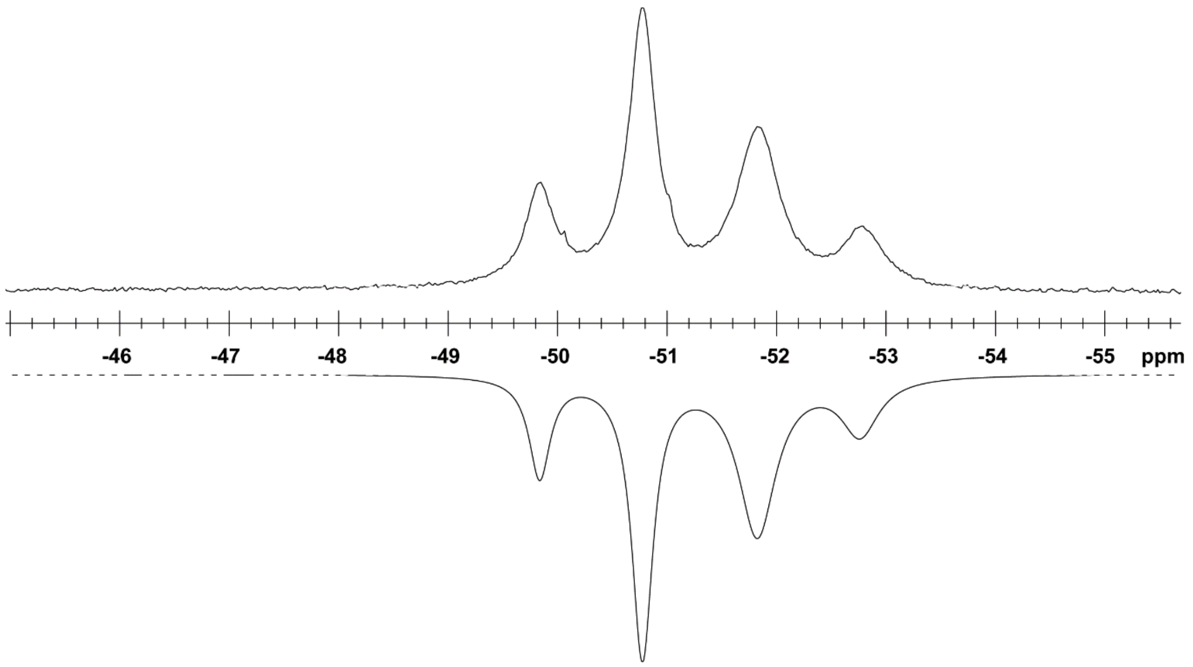

Figure S72. Measured (upwards) and simulated (downwards) $^{31}P\{^{1}H\}$ NMR spectrum of **7e**[OTf]$_2$ (CD_2Cl_2, 300 K).

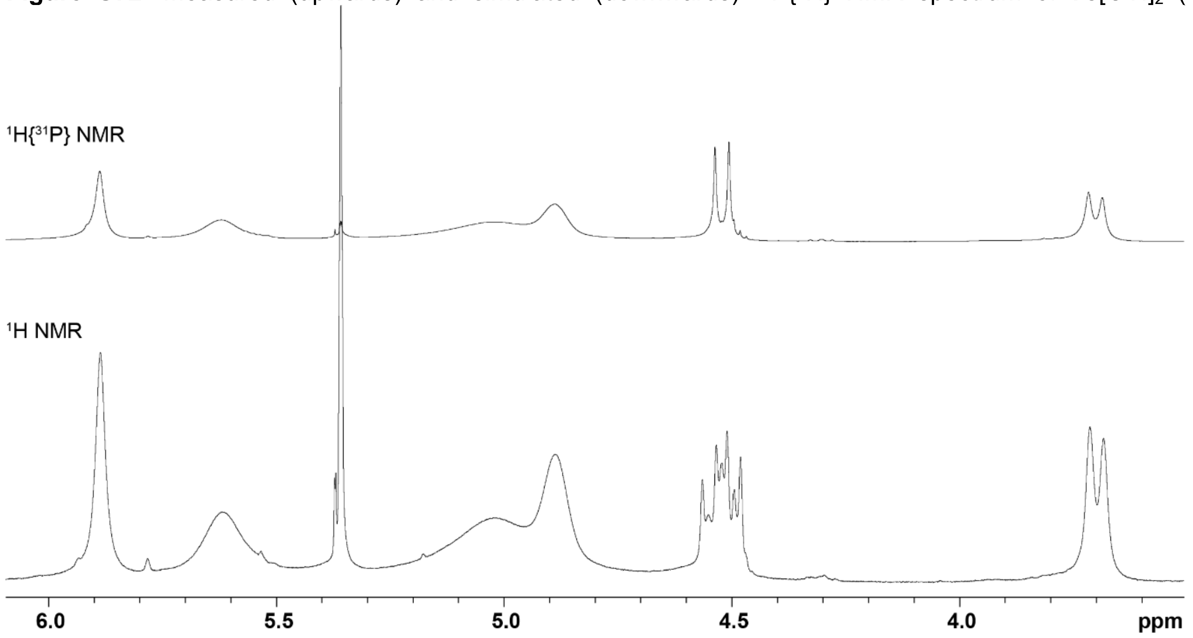

Figure S73. ^{1}H and $^{1}H\{^{31}P\}$ NMR spectra of **7e**[OTf]$_2$ (CD_2Cl_2, 300 K).

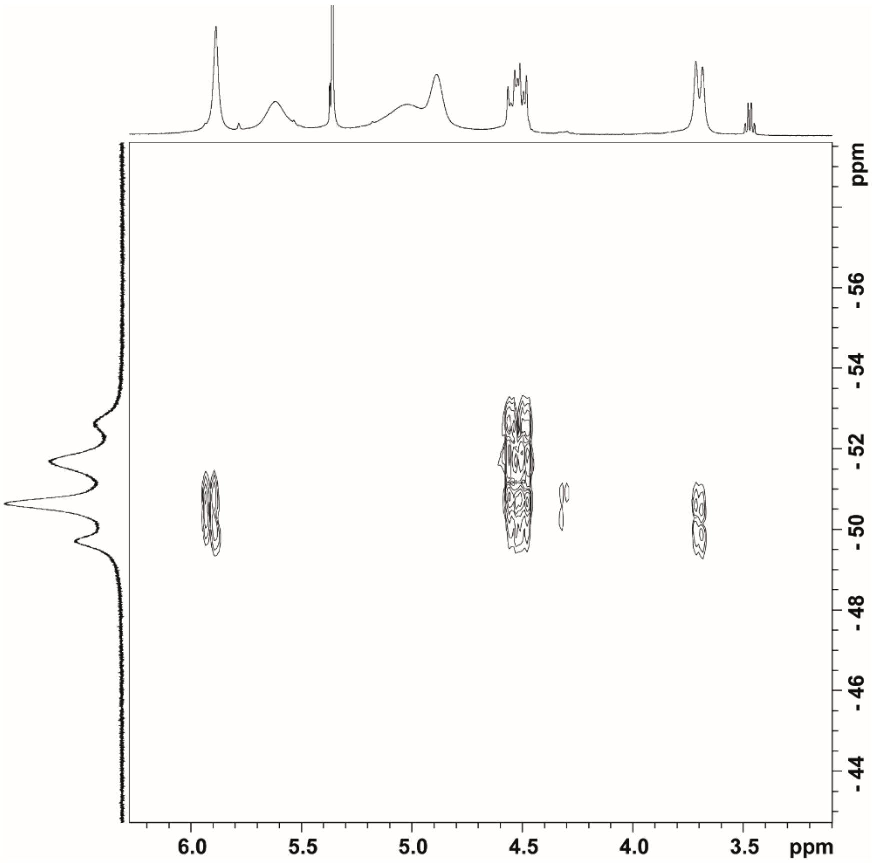

Figure S74. $^{1}H\{^{31}P\}$ HMBC NMR spectrum of **7e**[OTf]$_2$ (CD_2Cl_2, 300 K).

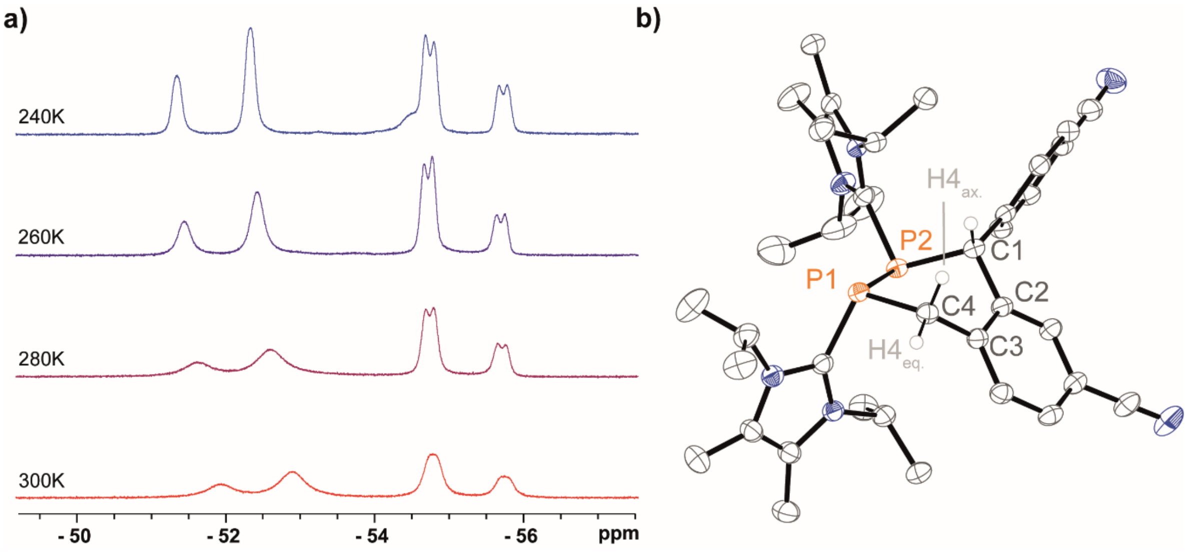

Figure S75. a) ^{31}P NMR spectra of **7e**[OTf]$_2$ at varying temperatures (CD_3CN, 240-300 K); b) molecular structure of **7e**$^{2+}$ in **7e**[OTf]$_2$ • CH_3CN; selected hydrogen atoms, solvent molecules and anions are omitted for clarity, and thermal ellipsoids are displayed at 50% probability; selected bond lengths (Å) and angles (°): P1–P2 2.2073(6), P1–C4 1.8636(16), P2–C1 1.8966(17), P1–C4–C3 118.22(12), P2–C1–C2 105.00(11), C1–P1–P2–C4 –25.17(8).

Upon stepwise cooling of a solution of **7e**[OTf]$_2$ in CD_3CN to 240 K a PH coupling [^{2}J(PH) = 22 Hz] of the A part to one of the H4 protons was observed, supported by $^{1}H\{^{31}P\}$ and $^{1}H\{^{31}P\}$ HMBC NMR experiments (**Figure S73-S75**). Further cooling of a CD_2Cl_2 solution of **7e**[OTf]$_2$ to 190 K did not further resolve the spin system. Based on precedents in cyclic[7] and acyclic[8] phosphanes as well as phospholenes[9], where high values for the geminal ^{2}J(PH) are found for protons with a *syn*-configuration to the lone pair at phosphorus[10], the coupling was assigned the to the P1–H4$_{ax.}$ interaction.

S2.18 Preparation of 2,3-Di(imidazoliumyl)-7-nitro-1-(4-nitrophenyl)-1,2,3,4-tetrahydrobenzo[d][1,2]diphosphinine (7f[OTf]₂)

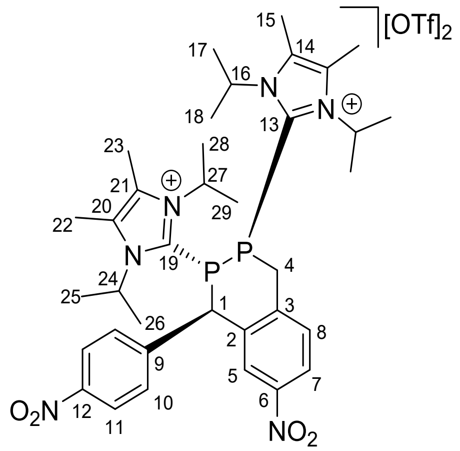

To a solution of **1c**[OTf] (250 mg, 0.44 mmol, 1.0 equiv.) in CH_3CN (10 ml), 4-nitrobenzaldehyde (665 mg, 2.2 mmol, 5.0 equiv.) was added as a solid while stirring. The resulting dark yellow to orange-colored solution was stirred for four hours at room temperature. Subsequently, all volatiles were removed from the reaction mixture *in vacuo*, and the resulting yellow-to-orange residue was stirred over a mixture of Et_2O (10 ml) and toluene (5 ml) for 16 hours, after which the supernatant was replaced by fresh Et_2O (10 ml). The supernatant was removed by decantation after another 16 hours at room temperature and to the resulting solid residue THF (2 ml) was added giving a red oil with an orange-colored supernatant. Addition of more THF (5 ml) eventually lead to a clear solution, which was loaded onto a silica plug (3 ml in 10 ml syringe). The plug was further washed with 40 ml of THF giving an orange-colored fraction, which was evaporated to dryness *in vacuo*, dissolved in CH_3CN and subjected to multinuclear NMR analysis, indicating the presence of minimal amounts of the product. Another fraction was obtained by rinsing the plug with 20 ml of CH_3CN. Evaporation of all volatiles from this fraction gave an orange-colored residue that was precipitated as an amorphous solid from slow diffusion of Et_2O into a saturated CH_3CN solution at –30°C to give a crude product as an air- and moisture sensitive yellow powder (95% purity, 127 mg). An analytically pure sample was obtained after another recrystallization step from CH_3CN/Et_2O at –30°C affording yellow crystals (30 mg), which were also suitable for single crystal X-ray diffraction analysis.
Note: The two L_C substituents exhibited resonances with only slightly different chemical shifts causing severe overlapping in the ^1H and ^{13}C NMR spectra, which prevented unambiguous assignment of all atoms.

Yield: 30.0 mg (7%); **m.p.**: 189-191 °C; **Raman** (400 mW, 50 scans, 298 K, in cm^{-1}): 3082 (9), 2979 (26), 2946 (41), 1611 (18), 1595 (31), 1527 (6), 1449 (10), 1403 (15), 1349 (100), 1273 (39), 1232 (6), 1193 (9), 1150 (10), 1109 (13), 1033 (21), 883 (6), 790 (6), 754 (9), 715 (6), 695 (6), 626 (7), 574 (5), 498 (9), 348 (6), 312 (8), 273 (5), 193 (5); **IR** (ATR, 298 K, in cm^{-1}): 2984 (vw), 2941 (vw), 2889 (vw), 1608 (vw), 1524 (w), 1492 (vw), 1462 (vw), 1395 (w), 1377 (vw), 1347 (m), 1258 (s), 1222 (m), 1147 (m), 1112 (w), 1083 (w), 1030 (s), 981 (vw), 938 (vw), 904 (vw), 879 (vw), 861 (w), 840 (w), 823 (vw), 810 (vw), 788 (vw), 752 (w), 704 (w), 680 (vw), 636 (vs), 572 (w), 542 (vw), 517 (m), 466 (w), 441 (w), 409 (vw); **^{1}H NMR** (500.13 MHz, CD_3CN, 300 K, in ppm): δ = 1.29 (6H, s(br), H18), 1.35 (3H, d, $^3J_{HH}$ = 6.2 Hz, H28), 1.40 (3H, s(br), H29), 1.53 (6H, d, $^3J_{HH}$ = 6.9 Hz, H17), 1.57 (3H, d, $^3J_{HH}$ = 6.8 Hz, H25), 1.78 (3H, d, $^3J_{HH}$ = 6.5 Hz, H26), 2.30 (3H, s(br), H22), 2.37 (6H, s(br), H15), 2.39 (3H, s(br), H23), 3.84 (1H, dd, $^2J_{HH}$ = 15.6 Hz, $^2J_{HP}$ = 2.4 Hz, H4$_{eq}$), 4.19 (1H, m, H4$_{ax}$), 4.85 (3H, s(br), H16 and H24), 5.53 (1H, s(br), H27), 5.63 (1H, s, H1), 7.78 (2H, m, H10), 7.81 (1H, d, $^3J_{HH}$ = 8.5 Hz, H8), 7.89 (1H, s, H5), 8.30 (1H, dd, $^3J_{HH}$ = 8.4 Hz, $^4J_{HH}$ = 2.2 Hz, H7), 8.36 (2H, m, H11); **^{13}C{^{1}H} NMR** (125.76 MHz, CD_3CN, 300 K, in ppm): δ = 10.3 (4C, s, C15 and C22 and C23), 20.0-21.2 (8C, s, C17 and C18 and C25 and C26 and C28 and C29), 27.4 (1C, d, $^1J_{CP}$ = 25 Hz, C4), 45.4 (1C, d, $^1J_{CP}$ = 20 Hz, C1), 54.1 (2C, d, $^3J_{CP}$ = 34 Hz, C16), 54.2 (1C, d, $^3J_{CP}$ = 23 Hz, C27), 55.1 (1C, d, $^3J_{CP}$ = 16 Hz, C24), 121.1 (1C, q, $^1J_{CF}$ = 321 Hz, OTf), 123.3 (1C, s(br), C8), 123.3 (1C, s, C5), 124.2 (1C, s, C7), 125.1 (2C, s, C11), 131.0 (2C, d, $^3J_{CP}$ = 10 Hz, C10), 132.6 (2C, s(br), C14), 133.5 (1C, s(br), C21), 133.6 (1C, s(br), C20), 136.0 (1C, dd, $^1J_{CP}$ = 64 Hz, $^2J_{CP}$ = 26 Hz, C19), 137.0 (1C, d, $^2J_{CP}$ = 10 Hz, C2), 137.5 (1C, dd(br), $^1J_{CP}$ = 69 Hz, $^2J_{CP}$ = 21 Hz, C13), 142.5 (1C, s(br), C3), 142.7 (1C, d, $^2J_{CP}$ = 14 Hz, C9), 148.0 (1C, s, C6), 148.1 (1C, s(br), C12); **^{19}F NMR** (470.59 MHz, CD_3CN, 300 K, in ppm): δ = -79.2 (6F, s, OTf); **^{31}P{^{1}H} NMR** (202.46 MHz, CD_3CN, 300 K, in ppm): AB spin system centered at $\delta(P_{AB})$ = −53.2 ppm, iteratively fitted: $\delta(^{31}P_A)$ = −55.8 ppm, $\delta(^{31}P_B)$ = −52.2 ppm, $^1J(PP)$ = −193 Hz; **elemental analysis**: calcd. for $C_{38}H_{50}F_6N_6O_{10}P_2S_2$: C: 46.06, H: 5.09, N: 8.48, S: 6.47; found: C: 45.80, H: 4.847, N: 8.28, S: 6.455.

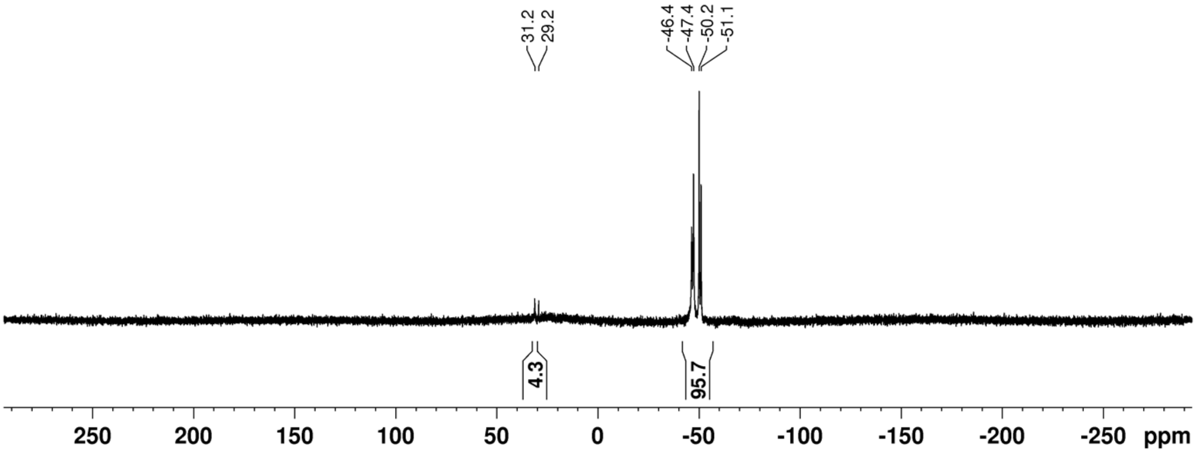

Figure S76. ^{31}P NMR spectrum of the crude solid obtained during work-up of **7f**[OTf]$_2$ (CD_3CN, 300 K); the major resonances are assigned to **7f**[OTf]$_2$ [$\delta(^{31}P_{AB})$ = −48.9, $^{1}J(P_AP_B)$= −192 Hz], minor resonances at $\delta(^{31}P)$ = 29.2 ppm and $\delta(^{31}P)$ = 31.2 ppm were not assigned.

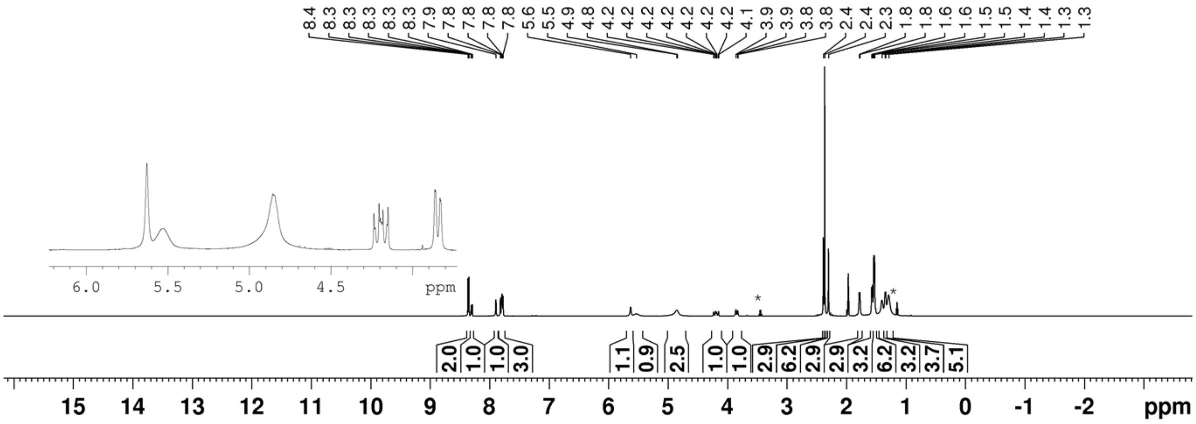

Figure S77. ^{1}H NMR spectrum of **7f**[OTf]$_2$ (CD_3CN, 300 K). Asterisks mark trace amounts of Et_2O.

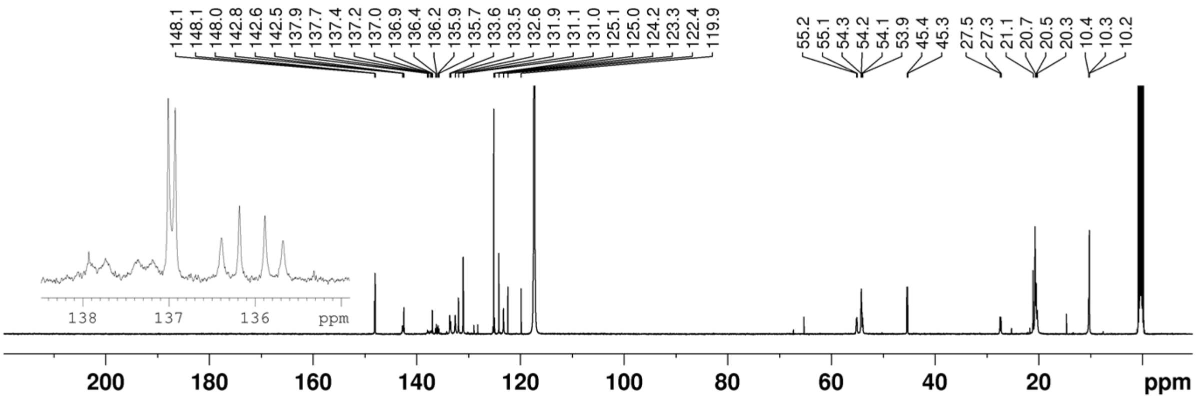

Figure S78. ^{13}C NMR spectrum of **7f**[OTf]$_2$ (CD_3CN, 300 K).

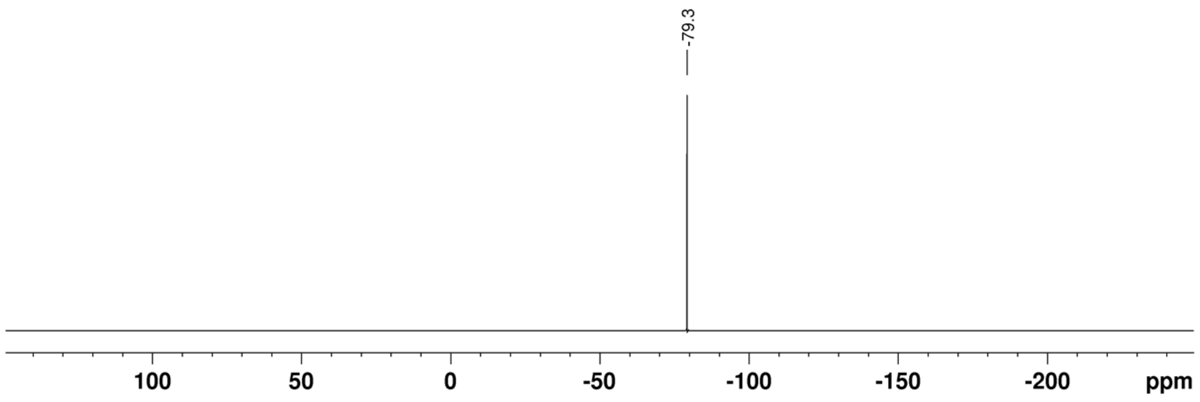

Figure S79. ^{19}F NMR spectrum of **7f**[OTf]$_2$ (CD_3CN, 300 K).

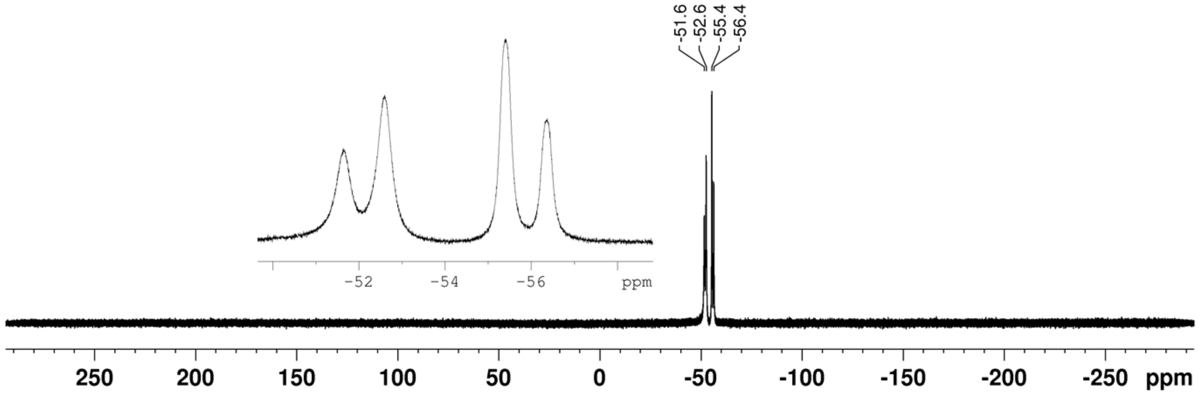

Figure S80. ^{31}P NMR spectrum of **7f**[OTf]$_2$ (CD_3CN, 300 K).

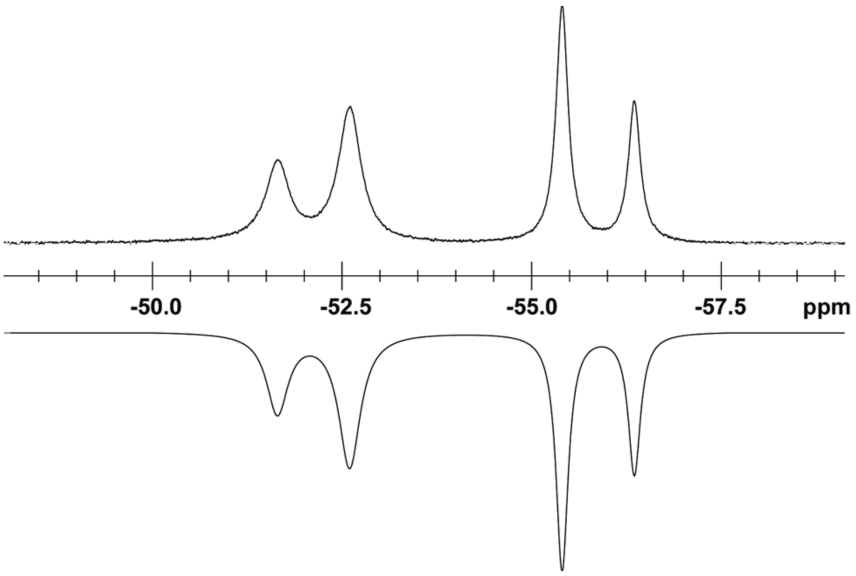

Figure S81. Measured (upwards) and simulated (downwards) $^{31}P\{^1H\}$ NMR spectrum of **7f**[OTf]$_2$ (CD_3CN, 300 K).

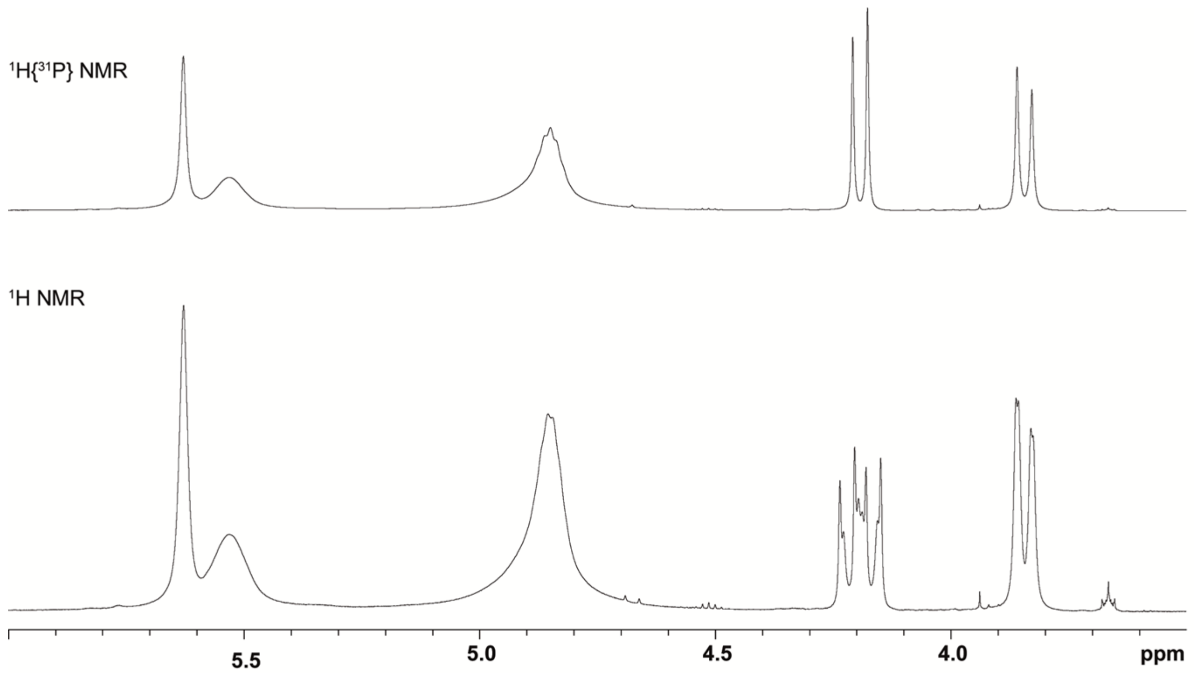

Figure S82. ^{1}H and ^{1}H{^{31}P} NMR spectra of **7f**[OTf]$_2$ (CD_3CN, 300 K).

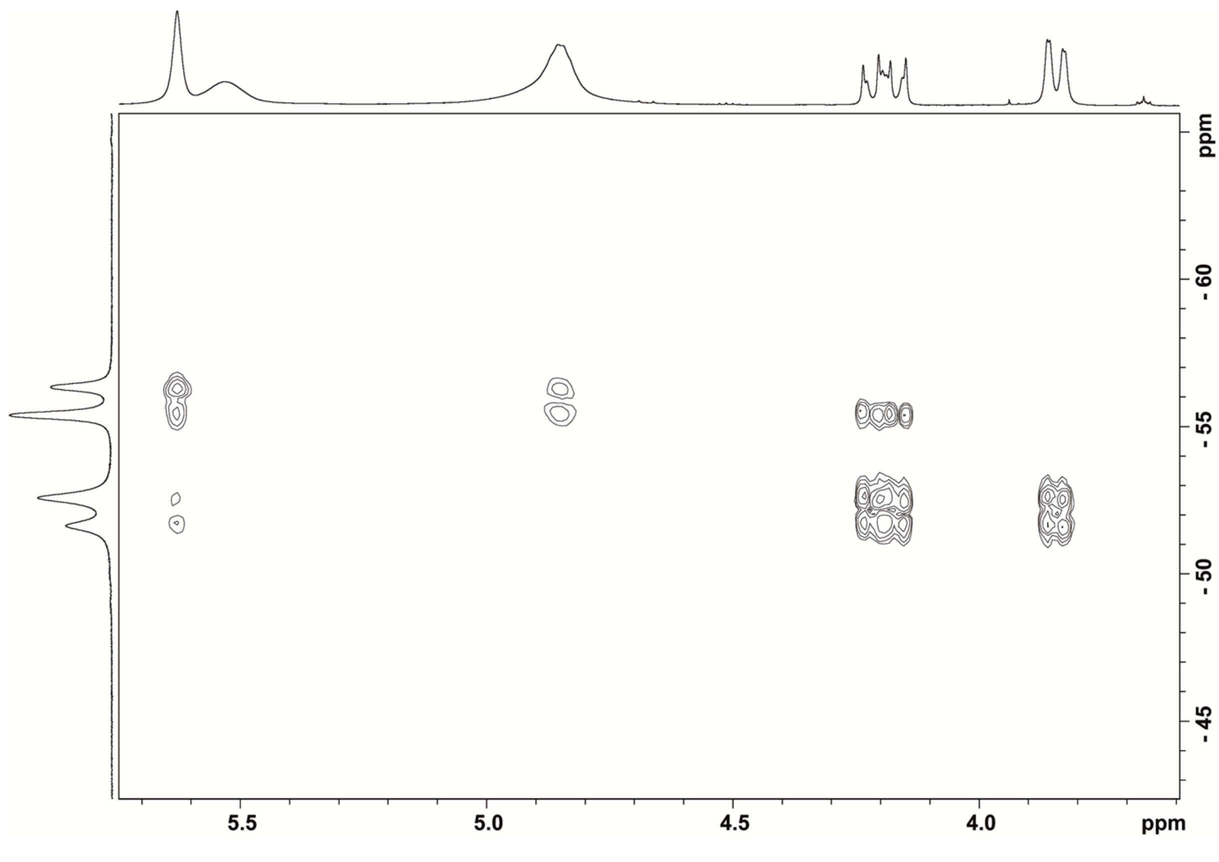

Figure S83. ^{1}H{^{31}P} HMBC NMR spectrum of **7f**[OTf]$_2$ (CD_3CN, 300 K).

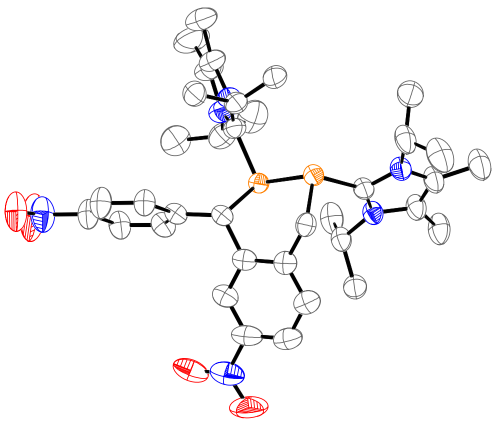

Figure S84. Molecular structures of **7f**$^{2+}$ in **7f**$[OTf]_2$ • CH_3CN • Et_2O; hydrogen atoms, anions, and solvent molecules are omitted for clarity, and thermal ellipsoids are displayed at 50% probability.

S2.19 Preparation of *anti*-1-Imidazoliumyl-4,5-dimethyl-2-(4-nitrophenyl)-1,2,3,6-tetrahydrophosphinine (8f[OTf])

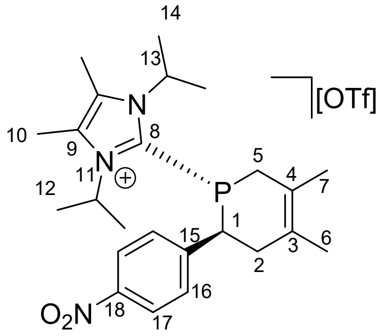

To a mixture of **1c**[OTf] (300 mg, 0.53 mmol, 1.0 equiv.) and 4-nitrobenzaldehyde (399 mg, 2.64 mmol, 5.0 equiv.), CH_2Cl_2 (10 ml) was added, and the yellow solution was stirred at room temperature for ten minutes until a solution of 2,3-dimethylbutadiene (90 µl, 65.0 mg, 0.79 mmol, 1.5 equiv.) in CH_2Cl_2 (5 ml) was added. After 16 hours at room temperature the resulting dark yellow solution was evaporated to dryness *in vacuo*, dissolved in C_6H_5F (3 ml) and Et_2O (15 ml) was added. The occurring yellow solid was filtered off, washed with Et_2O (3x4 ml), and dried *in vacuo* to afford the product as an air- and moisture-sensitive pale-yellow solid. Single crystals suitable for X-ray diffraction analysis were obtained from slow diffusion of Et_2O into a saturated CH_2Cl_2 solution at −30°C.

Yield: 280 mg (92%); **m.p.**: 165-167 °C; **Raman** (100 mW, 100 scans, 298 K, in cm^{-1}): sample showed some fluorescence, 3075 (6), 3036 (7), 2994 (10), 2944 (17), 2930 (15), 2907 (14), 2896 (13), 2884 (12), 1663 (25), 1617 (28), 1597 (49), 1515 (27), 1453 (30), 1429 (27), 1409 (31), 1384 (28), 1345 (100), 1281 (34), 1224 (26), 1191 (29), 1153 (27), 1108 (36), 1032 (32), 1011 (25), 915 (26), 887 (27), 869 (28), 844 (25), 810 (26), 788 (27), 754 (27), 727 (26), 701 (26), 686 (28), 654 (26), 625 (29), 587 (26), 573 (28), 545 (27), 503 (27), 493 (25), 466 (26), 442 (26), 414 (26), 349 (27), 314 (27); **IR** (ATR, 298 K, in cm^{-1}): 3073 (vw), 3034 (vw), 2994 (vw), 2976 (vw), 2942 (vw), 2894 (vw), 2883 (vw), 2827 (vw), 1616 (vw), 1597 (vw), 1513 (m), 1489 (vw), 1455 (vw), 1405 (w), 1374 (vw), 1343 (s), 1320 (vw), 1265 (vs), 1223 (m), 1184 (vw), 1147 (s), 1116 (w), 1090 (w), 1030 (vs), 1011 (w), 960 (vw), 940 (vw), 905 (vw), 885 (vw), 870 (w), 842 (vw), 835 (vw), 809 (vw), 787 (vw), 775 (vw), 755 (w), 727 (vw), 700 (w), 669 (vw), 636 (vs), 571 (w), 543 (vw), 533 (w), 516 (m), 463 (w), 440 (vw); **^{1}H NMR** (500.13 MHz, CD_2Cl_2, 300 K, in ppm): δ = 1.37 (6H, s(br), H12), 1.65 (6H, s(br), H14), 1.84 (3H, s, H6), 1.91 (3H, s, H7), 2.37 (6H, s(br), H10), 2.65 (2H, m, $H2_{eq}$ and $H2_{ax}$), 2.87 (2H, m, $H5_{eq}$ and $H5_{ax}$), 3.76 (1H, m, H1), 5.31 (2H, s(br), H11 and H13), 7.46 (2H, m, H16), 8.18 (2H, m, H17); **^{19}F NMR** (470.59 MHz, CD_2Cl_2, 300 K, in ppm): δ = −78.8 (3F, s, OTf); **^{13}C{^{1}H} NMR** (125.76 MHz, CD_2Cl_2, 300 K, in ppm): δ = 10.7 (2C, s(br), C10), 20.5 (1C, d, ^4J(CP) = 1 Hz, C6), 21.2 (2C, s(br), C12), 21.2 (2C, s(br), C14), 21.2 (1C, d, ^4J(CP) = 5 Hz, C7), 26.2 (1C, d, ^1J(CP) = 17 Hz, C5), 38.9 (1C, d, ^2J(CP) = 9 Hz, C2), 38.9 (1C, d, ^1J(CP) = 9 Hz, C1), 52.8 (1C, s, C11), 52.9 (1C, s, C13), 121.0 (1C, q, ^1J(CF) = 322 Hz, OTf), 122.6 (1C, s, C3), 124.3 (2C, s, C17), 128.8 (2C, d, ^3J(CP) = 9 Hz, C16), 129.7 (1C, d, ^2J(CP) = 6 Hz, C4), 131.7 (2C, s(br), C9), 141.4 (1C, d, ^1J(CP) = 70 Hz, C8), 147.1 (1C, d, ^5J(CP) = 3 Hz, C18), 149.0 (1C, d, ^2J(CP) = 12 Hz, C15); **^{31}P{^{1}H} NMR** (202.46 MHz, CD_2Cl_2, 300 K, in ppm): δ = −47.7 (1P, s(br), P); **elemental analysis**: calcd. for $C_{25}H_{35}F_3N_3O_5PS$: C: 51.99, H: 6.11, N: 7.28, S: 5.55; found: C: 51.11, H: 5.872, N: 7.24, S: 5.357.

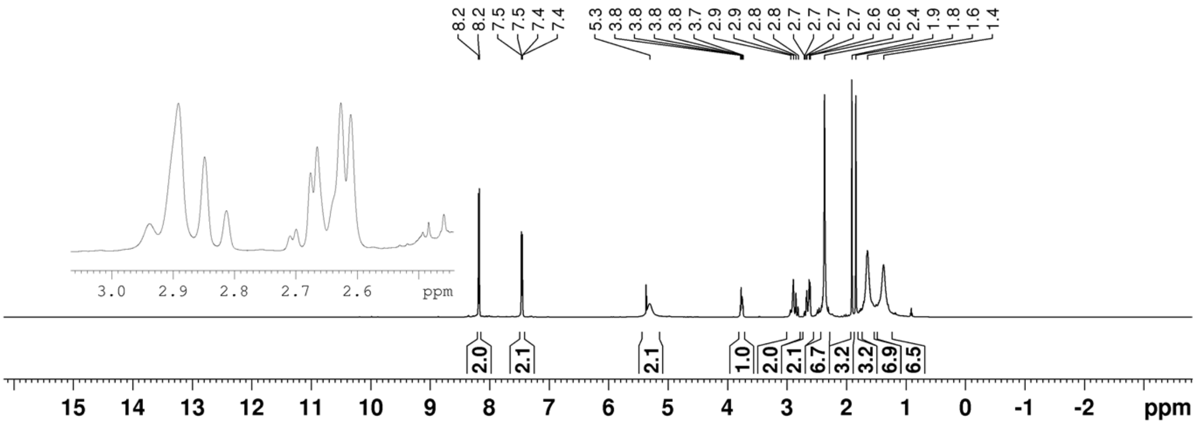

Figure S85. ^{1}H NMR spectrum of **8f**[OTf] (CD_3CN, 300 K).

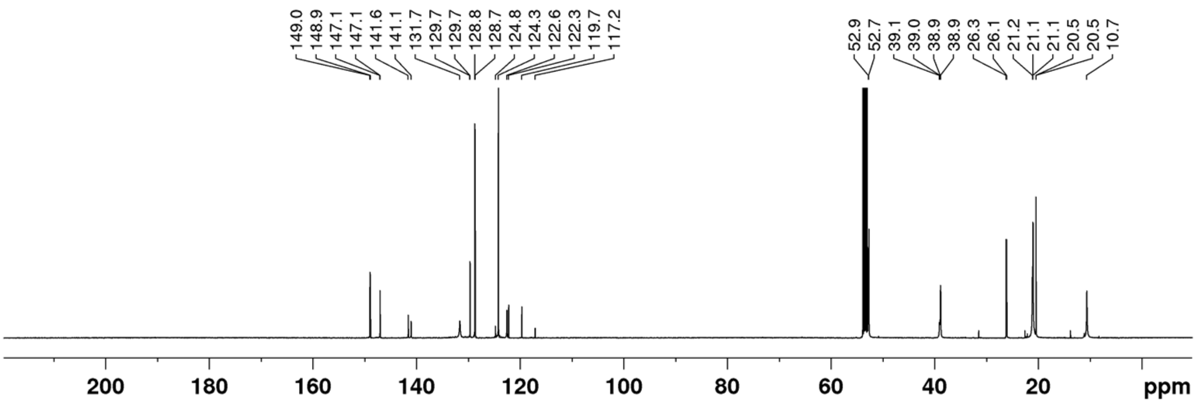

Figure S86. ^{13}C NMR spectrum of **8f**[OTf] (CD_3CN, 300 K).

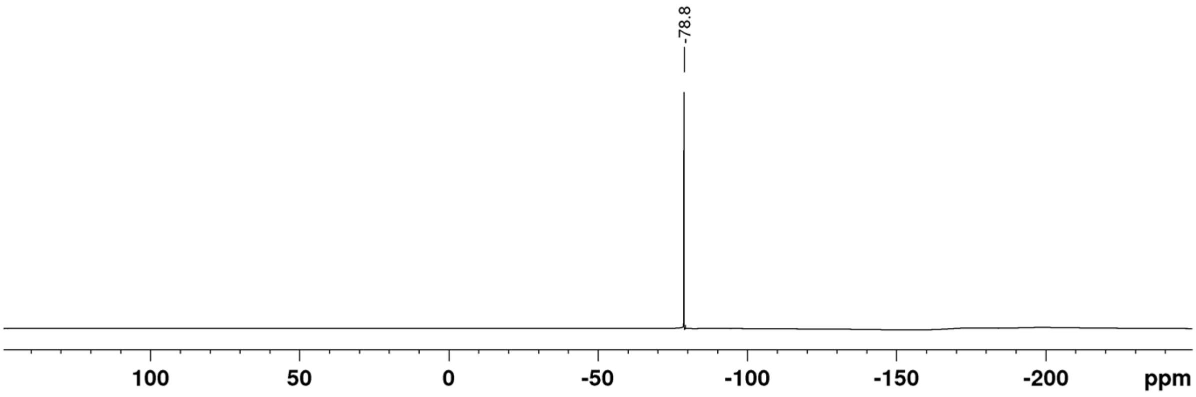

Figure S87. ^{19}F NMR spectrum of **8f**[OTf] (CD_3CN, 300 K).

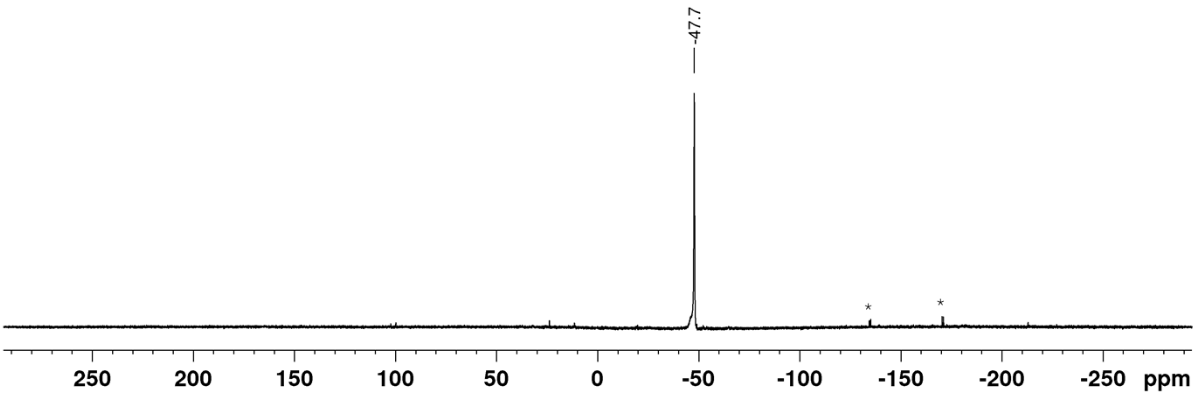

Figure S88. ^{31}P NMR spectrum of **8f**[OTf] (CD_3CN, 300 K). Asterisks mark trace amounts of diphosphirane **4f**$^{2+}$.

S2.20 Preparation of *endo*-2-Imidazoliumyl-3-(4-nitrophenyl)-2-phosphabicyclo[2.2.2]oct-5-ene (9f[OTf])

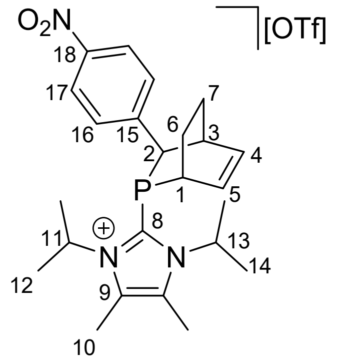

To a mixture of **1c**[OTf] (500 mg, 0.88 mmol, 1.0 equiv.) and 4-nitrobenzaldehyde (665 mg, 4.4 mmol, 5.0 equiv.), CH_2Cl_2 (10 ml) was added, and the yellow solution was stirred at room temperature for ten minutes until a solution of 1,3-cyclohexadiene (89.5 µl, 106 mg, 1.3 mmol, 1.5 equiv.) in CH_2Cl_2 (5 ml) was added. After 16 hours at room temperature the volume of the resulting dark yellow solution was reduced to approximately 2 ml and Et_2O (15 ml) was added, resulting in the precipitation of a yellow solid. After ten minutes at room temperature, the suspension was stored at −30°C for one hour, filtered, and the yellow filtration residue was washed thoroughly with Et_2O, and dried *in vacuo* to afford the product as an air- and moisture-sensitive off-white solid. Single crystals suitable for X-ray diffraction analysis were obtained from slow diffusion of Et_2O into a saturated CH_2Cl_2 solution at room temperature.

Yield: 458 mg (91%); **m.p.**: 206-208 °C (decomp.); **Raman** (100 mW, 100 scans, 298 K, in cm^{-1}): 3077 (6), 3053 (7), 2985 (10), 2947 (21), 2879 (6), 2679 (4), 2449 (4), 1623 (12), 1614 (15), 1593 (40), 1525 (11), 1493 (8), 1447 (13), 1414 (13), 1404 (13), 1386 (10), 1345 (100), 1281 (22), 1251 (8), 1239 (8), 1224 (9), 1195 (12), 1184 (14), 1164 (11), 1108 (23), 1032 (20), 1009 (8), 955 (7), 919 (8), 892 (10), 862 (12), 810 (8), 798 (9), 789 (9), 753 (11), 734 (9), 680 (9), 646 (13), 625 (12), 585 (8), 573 (8), 545 (8), 529 (11), 503 (7), 464 (8), 452 (8); **IR** (ATR, 298 K, in cm^{-1}): 3058 (vw), 2982 (vw), 2946 (vw), 2876 (vw), 1622 (vw), 1604 (vw), 1593 (vw), 1519 (m), 1491 (vw), 1463 (vw), 1445 (vw), 1393 (w), 1377 (vw), 1343 (s), 1262 (vs), 1222 (w), 1195 (vw), 1182 (vw), 1143 (m), 1116 (w), 1106 (w), 1087 (vw), 1060 (vw), 1030 (s), 1013 (vw), 962 (vw), 943 (vw), 918 (w), 907 (vw), 875 (w), 861 (w), 826 (vw), 811 (vw), 797 (vw), 788 (vw), 752 (w), 728 (m), 701 (w), 678 (vw), 636 (vs), 595 (vw), 571 (w), 544 (vw), 516 (w), 500 (w), 452 (vw); **^{1}H NMR** (500.13 MHz, CD_3CN, 300 K, in ppm): δ = 1.21 (1H, m, H7$_{anti/syn}$), 1.41 (6H, s(br), H12), 1.53 (6H, d, ^3J(HH) = 7.1 Hz, H14), 1.81 (1H, m, H7$_{anti/syn}$), 1.88 (1H, m, H6$_{syn}$), 2.13 (1H, m, H6$_{anti}$), 2.32 (6H, s, H10), 3.01 (1H, m, H3), 3.38 (1H, m, H1), 3.81 (1H, s(br), H2), 5.29 (2H, s(br), H11 and H13), 6.65 (1H, m(br), H5), 6.73 (1H, m(br), H4), 7.82 (2H, m, H16), 8.24 (2H, m, H17); **^{19}F NMR** (470.59 MHz, CD_3CN, 300 K, in ppm): δ = −79.3 (3F, s, OTf); **^{13}C{^{1}H} NMR** (125.76 MHz, CD_3CN, 300 K, in ppm): δ = 10.1 (2C, s, C10), 18.3 (1C, d, ^3J(CP) = 9 Hz, C7), 20.1 (2C, s, C14), 20.2 (2C, s(br), C12), 25.9 (1C, d, C6), 29.3 (1C, d, ^1J(CP) = 19 Hz, C1), 37.9 (1C, s, C3), 46.8 (1C, d, ^1J(CP) = 21 Hz, C2), 52.1 (2C, d, ^3J(CP) = 14 Hz, C11 and C13), 121.2 (1C, q, ^1J(CF) = 321 Hz, OTf), 123.7 (2C, s, C9), 123.7 (2C, s, C17), 130.6 (2C, d, ^3J(CP) = 16 Hz, C16), 131.0 (1C, s(br), C15), 131.7 (1C, d, ^2J(CP) = 4 Hz, C5), 135.0 (1C, s, C4), 147.0 (1C, d, ^5J(CP) = 2 Hz, C18), 147.9 (1C, d, ^1J(CP) = 26 Hz, C8); **^{31}P{^{1}H} NMR** (202.46 MHz, CD_3CN, 300 K, in ppm): δ = −28.0 (1P, s(br), P); **elemental analysis**: calcd. for $C_{25}H_{33}F_3N_3O_5PS$: C: 52.17, H: 5.78, N: 7.30, S: 5.57; found: C: 51.98, H: 5.676, N: 7.42, S: 5.527.

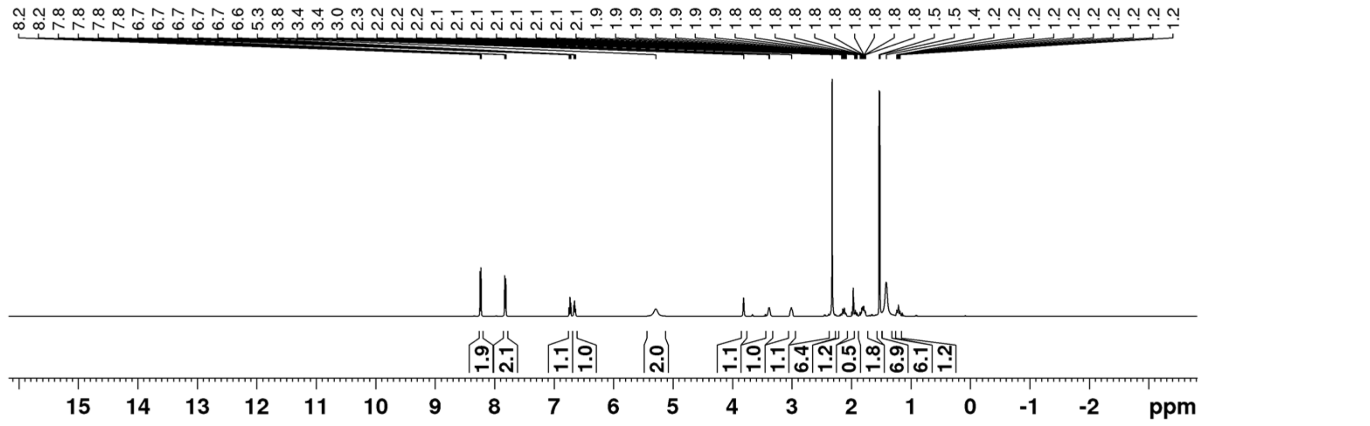

Figure S89. ^{1}H NMR spectrum of **9f**[OTf]$_2$ (CD_3CN, 300 K).

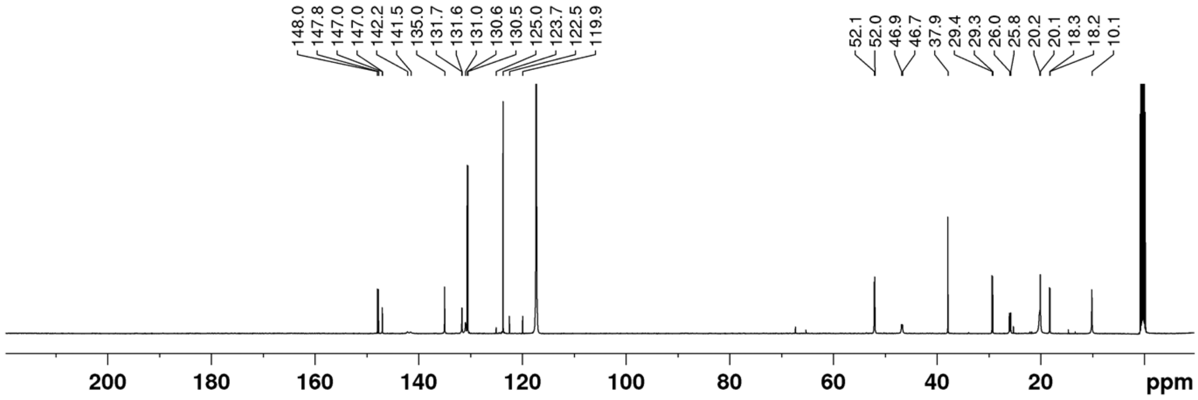

Figure S90. ^{13}C NMR spectrum of **9f**[OTf] (CD_3CN, 300 K).

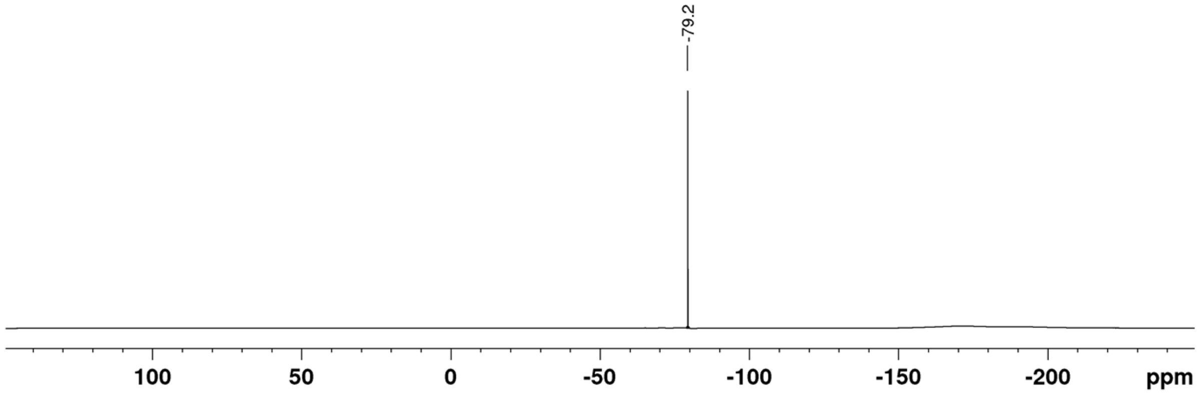

Figure S91. ^{19}F NMR spectrum of **9f**[OTf] (CD_3CN, 300 K).

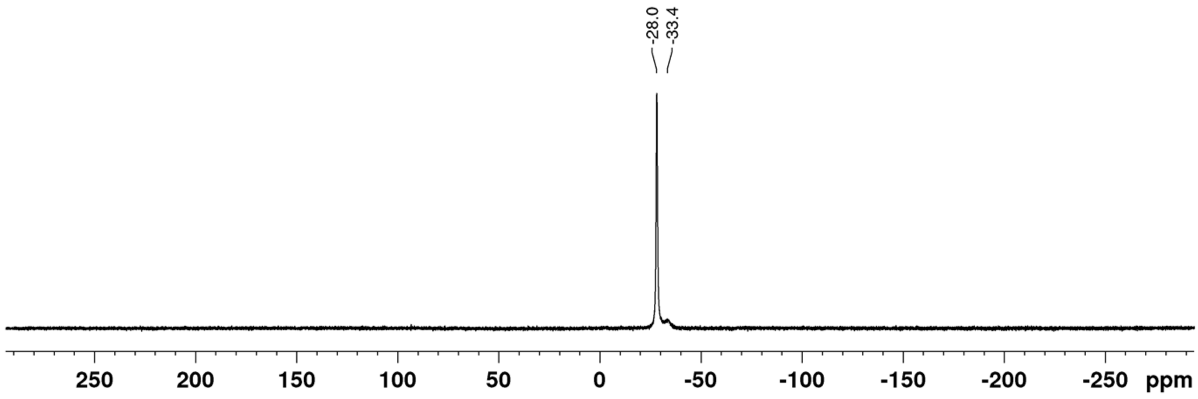

Figure S92. ^{31}P NMR spectrum of **9f**[OTf] (CD_3CN, 300 K).

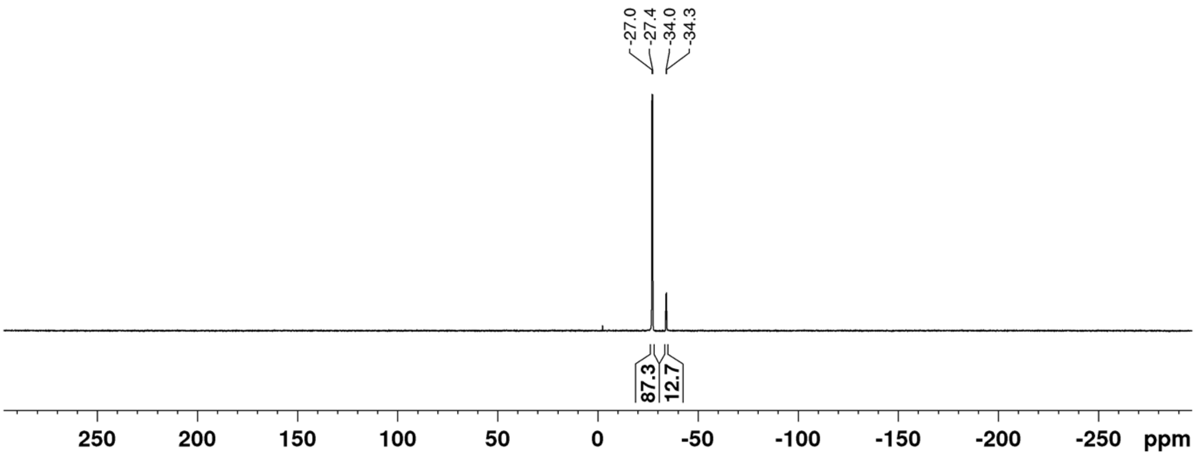

Figure S93. ^{31}P NMR spectrum of **9f**[OTf] (CD_3CN, 240 K).

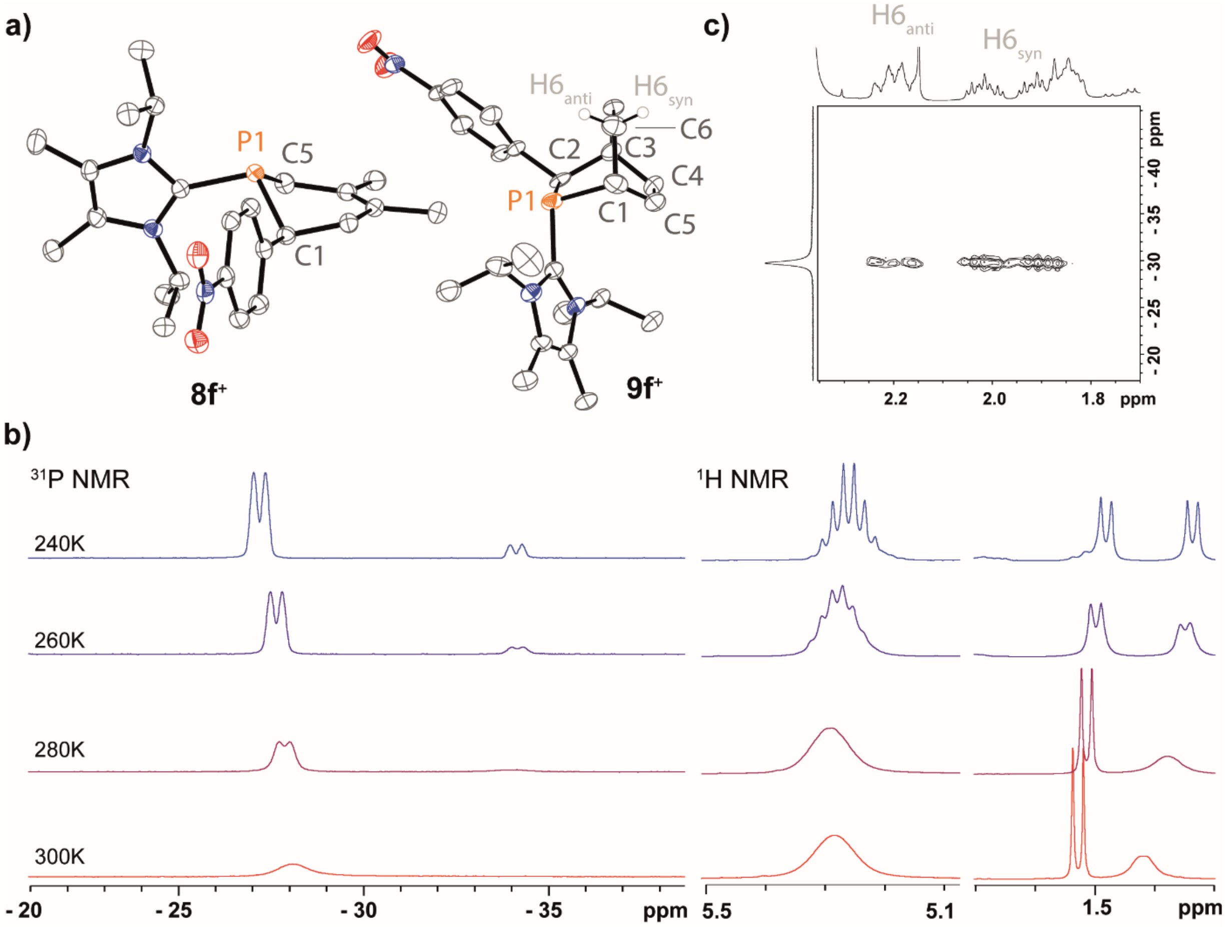

Figure S94. a) Molecular structures of *anti*-**8f**$^+$ and *endo*-**8f**$^+$ in **8f**[OTf] and **8f**[OTf]; selected hydrogen atoms and anions are omitted for clarity, and thermal ellipsoids are displayed at 50% probability; selected bond lengths (Å) and angles (°): for **8f**$^+$: P1–C1 1.854(2), P1–C5 1.8341(2), C1–P1–C5 96.9(1); for **9f**$^+$: P1–C2 1.869(2), P1–C1 1.882(4), C2–C3 1.575(5), P1–C1–C5 111.4(3), P1–C3–C4 109.9(3); b) ^1H and ^{31}P NMR spectra of a solution of **9f**[OTf] at varying temperatures (CD_3CN, 240-300 K); c) $^1H\{^{31}P\}$HMBC spectrum of **9f**[OTf] (CD_3CN, 300 K)

Upon gradual cooling a CD_2Cl_2 solution of **9f**[OTf] from 300 K to 240 K, the broad ^{31}P resonance revolved into two doublets with identical large coupling constants [$^3J(PH)$ = 51 Hz, **Figure S94b**]. Crosspeaks in the $^1H\{^{31}P\}$HMBC spectrum (**Figure S94c**) confirmed the coupling to arise from vicinal P–H6 interactions. Analysis of the coupling constant with respect to dihedral angular dependencies for organophosphorus compounds (based on the Karplus correlation)§ supports assignment to the $H6_{syn}$ proton [∠lone pair–P–C1–C6 ≈ 60°C (gauche conformation), ∠P–C1–C6–$H6_{anti}$ = 50.6(3)°, ∠P–C1–C6–$H6_{syn}$ = 169.2(3)°]. In contrast, a markedly different $^3J(PH)$ coupling constant can be expected from the corresponding *exo*-diastereomer. The additional minor doublet observed at 240 K is therefore attributed to a conformer of **9f**$^+$, arising from

hindered rotation of the ^{i}Pr groups about the N–C^{i}Pr bond of the imidazoliumyl moiety (**Figure S95**). The assignment is further supported by variable-temperature 1H NMR data of **9f**$^{+}$ (300-240 K). Cooling leads to sharpening of the doublet resonance assigned to the CH and CH_3 protons of the ^{i}Pr substituent at the imidazoliumyl group (**Figure S95**). Moreover, the emergence of a second, upfield-shifted resonance for an ^{i}Pr CH proton at 240 K corroborates the presence of the conformer.

§ Qualitative and quantitative Karplus correlations[11] for the ^{3}J(PH) coupling constant have been employed empirically for organophosphorus compounds with phosphorus in oxidation state +V.[6] However, a Karplus-type equation predicting ^{3}J(PH) on the basis of P−H dihedral angle is insufficient for trivalent phosphorus due to effects from lone pair conformation.[12] Hersh et al. described the two-dimensional dependency of the vicinal Phosphorus-Hydrogen NMR coupling constants on lone-pair conformation and the classical Karplus dihedral angle.[12]

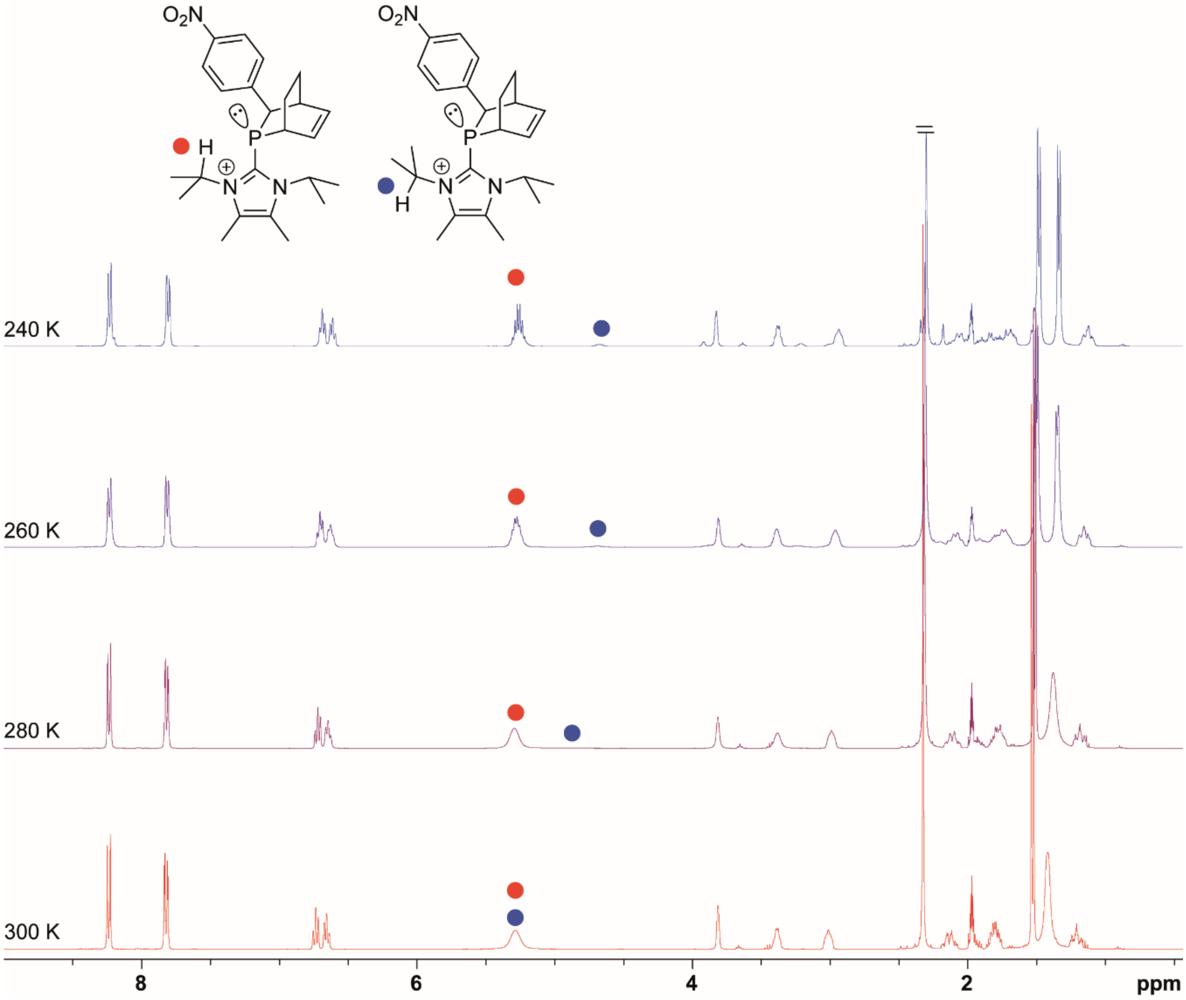

Figure S95. ^{1}H NMR spectra of **9f**[OTf] at varying temperatures (CD_3CN, 240-300 K).

S3 Single Crystal X-ray Diffraction Data

S3.1 General remarks

Suitable single crystals were coated with Paratone-N oil, mounted using a nylon loop and frozen in a cold nitrogen stream. Crystals were measured at 100 K on a Rigaku Oxford Diffraction SuperNova system using Cu K_α radiation (λ = 1.54184 Å) generated by a Nova micro-focus X-ray source. Reflections were collected with an Atlas S2 detector. Data reduction and absorption correction was performed with CrysaAlisPro[13] software. Using Olex2[14], the structures were solved with SHELXS/T[15] by direct methods and refined with SHELXL[16] by least-square minimization against F^2 using first isotropic and later anisotropic thermal parameters for all non-hydrogen atoms. Hydrogen atoms bonded to carbon atoms were added to the structure models on calculated positions using the riding model. All other hydrogen atoms were localized in the difference Fourier map. Images of the structures were produced with Olex2[14] software. All structures have been deposited

with the Cambridge Crystallographic Data Centre (CCDC) and can be accessed free of charge under the numbers 2501246-2501257, 2517773 and 2517774.

S3.2 Refinement details

Table S1. Crystallographic data of **1d**[OTf], **2h**[OTf] and **2i**[OTf].

	**1d**[OTf]	**2h**[OTf]	**2i**[OTf]
Empirical formula	$C_{30}H_{53}F_3N_2O_6P_2S$	$C_{19}H_{21}F_8N_2O_3PS$	$C_{17}H_{30}F_3N_2O_3PS$
Formula weight	688.74	540.41	430.46
Temperature/K	100.00(10)	99.94(13)	100.00(10)
Crystal system	monoclinic	monoclinic	monoclinic
Space group	$P2_1/n$	$P2_1/c$	$P2_1/n$
a/Å	10.58510(10)	9.6269(6)	9.54210(10)
b/Å	18.8359(2)	26.2948(18)	20.0160(2)
c/Å	17.73090(10)	18.1460(13)	11.7739(2)
α/°	90	90	90
β/°	94.9020(10)	92.044(6)	100.8860(10)
γ/°	90	90	90
Volume/Å^3	3522.25(5)	4590.5(5)	2208.29(5)
Z	4	8	4
ρ_{calc}g/cm^3	1.299	1.564	1.295
μ/mm^{-1}	2.172	2.756	2.382
F(000)	1472	2208	912
Crystal size/mm^3	0.628 × 0.484 × 0.346	0.162 × 0.12 × 0.053	0.115 × 0.077 × 0.064
Radiation	Cu Kα (λ = 1.54184)	Cu Kα (λ = 1.54184)	Cu Kα (λ = 1.54184)
2Θ range for data collection/°	6.86 to 153.962	5.92 to 136.496	8.832 to 153.486
Index ranges	-13 ≤ h ≤ 11, -23 ≤ k ≤ 23, -22 ≤ l ≤ 22	-11 ≤ h ≤ 11, -31 ≤ k ≤ 21, -21 ≤ l ≤ 21	-11 ≤ h ≤ 12, -16 ≤ k ≤ 24, -14 ≤ l ≤ 14
Reflections collected	41769	25476	25584
Independent reflections	7369 [R_{int} = 0.0466, R_{sigma} = 0.0267]	8307 [R_{int} = 0.0519, R_{sigma} = 0.0582]	4624 [R_{int} = 0.0387, R_{sigma} = 0.0255]
Data/restraints/parameters	7369/0/403	8307/505/809	4624/0/257
Goodness-of-fit on F^2	1.028	1.015	1.091
Final R indexes [I>=2σ (I)]	R_1 = 0.0431, wR_2 = 0.1160	R_1 = 0.0708, wR_2 = 0.1626	R_1 = 0.0457, wR_2 = 0.1156
Final R indexes [all data]	R_1 = 0.0459, wR_2 = 0.1189	R_1 = 0.0989, wR_2 = 0.1788	R_1 = 0.0501, wR_2 = 0.1181
Largest diff. peak/hole/e Å^{-3}	0.41/-0.58	0.59/-0.66	0.35/-0.43
CCDC	2501252	2501253	2501256

Table S2. Crystallographic data of **4b**[OTf]$_2$ · *o*-$C_6H_4F_2$, **5c**[OTf]$_2$ · *o*-$C_6H_4F_2$ and *syn*-(**2h**)$_2$[OTf]$_2$.

	**4b**[OTf]$_2$ · *o*-$C_6H_4F_2$	**5c**[OTf]$_2$ · *o*-$C_6H_4F_2$	*syn*-(**2h**)$_2$[OTf]$_2$
Empirical formula	$C_{37}H_{49}BrF_8N_4O_6P_2S_2$	$C_{35}H_{54}F_6N_4O_9P_2S_2$	$C_{38}H_{42}F_{16}N_4O_6P_2S_2$
Formula weight	1003.77	914.88	1080.81
Temperature/K	100.01(10)	200.00(10)	100.02(10)
Crystal system	monoclinic	monoclinic	monoclinic
Space group	$P2_1/c$	Pn	$P2_1/c$
a/Å	18.3115(2)	10.49620(10)	33.48865(11)
b/Å	12.4673(2)	16.7168(2)	18.15171(6)
c/Å	19.7589(3)	14.43090(10)	22.02616(6)
α/°	90	90	90
β/°	102.7040(10)	90.0260(10)	92.3847(3)
γ/°	90	90	90
Volume/Å^3	4400.43(11)	2532.09(4)	13377.58(7)
Z	4	2	12
ρ_{calc}g/cm^3	1.515	1.2	1.61
μ/mm^{-1}	3.578	2.156	2.837
F(000)	2064	960	6624
Crystal size/mm^3	0.285 × 0.236 × 0.065	0.347 × 0.197 × 0.089	0.218 × 0.139 × 0.106
Radiation	Cu Kα (λ = 1.54184)	Cu Kα (λ = 1.54184)	Cu Kα (λ = 1.54184)
2Θ range for data collection/°	4.946 to 153.434	5.286 to 154.206	5.282 to 153.606
Index ranges	-23 ≤ h ≤ 16, -15 ≤ k ≤ 15, -24 ≤ l ≤ 24	-13 ≤ h ≤ 13, -21 ≤ k ≤ 20, -12 ≤ l ≤ 18	-42 ≤ h ≤ 42, -22 ≤ k ≤ 22, -27 ≤ l ≤ 23
Reflections collected	49573	25804	164737
Independent reflections	9214 [R_{int} = 0.0355, R_{sigma} = 0.0252]	7580 [R_{int} = 0.0346, R_{sigma} = 0.0316]	28048 [R_{int} = 0.0308, R_{sigma} = 0.0195]
Data/restraints/parameters	9214/0/553	7580/153/743	28048/436/2090
Goodness-of-fit on F^2	1.028	1.033	1.016
Final R indexes [I>=2σ (I)]	R_1 = 0.0271, wR_2 = 0.0683	R_1 = 0.0470, wR_2 = 0.1320	R_1 = 0.0334, wR_2 = 0.0859
Final R indexes [all data]	R_1 = 0.0294, wR_2 = 0.0698	R_1 = 0.0515, wR_2 = 0.1394	R_1 = 0.0381, wR_2 = 0.0894
Largest diff. peak/hole/e Å^{-3}	0.49/-0.40	0.19/-0.29	0.51/-0.56
Flack parameter	-	0.04(3)	-
CCDC	2501248	2501246	2501249

Table S3. Crystallographic data of *anti*-(**2h**)$_2$[OTf]$_2$, **7e**[OTf]$_2$ · CH_3CN and **7f**[OTf]$_2$ · CH_3CN · Et_2O.

	anti-(**2h**)$_2$[OTf]$_2$	**7e**[OTf]$_2$ · CH_3CN	**7f**[OTf]$_2$ · CH_3CN · Et_2O
Empirical formula	$C_{38}H_{42}F_{16}N_4O_6P_2S_2$	$C_{42}H_{53}F_6N_7O_6P_2S_2$	$C_{44}H_{63}F_6N_7O_{11}P_2S_2$
Formula weight	1080.81	991.97	1106.07
Temperature/K	100.00(10)	100.00(10)	99.98(10)
Crystal system	monoclinic	triclinic	hexagonal
Space group	$P2_1/n$	P-1	$P6_5$
a/Å	10.94280(10)	10.2822(3)	13.7455(3)
b/Å	14.67850(10)	13.2602(4)	13.7455(3)
c/Å	14.0224(2)	17.8827(5)	48.9066(10)
α/°	90	94.291(2)	90
β/°	97.4910(10)	97.156(3)	90
γ/°	90	90.210(3)	120
Volume/Å^3	2233.11(4)	2412.22(12)	8002.4(4)
Z	2	2	6
ρ_{calc}g/cm^3	1.607	1.366	1.377
μ/mm^{-1}	2.833	2.283	2.192
F(000)	1104	1036	3480
Crystal size/mm^3	0.235 × 0.146 × 0.133	0.254 × 0.179 × 0.056	0.364 × 0.045 × 0.037
Radiation	Cu Kα (λ = 1.54184)	Cu Kα (λ = 1.54184)	Cu Kα (λ = 1.54184)
2Θ range for data collection/°	8.76 to 153.254	4.994 to 153.468	7.426 to 136.418
Index ranges	-13 ≤ h ≤ 13, -18 ≤ k ≤ 17, -17 ≤ l ≤ 17	-12 ≤ h ≤ 12, -16 ≤ k ≤ 16, -22 ≤ l ≤ 17	-16 ≤ h ≤ 13, -15 ≤ k ≤ 16, -58 ≤ l ≤ 58
Reflections collected	24861	26815	35270
Independent reflections	4675 [R_{int} = 0.0253, R_{sigma} = 0.0168]	9990 [R_{int} = 0.0261, R_{sigma} = 0.0304]	9735 [R_{int} = 0.0557, R_{sigma} = 0.0480]
Data/restraints/parameters	4675/66/343	9990/677/772	9735/397/713
Goodness-of-fit on F^2	1.04	1.019	1.047
Final R indexes [I>=2σ (I)]	R_1 = 0.0386, wR_2 = 0.1037	R_1 = 0.0454, wR_2 = 0.1205	R_1 = 0.0613, wR_2 = 0.1556
Final R indexes [all data]	R_1 = 0.0418, wR_2 = 0.1071	R_1 = 0.0514, wR_2 = 0.1260	R_1 = 0.0748, wR_2 = 0.1664
Largest diff. peak/hole/e Å^{-3}	0.45/-0.41	0.82/-0.47	0.44/-0.32
Flack Parameter	-	-	-0.011(15)
CCDC	2501250	2501254	2501247

Table S4. Crystallographic data of **8f**[OTf], **9f**[OTf], and *syn*-(**2e**)$_2$[OTf]$_2$.

	**8f**[OTf]	**9f**[OTf]	*syn*-(**2e**)$_2$[OTf]$_2$
Empirical formula	$C_{25}H_{35}F_3N_3O_5PS$	$C_{25}H_{33}F_3N_3O_5PS$	$C_{40}H_{50}F_6N_6O_6P_2S_2$
Formula weight	577.59	575.57	950.92
Temperature/K	100.01(10)	100.00(10)	100.00(10)
Crystal system	orthorhombic	monoclinic	monoclinic
Space group	$Pca2_1$	$P2_1/c$	C2/c
a/Å	21.5490(2)	10.9283(4)	25.0983(7)
b/Å	8.46980(10)	14.6147(3)	9.1242(2)
c/Å	15.24110(10)	16.8837(6)	20.5645(4)
α/°	90	90	90
β/°	90	95.322(4)	109.348(2)
γ/°	90	90	90
Volume/Å^3	2781.74(5)	2684.94(15)	4443.34(19)
Z	4	4	4
ρ_{calc}g/cm^3	1.379	1.424	1.421
μ/mm^{-1}	2.107	2.183	2.447
F(000)	1216	1208	1984
Crystal size/mm^3	0.222 × 0.133 × 0.084	0.146 × 0.052 × 0.045	0.164 × 0.081 × 0.046
Radiation	Cu Kα (λ = 1.54184)	Cu Kα (λ = 1.54184)	Cu Kα (λ = 1.54184)
2Θ range for data collection/°	8.206 to 153.67	8.016 to 136.502	7.466 to 153.692
Index ranges	-27 ≤ h ≤ 26, -10 ≤ k ≤ 7, -19 ≤ l ≤ 18	-13 ≤ h ≤ 13, -17 ≤ k ≤ 13, -16 ≤ l ≤ 20	-29 ≤ h ≤ 31, -11 ≤ k ≤ 11, -19 ≤ l ≤ 25
Reflections collected	28113	20867	20527
Independent reflections	5720 [R_{int} = 0.0256, R_{sigma} = 0.0196]	4874 [R_{int} = 0.0592, R_{sigma} = 0.0486]	4651 [R_{int} = 0.0470, R_{sigma} = 0.0377]
Data/restraints/parameters	5720/1/351	4874/1/357	4651/0/286
Goodness-of-fit on F^2	1.05	1.072	1.022
Final R indexes [I>=2σ (I)]	R_1 = 0.0275, wR_2 = 0.0737	R_1 = 0.0712, wR_2 = 0.1887	R_1 = 0.0415, wR_2 = 0.1056
Final R indexes [all data]	R_1 = 0.0279, wR_2 = 0.0741	R_1 = 0.0861, wR_2 = 0.1999	R_1 = 0.0545, wR_2 = 0.1148
Largest diff. peak/hole/e Å^{-3}	0.50/-0.20	1.25/-0.35	0.72/-0.36
Flack Parameter	-0.025(6)		
CCDC	2501251	2501255	2501257

Table S5. Crystallographic data of **4h**[OTf]$_2$ and **6h**[OTf]$_3$ · C_6H_5F.

	**4h**[OTf]$_2$	**6h**[OTf]$_3$ · C_6H_5F
Empirical formula	$C_{31}H_{41}F_{11}N_4O_6P_2S_2$	$C_{49}H_{66}F_{15}N_6O_9P_3S_3$
Formula weight	900.74	1357.16
Temperature/K	100.00(10)	100.0(2)
Crystal system	tetragonal	monoclinic
Space group	$P4_1$	P21/c
a/Å	13.68062(4)	14.82790(10)
b/Å	13.68062(4)	15.67600(10)
c/Å	22.05367(11)	26.6476(2)
α/°	90	90
β/°	90	99.5860(10)
γ/°	90	90
Volume/Å^3	4127.55(3)	6107.54(8)
Z	4	4
ρ_{calc}g/cm^3	1.449	1.476
μ/mm^{-1}	2.763	2.767
F(000)	1856	2808
Crystal size/mm^3	0.227 × 0.195 × 0.174	0.398 × 0.121 × 0.072
Radiation	Cu Kα (λ = 1.54184)	Cu Kα (λ = 1.54184)
2Θ range for data collection/°	6.46 to 153.326	6.044 to 153.518
Index ranges	-17 ≤ h ≤ 11, -17 ≤ k ≤ 17, -26 ≤ l ≤ 27	-18 ≤ h ≤ 17, -19 ≤ k ≤ 14, -32 ≤ l ≤ 33
Reflections collected	46471	66528
Independent reflections	8443 [R_{int} = 0.0261, R_{sigma} = 0.0187]	12803 [R_{int} = 0.0370, R_{sigma} = 0.0252]
Data/restraints/parameters	8443/223/590	12803/481/877
Goodness-of-fit on F^2	1.033	1.038
Final R indexes [I>=2σ (I)]	R_1 = 0.0273, wR_2 = 0.0710	R1 = 0.0575, wR_2 = 0.1668
Final R indexes [all data]	R_1 = 0.0276, wR_2 = 0.0712	R1 = 0.0654, wR_2 = 0.1766
Largest diff. peak/hole/e Å^{-3}	0.91/-0.29	0.99/-0.62
Flack Parameter	-0.004(4)	
CCDC	2517773	2517774

In the structure refinements of **2h**[OTf], **5c**$[OTf]_2$ · *o*-$C_6H_4F_2$, *syn*-(**2h**)$_2$$[OTf]_2$, *anti*-(**2h**)$_2$[OTf]2, **7e**$[OTf]_2$ · CH_3CN and **7f**$[OTf]_2$ · CH_3CN · Et_2O SADI, SIMU, RIGU and ISOR restraints were applied to solve occurring disorders of the triflates anions, ^{i}Pr groups of the imidazoliumyl substituents, and solvent molecules. The crystal of **5c**$[OTf]_2$ · *o*-$C_6H_4F_2$ was obtained, measured and refined as an inversion twin (BASF = 0.04(3)). In the refinement of **7e**$[OTf]_2$ · CH_3CN one triflate anion was found to be disordered over three positions and the SUMP command has been used to determine the respective occupancy in the crystal lattice. In the refinement of **6h**$[OTf]_3$ · C_6H_5F the solvent molecule was found to be disordered over three positions and the SUMP command has been used to determine the respective occupancy in the crystal lattice. The PLATON/SQUEEZE[17] extension in Olex2 was used for a solvent mask in the refinement of **5c**$[OTf]_2$ · *o*-$C_6H_4F_2$ (solvent mask for 1 *o*-$C_6H_4F_2$ per asymmetric unit).

S4 Computational Details

The geometries and energies of all systems included in this study were fully optimized without symmetry constrains at the RI-BP86-D4/def2-TZVP level of theory.[18-21] The calculations have been performed by using the program ORCA[22] version 6.0 For the calculations we have used the BP86[18,19] functional with the D4 correction for dispersion[20] and the triple-ζ def2-TZVP basis set.[21] In order to reproduce solvent effects, we have used the conductor-like continuum model CPCM (for CH_3CN),[23] which is a variant of the dielectric continuum solvation models. The minimum nature of the complexes and compounds have been confirmed by doing frequency calculations. The transition states were initially located using the NEB tool[24] of ORCA 6.0**.** They only present one negative frequency that corresponds to the movement of atoms connecting the intermediates. The ΔG energies were computed at normal conditions**.**

The delocalized frontier molecular orbitals were computed at the same level of theory and represented using the 0.04 a.u. isosurface by means of the Gaussview 5.0 program.[25]

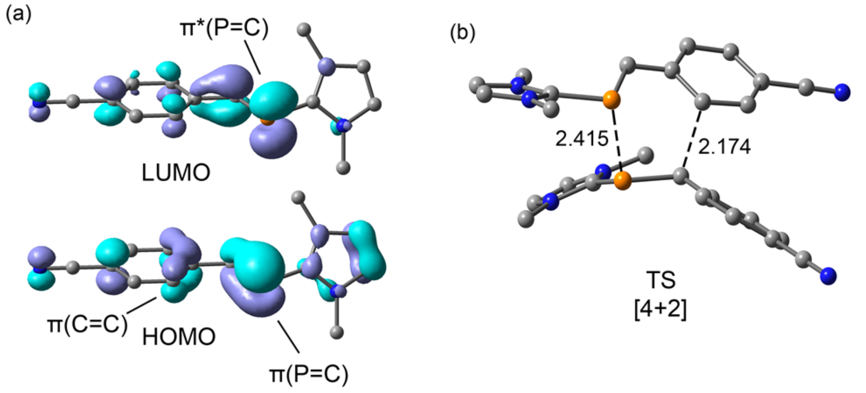

Figure S96. Rationalization of the [4+2] cycloaddition mechanism. (a) Frontier Molecular Orbitals (FMOs) of a molecule of **2e**$^{+}$ (truncated model), showing the HOMO and LUMO. (b) Optimized geometry of the transition state for the [4+2] cycloaddition, illustrating the concerted formation of the two new P–P and C–C bonds with their corresponding interatomic distances in Å.

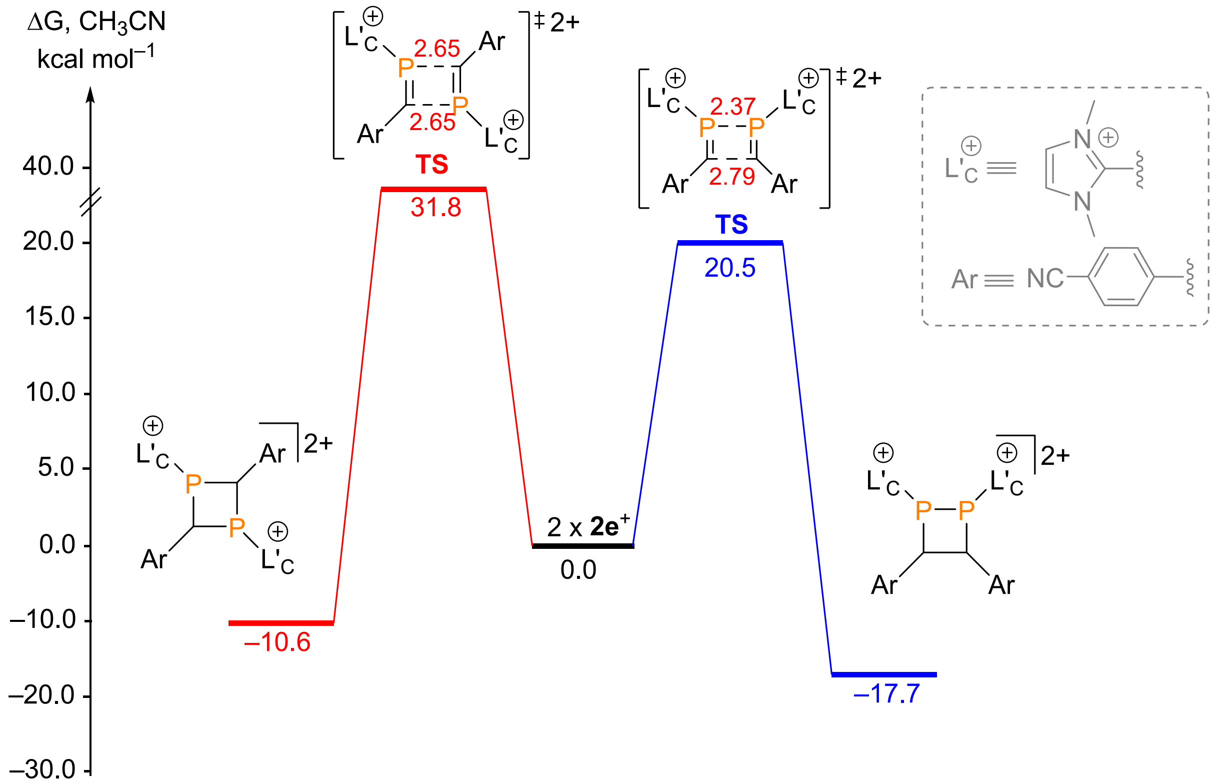

Figure S97. Calculated free energy profile (RI-BP86-D4/def2-TZVP, COSMO = CH_3CN) for [2+2] cycloadditions of two equiv. **2e**$^+$ to form 1,3-diphosphetane (red pathway) and 1,2-diphosphetane (blue pathway). All energies are reported in kcal mol^{-1} and are relative to two isolated molecules of **2e**$^+$.

S5 References

References

1 P. Royla, K. Schwedtmann, Z. Han, J. Fidelius, D. P. Gates, R. M. Gomila, A. Frontera and J. J. Weigand, *J. Am. Chem. Soc.*, 2023, **145**, 10364.

2 I. Kownacki, B. Marciniec, H. Steinberger, M. Kubicki, M. Hoffmann, A. Ziarko, K. Szubert, M. Majchrzak and S. Rubinsztajn, *Applied Catalysis A: General*, 2009, **362**, 106.

3 J. A. Pople, W. G. Schneider and H. J. Bernstein, *Can. J. Chem.*, 1957, **35**, 1060.

4 J. D. Protasiewicz and C. Hering-Junghans, in *Encyclopedia of inorganic and bioinorganic chemistry*, ed. R. A. Scott, Wiley, Chichester, 2012, vol. 13, pp. 1–27.

5 a) D. Weber and E. Fluck, *Z. Anorg. Allg. Chem.*, 1976, **424**, 103; b) D. Bevern, H. Görls, S. Krieck and M. Westerhausen, *Z. Anorg. Allg. Chem.*, 2020, **646**, 948; c) D. Bevern, F. E. Pröhl, H. Görls, S. Krieck and M. Westerhausen, *Organometallics*, 2021, **40**, 1744; d) F. Zurmühlen and M. Regitz, *Angew. Chem. Int. Ed. Engl.*, 1987, **26**, 83;

6 O. Kühl, *Phosphorus-31 NMR Spectroscopy*, Springer Berlin Heidelberg, Berlin, Heidelberg, 2009.

7 J.-P. Albrand, D. Gagnaire, M. Picard and J.-B. Robert, *Tetrahedron Lett.*, 1970, **11**, 4593.

8 J. P. Albrand, D. Gagnaire and J. B. Robert, *Chem. Commun. (London)*, 1968, **0**, 1469.

9 L. D. Quin and T. P. Barket, *J. Am. Chem. Soc.*, 1970, **92**, 4303.

10 C. Giessner-Prettre and B. Pullman, *J. Theor. Biol.*, 1974, **48**, 425.

11 M. Karplus, *J. Am. Chem. Soc.*, 1963, **85**, 2870.

12 W. H. Hersh, S. T. Lam, D. J. Moskovic and A. J. Panagiotakis, *J. Org. Chem.*, 2012, **77**, 4968.

13 Oxford Diffraction/Agilent Technologies UK Ltd, CrysAlisPRO, Yarnton, England, 2016.

14 O. V. Dolomanov, L. J. Bourhis, R. J. Gildea, J. A. K. Howard and H. Puschmann, *J. Appl. Crystallogr.*, 2009, **42**, 339.

15 G. M. Sheldrick, *Acta Crystallogr. A Found. Adv.*, 2015, **71**, 3.

16 G. M. Sheldrick, *Acta Crystallogr. C Struct. Chem.*, 2015, **71**, 3.

17 A. L. Spek, *J. Appl. Crystallogr.*, 2003, **36**, 7.

18 J. P. Perdew, *Phys. Rev. B*, 1986, **33**, 8822–8824.

19 A. D. Becke, *J. Chem. Phys.*, 1996, **104**, 1040–1046.

20 S. Grimme, J. Antony, S. Ehrlich and H. Krieg, *J. Chem. Phys.*, 2010, **132**, 154104,

21 F. Weigend and R. Ahlrichs, *Phys. Chem. Chem. Phys.*, 2005, **7**, 3297-3305.

22 F. Neese, F. Wennmohs, U. Becker and C. Riplinger, *J. Chem. Phys.*, 2020, **152**, 224108..

23 Y. Takano and K. N. Houk, *J. Chem. Theory Comput.*, 2005, **1**, 70–77.

24 G. Henkelman, B. P. Uberuaga and H. Jónsson, *J. Chem. Phys.*, 2000, **113**, 9901–9904

25 *GaussView, Version 6.1*, R. Dennington, T. A. Keith, and J. M. Millam, Semichem Inc., Shawnee Mission, KS, 2016.

S6 Cartesian Coordinates

Structures of Figure 5

2e⁺

P	5.38030489489942	6.23129554035830	11.89219558527914
N	6.63196064233693	3.95291010388624	13.16699615851571
N	4.52469394918269	4.20062851085527	13.65960858999048
C	5.52903532261594	4.70535767162106	12.89133878594344
C	4.98882875523640	3.13172560559823	14.39659843239412
C	6.30884228741818	2.97531617324594	14.08686015594802
C	3.15168926694264	4.71045286574655	13.71203041947594
C	7.97799296326079	4.14872271545820	12.62251028248819
H	8.09153571293642	5.19067270028432	12.30722413754783
C	5.99565622126722	5.64605112404501	10.40923981025296
C	6.18203666294322	6.43805542263933	9.21916389017029
C	6.58999401667005	5.79181325748292	8.02869828958567
C	5.96733836322992	7.83769122423047	9.19023900594500
C	6.76989003411625	6.50499291268126	6.85306765744064
H	6.75893612989288	4.71372473027755	8.03587833984717
C	6.14370526042778	8.55700515892941	8.02327473830892
H	5.66634677720199	8.36267465290603	10.09906859769253
C	6.54684186869614	7.89461593338142	6.84183817827574
H	7.08245114271748	6.00204526378051	5.93878039212584

H	5.97983466989989	9.63377985211367	8.00365606456109
C	6.73252683224948	8.63806691002979	5.63908471065297
N	6.88312383630461	9.24655577255724	4.65639434291315
H	6.24848430435266	4.58735644361231	10.29926881060921
H	4.34894346351743	2.57424084583401	15.07010166639497
H	7.03812981267004	2.25746903126672	14.44278980912718
H	8.70814077734417	3.92137981950134	13.40674510791236
H	8.14553007179398	3.48567218645994	11.76464178428241
H	3.00426894081620	5.42076320510566	12.89134401982126
H	2.45129395580998	3.87525062872042	13.59771100874873
H	2.97786713324916	5.21569737739080	14.67008829774891

TS1

P	-1.06029404074407	-0.88607067709494	-0.20709175995378
C	-1.45198535312194	-0.42604696124349	-1.93337235693152
C	0.72221824437906	-0.99693117652806	-0.12074940819996
N	-2.71329089166475	-0.70221589530834	-2.39816579272412
N	-0.76262459440204	0.06286963893631	-3.00818987300253
C	1.24479058283112	-1.96568093870188	0.84642513714092
H	1.25270459118384	-0.97675685113620	-1.07154489335626
C	-2.80412209934760	-0.38685354857021	-3.73415004697131
C	-1.58263150497870	0.08577406582295	-4.11738234495669
C	2.42606488867807	-2.67440750025395	0.54909065976845
C	0.62283814256920	-2.17811203309514	2.09346169669176
H	-3.71277546965125	-0.53916195712041	-4.30526453262171
H	-1.22719807572332	0.41721470096298	-5.08647784453888
C	2.96458209267956	-3.57452312848696	1.45955771186776
H	2.92275173536539	-2.52292343446475	-0.41101667139230
C	1.15587402929797	-3.06812766259708	3.01335088156363
H	-0.29319966937845	-1.63994361676140	2.35135858899486
C	2.33400426507301	-3.77712567280823	2.70175444023792
H	3.87325488668346	-4.12598134332836	1.22008350549368
H	0.67326717870648	-3.22953151190403	3.97660308458142
C	2.87908337446426	-4.69774843353183	3.64531620112602
N	3.31822665709765	-5.44803397400600	4.42124819718456
H	3.40281130797780	-0.19755552620675	1.16137096215454
C	2.95532526508848	0.61511855080900	0.59104010501655
C	1.55280291705554	0.84475653288610	0.68330044205341
C	3.74596286828976	1.40009802945125	-0.22516270106635
C	0.98500341038489	2.00987203401960	0.02241908945785
H	1.07021681190596	0.53103616511117	1.61468764565021
C	3.17816494080618	2.52408756650433	-0.91687767840634
C	5.13531607084446	1.11127919346331	-0.37483139288976
C	-0.38011184635356	2.29032808460516	0.12960682613035
C	1.84777993884404	2.81933964914101	-0.78572703571667
H	3.82910695732281	3.14768863752844	-1.52967122669524
N	6.26393809578479	0.86189510938800	-0.52229447006035
P	-1.34278503307898	1.15867938976796	1.04723830039724
H	-0.78250115885375	3.16909096297254	-0.37161974865358
H	1.43064675306075	3.69165979933433	-1.29195653295899
C	-3.07987362290648	1.62201534584897	0.72336408820297
N	-4.04357251173168	1.34596586925702	1.65008171031432
N	-3.72216823491122	2.22439616664696	-0.32036894683235
C	-5.26744732686850	1.77253351837799	1.19491146763199
C	-5.06666303104634	2.33046288616976	-0.03740604651460
H	-6.17444794298807	1.66039188441182	1.77825340658119
H	-5.76466488551438	2.80492458584637	-0.71816453145672
C	-3.83492357578438	0.67966803506435	2.94463910997302
H	-3.86261852509580	-0.40956387278403	2.81743677632089
H	-2.86679811531172	0.98147459592623	3.35770468677028
H	-4.63449191759357	0.99049015062172	3.62456885503744
C	-3.12461057798749	2.74879757169581	-1.55020386266896
H	-2.87031351709062	3.80936133104505	-1.42561286041033
H	-2.22224920259006	2.18059465397232	-1.79096831270845
H	-3.85417444415236	2.65051378909698	-2.36097037763247
C	0.65953235691111	0.41105439217605	-3.08541935660238
H	0.97384474921721	0.92217604150657	-2.17375336916741
H	1.25996894505132	-0.49311506271403	-3.25047430689341
H	0.79946440170055	1.08715276691417	-3.93508918849656
C	-3.81601852237345	-1.28127048265110	-1.62402507582886
H	-3.41876751020967	-2.00847289858967	-0.90733827743626
H	-4.36668974696911	-0.49839696032003	-1.08674579775353
H	-4.49428883083136	-1.78931007507578	-2.31732318484464

INT1

C	5.36106283797763	9.18749351905648	14.28036812349980
N	6.27221848143246	9.94296073464459	13.60236056763078
P	4.25883102245527	7.81458134731658	13.75733762326826

P	5.55974873938335	6.62744603429145	12.39040001623705
N	5.28314647466022	9.71987442547120	15.53245067261439
C	6.13286130788732	10.79459431549554	15.63637363982218
N	6.58861230503952	4.31468963627235	13.63222808536545
C	6.75781820914411	10.93113462926581	14.42864051060565
N	4.42761302915586	4.12660717787037	13.41324016552183
C	4.37755774391937	9.30098207978270	16.60964118055387
H	4.02865367622139	8.28322963883260	16.41178912022821
N	-1.25447145897472	9.47184921504900	8.78181352204830
N	0.08603641171962	13.24429666312338	16.75158698098483
C	6.73106444099501	9.75982445529268	12.22229207799547
H	5.91337912480329	9.38775451856168	11.59795158408030
C	5.47325709030078	4.97818086852326	13.20980506068649
C	4.88623559647229	2.95065280594590	13.95970483836869
C	6.24139039044189	3.06937275076531	14.09987457659135
C	3.02141412471783	4.39707385488353	13.11486180566166
H	2.89269280520253	4.56556483967201	12.03980658468554
C	7.96522823612723	4.82375566571524	13.62592618858579
H	8.02753073897995	5.67453800923699	12.93993071328896
C	4.46393631415173	6.43926900638357	10.97788299992586
C	3.24012224713470	6.97977198211004	10.68296237865096
C	2.37438861977112	7.76488319480678	11.66803789724887
C	3.20910626905310	8.77253092679502	12.51162948685420
H	3.84329916277478	9.33483366117341	11.81282259746804
C	2.72258665859340	6.78461711873524	9.34610825436216
H	3.30438706180035	6.16059115780000	8.66561450717717
C	1.58732492576090	7.37767252688721	8.90207059538160
H	1.24082980718718	7.21853218065128	7.88094950202776
C	0.82306658882388	8.26932341702115	9.76452014934360
C	1.19679223751866	8.46488841757385	11.05145740432341
H	0.61310789553387	9.12005436325731	11.69610877502470
C	-0.32432505165336	8.93406882376823	9.23103585356514
C	2.44730280256971	9.74802364990402	13.37731356482156
C	2.76354673805531	11.11299518795307	13.32284168348996
H	3.49226997625825	11.47216052064302	12.59358586059533
C	2.14442126975010	12.02417638613286	14.17391484184458
C	1.18461266121263	11.57518627383244	15.09906367575107
C	0.84376066716295	10.20947987130475	15.14427519285656
H	0.08361135483865	9.86838387075229	15.84664734162585
C	1.47923316738743	9.30899144391631	14.29656185474373
H	1.21022301141629	8.25231692530313	14.35213295526393
C	0.56781574576860	12.49755386899346	15.99856466086224
H	4.96201790310657	5.89390658534208	10.16877530275422
H	1.93640047071942	7.02291583852498	12.37206742652572
H	2.38702846315640	13.08526149439395	14.12232869018148
H	4.92311367007361	9.33052101057071	17.55911740560198
H	3.51725787258465	9.98007423147914	16.65113669763186
H	6.22330071650439	11.37488214337983	16.54759895231371
H	7.50462963792346	11.64428269632387	14.09815171996166
H	7.05824955217144	10.73112066362313	11.83659355211931
H	7.56603508144498	9.05001309736407	12.18899662145858
H	2.42960615591074	3.53188897267299	13.42794418294691
H	2.69050841317433	5.27946788208763	13.67733834016181
H	8.25018037635166	5.13841558105071	14.63757456111724
H	8.63580410107901	4.02729157735763	13.28507836766121
H	6.97463537388863	2.37045990541543	14.48599238169839
H	4.22318549297801	2.12666792964619	14.19784623633193

TS2

C	5.49361257633914	9.28175343869036	14.30823167799415
N	6.43746263312525	10.02954787066020	13.67090704265428
P	4.44502880195575	7.87433110091990	13.73897532267714
P	5.75529345145799	6.78868852547256	12.31036204243719
N	5.34219436749658	9.84059356617288	15.54145721254640
C	6.17984537908529	10.92458581358224	15.67457584592386
N	6.25875508125948	4.29911439551945	13.63219514839152
C	6.87201911755542	11.03851738821381	14.50412640253197
N	4.19881359045051	4.30592710520912	12.94588283879132
C	4.36634700498400	9.43926676307738	16.55744802414191
H	4.05134527934051	8.41020457635513	16.35731860138258
N	-1.86597453984130	7.70594221088782	9.91080229905940
N	0.13350579500338	13.23100714948838	16.77240360365768
C	6.96717878081255	9.82887258698924	12.31858894381504
H	6.23829805549578	9.29140707505345	11.69850089494508
C	5.29565231901617	5.10339015381645	13.08784490427056
C	4.47305634619476	3.03076068187737	13.39794842768636
C	5.76456706571284	3.02571027011613	13.83452987612838
C	2.90932441726210	4.70666587042086	12.38884676933789

H	2.95481699219761	4.73241162254615	11.29499619374743
C	7.60250989609210	4.72614281031134	14.01296323652114
H	7.91026676464305	5.54754393427013	13.35527270847583
C	4.91373596920578	7.04760834922320	10.80924313113418
C	3.50968620794645	7.26537138652958	10.59819060174007
C	2.70283501923997	8.04593402963404	11.48980568244979
C	3.41311348792079	8.90615038855258	12.50108574451078
H	4.15678382460741	9.48789374863174	11.93119295915425
C	2.86321352944082	6.72386035592434	9.45632396635644
H	3.46723098213944	6.17331784764193	8.73237754661841
C	1.49985009905351	6.83789851484025	9.25691875881215
H	1.02996378813566	6.38809962864321	8.38218619063304
C	0.70272818509778	7.52763724509315	10.19602543582563
C	1.32820839174074	8.14892693393444	11.28984439943742
H	0.71954542904499	8.75703557003762	11.95749979615964
C	-0.70835823912210	7.62493446360410	10.03362935528127
C	2.60853086682523	9.84432347547281	13.35915446487400
C	2.90182627310359	11.21534625171318	13.34698851060766
H	3.64687180004640	11.59847711691457	12.64710103573809
C	2.25533170070782	12.09757645861676	14.20810103471039
C	1.28227539798853	11.61453718311023	15.10075171841381
C	0.96098812943230	10.24346185601387	15.10426097598123
H	0.19553706619379	9.87262886158865	15.78548951467617
C	1.62820593292897	9.37358453729940	14.24985119494769
H	1.39066209539466	8.30929673180299	14.27849107632528
C	0.63867940557773	12.50665651564506	16.01228120058188
H	5.47657475415983	6.64040398460474	9.96302123955012
H	2.48987561364327	13.16162282644439	14.19226851882774
H	4.83572770987753	9.50560859251346	17.54514580985018
H	3.49225320051786	10.10005830853498	16.50902387766130
H	6.21084884188874	11.52344256658660	16.57677061226686
H	7.63348814932584	11.74665648107828	14.19988693293535
H	7.16871467850557	10.81228576280345	11.87925709481433
H	7.89408723407347	9.24378918750672	12.36191858649746
H	2.15317656859664	3.98609690454480	12.71668897447230
H	2.63880830075572	5.70652417101880	12.74792772521314
H	7.61495050476962	5.07011894260781	15.05571454886986
H	8.28986490184042	3.88079774022063	13.89871166711593
H	6.36411337542349	2.23237464014962	14.26421173208767
H	3.73500592833258	2.23829329126714	13.36743904775059

TS2′

P	-0.33971376409592	-0.65485337630714	0.70798681811289
C	0.67662751439991	0.34846575167014	1.86430036906141
C	-0.66235784101249	0.68813499019007	-0.60680551424928
N	0.23663410289985	0.62510070846731	3.12545020383189
N	1.83677336906197	1.04822860519457	1.70654197058544
C	-1.29325760272658	1.83502951163930	0.14205595014422
H	0.32432087679083	0.98873261223801	-1.00672202770190
C	1.10430506468794	1.49261307768870	3.74301366223589
C	-0.99520570558136	0.13595340593065	3.75767664249240
C	2.10804239821790	1.75714761059625	2.85419970001269
C	2.67671242895353	1.13150450359518	0.50742469183449
C	-0.62335548578677	3.06077391763624	0.24518478357825
C	-2.51754909685360	1.66591719390017	0.81035382447343
H	0.94697153347473	1.84541434481574	4.75597928891974
H	-1.40250358427558	-0.68616698372476	3.16268198238856
H	-0.76003151121344	-0.21628718223859	4.76823741859445
H	-1.72695163241927	0.95100668345381	3.80543884444418
H	2.98837382101328	2.38274500494570	2.95003615372119
H	2.41735782060849	2.02720858553376	-0.07054279938882
H	3.72497618211852	1.19655353347685	0.81859040538479
H	2.54151660327737	0.23090249702867	-0.09936755230443
C	-1.14175223205655	4.09277332974627	1.02340137033787
H	0.30448852052939	3.22237698580299	-0.30692173275260
C	-3.05141031152163	2.69111903846118	1.58329942267302
H	-3.06294032491557	0.72414575949798	0.72079560193216
C	-2.35406977115984	3.90795271477828	1.71120246837191
H	-0.62128522416196	5.04728568917261	1.09716799993493
H	-4.00738910837057	2.56399108739818	2.09101411761242
C	-2.87170742763560	4.94499154407172	2.54608707067126
N	-3.27980210205977	5.77715767381116	3.25156231751662
H	-2.82676024644066	1.76301194752065	-1.89076821680173
C	-2.46724100527628	0.85948148631175	-2.38139949286860
C	-1.40854587685404	0.15118970433944	-1.79115446630879
C	-3.02866037410619	0.47114916320663	-3.59480172487966
C	-0.90623660978904	-1.11150601239831	-2.42336903106961
C	-2.52818874125542	-0.69128726962390	-4.26586349975528

C	-4.09691694135443	1.22078183317079	-4.15740346414447
C	0.25831995499481	-1.83474521614683	-1.99465879996153
C	-1.52693856590832	-1.43573331733857	-3.71963288768845
H	-2.96205594219242	-0.97629596281017	-5.22446814677127
N	-4.97677838011109	1.83405313751686	-4.61602492010021
P	1.14068132148743	-1.84456295810549	-0.47105262201951
H	-1.15303863151082	-2.31649349065848	-4.24321626300430
C	0.76958952609576	-3.48960840865763	0.34415161777089
N	1.74471001460605	-4.23085039179874	0.94700572430304
N	-0.31647555917643	-4.30947395283815	0.24794733444226
C	1.26759862298286	-5.49043926420233	1.23610216809259
C	3.09504829197099	-3.77013336583202	1.28197753213313
C	-0.02350544531875	-5.54102013730282	0.79275071956720
C	-1.60014230195213	-3.96494026847569	-0.35724345189399
H	1.87584560712317	-6.24275963959430	1.72490617015795
H	3.37975900617278	-2.96912045263124	0.59042600856196
H	3.12378285973268	-3.39818582633254	2.31391270359155
H	3.79077841611932	-4.60938715002499	1.17664661637762
H	-0.74610384703083	-6.34869771692829	0.81820335149122
H	-1.48630012314777	-3.86769827967006	-1.44374659692370
H	-2.31594431854950	-4.76060718048693	-0.12912538212771
H	-1.96839674950028	-3.02173567016344	0.06636448564092
H	0.64884739376247	-2.56077522630289	-2.71222796810663
H	-1.83517982220236	-1.32889130900240	-1.66973072380258

7e^{2+}

P	4.33947173248725	7.99271606114416	13.83171188770885
C	5.37714568043891	9.29853909616490	14.60052932501078
C	3.50957855587764	9.01473177873585	12.44434856194889
N	5.10902491415482	9.74966413657712	15.85910404704225
N	6.39600280729257	10.07555987122073	14.13073613798428
C	2.66019594382546	10.03737545560963	13.15584009602220
H	4.30721842123629	9.54721820487088	11.90495488344395
C	5.94448652246162	10.79438459226269	16.16973074545781
C	4.05388850138130	9.26974492329328	16.76117630477571
C	6.75543293952467	10.99591257813246	15.08789872165721
C	7.04104798864389	9.98753001793296	12.81903600613646
C	2.91136227838641	11.40091975435991	12.94909770211554
C	1.66287646751470	9.66172601560377	14.07241621374836
H	5.90099951832042	11.30613897560828	17.12436540388584
H	3.77994245487277	8.24707567178129	16.48672941768162
H	4.43698334782165	9.28803708026947	17.78698738269669
H	3.17561283545723	9.92071038254116	16.67468975245334
H	7.55595957334204	11.70977705551655	14.93067933538916
H	6.33736907246751	10.27146345737193	12.02791358479771
H	7.88463209945194	10.68437014279691	12.80304474653233
H	7.41592531257177	8.97053529262632	12.65143680376119
C	2.19158622652518	12.37374664001483	13.63754483814736
H	3.66914982550495	11.71017412650871	12.22617334895439
C	0.93112396011783	10.62257286977237	14.76223873666853
H	1.44960405197479	8.60614485664324	14.25037653100254
C	1.19988009720897	11.98920374320621	14.55715980875180
H	2.38611466949010	13.43215736854191	13.46653182254741
H	0.14828531877152	10.32797140424042	15.46093088859776
C	0.47875519200337	12.97809393649783	15.29383317571658
N	-0.09368036198821	13.78067391974666	15.91418841594357
H	0.82298051153534	8.67635197924746	11.86725018100803
C	1.47107631742517	7.98749777666558	11.32849573676483
C	2.85507656876368	8.04388109928691	11.49042783736002
C	0.89049519054013	7.05563748069116	10.45165763136087
C	3.67852971763338	7.15897232290293	10.75191784077126
C	1.70805620985077	6.17922956987178	9.71633130326159
C	-0.53155137285151	7.00338329387446	10.32421598067735
C	5.16625970561095	7.18705544034472	10.91311955136101
C	3.08938661978028	6.24148643420164	9.87176312475505
H	1.25650568793361	5.46921685592435	9.02406198647737
N	-1.69281375075121	6.95611331197896	10.24823916722382
P	5.81546408585761	6.82851913079781	12.64615289153951
H	3.72459666680250	5.57500533386174	9.28570975577746
C	5.27744634593359	5.08845190269021	12.91639405028452
N	6.22416299985252	4.09994575351522	12.91741880975791
N	4.08349695548631	4.46210185598458	13.13994054103556
C	5.62955760538906	2.88060662233370	13.12506947200419
C	7.66708388707033	4.25944543074379	12.68837349129897
C	4.29013115805293	3.10855352880542	13.26994900277866
C	2.74107994871905	5.05141841015949	13.21772571878676
H	6.19665950559059	1.95734452803914	13.15804706014959
H	7.97793013770536	5.26062488007084	13.00229168092586

H	8.19683599199813	3.50656320203949	13.28129356292197
H	7.89622019208484	4.11721763377666	11.62474669785219
H	3.47523482749774	2.41913363894975	13.45895890981802
H	2.35156415570207	5.23881677802104	12.21113523475672
H	2.09222863711742	4.34024221562581	13.73851780037444
H	2.77718932821137	5.98386536891106	13.78920835744945
H	5.59303440107758	8.17489296490338	10.67350239978939
H	5.65795742724067	6.46946781618752	10.24155411509618

TS3

P	-1.10915426692521	-0.68159371245375	0.07015353065815
C	-0.60187329474634	0.12170567088706	1.55404492753970
P	1.21302195201160	-0.52472750110116	-0.27157377347481
C	0.61422125242335	0.63787988802589	-1.45212882614643
H	-0.39104536746826	1.19295689321716	1.55753901340209
H	0.31497664670716	1.63976003081053	-1.13772767670795
C	-0.41614447138940	-0.56960387753226	2.79547634089160
C	-0.80278140954980	-1.92006485545210	2.99618242439222
C	0.21653710019761	0.11104799410115	3.86868626757509
C	-0.52745454765541	-2.57076789742475	4.18524973774422
H	-1.33299309675205	-2.45623259248825	2.20634690719502
C	0.48576212914922	-0.53153960481656	5.06586327448585
H	0.48596735424610	1.16262210035427	3.75088867052438
C	0.13401426539229	-1.88819679547990	5.23167163786345
H	-0.82790355767490	-3.60812644232756	4.33105631017963
H	0.96120221524582	0.00213146497035	5.88867617478242
C	0.47128930929840	0.33600090400873	-2.84592517386205
C	0.96539249994924	-0.85641544370122	-3.43554651951595
C	-0.22846995956189	1.25124940119966	-3.67514734998121
C	0.73070069706461	-1.14259578769644	-4.76820126654233
H	1.54750904247230	-1.55823875077275	-2.83473029652903
C	-0.45783304135839	0.97572900513843	-5.01306373992623
H	-0.58293996459589	2.19351869093226	-3.25228247383625
C	0.00309221480694	-0.23546168464237	-5.57189136489007
H	1.11399785358509	-2.06073153656046	-5.21287897427574
H	-0.98594115918477	1.68962422238072	-5.64498219503852
C	0.43793236696353	-2.56137618604368	6.44929442759266
N	0.70801645879071	-3.11441650641186	7.43964460836222
C	-0.25806120198169	-0.53648807826775	-6.93912729720110
N	-0.49237804473703	-0.78813081751047	-8.05328776368234
C	3.71296553877615	0.46625384687638	2.66996675594126
C	3.66434850290511	1.69840688495357	2.07720179289449
H	4.27730274447795	0.11744665614985	3.52741842247566
H	4.19109413779838	2.61545612389615	2.31636023588111
C	-3.74083755992851	0.91869752260168	-2.46087694972366
C	-3.79122090293481	1.91361351082837	-1.52300363585748
H	-4.28948559879874	0.79470637134587	-3.38774136192414
H	-4.40239424603959	2.80808600376686	-1.47862704793431
C	2.30280420613363	0.33725563418538	0.93208299349686
C	-2.29245179097991	0.39976338581268	-0.83057726729033
N	2.88145780514344	-0.35922032273103	1.95565876627293
N	-2.82602754955738	-0.00506576773183	-2.02170696277904
N	2.79430436901731	1.60979917064278	1.01442075105982
N	-2.89736762512131	1.58753704241136	-0.52869325690753
C	2.71094689759709	-1.78641004499688	2.25889849477847
H	2.20927767672141	-1.89470070893818	3.22719689355379
H	2.10433777390643	-2.25584161719715	1.47879657633670
H	3.69805387658620	-2.26245902854482	2.28971615598487
C	2.51949124716044	2.73086555137869	0.10784258032820
H	2.65952059362838	2.42199922996476	-0.93250843369064
H	1.49924192805565	3.10305026693465	0.25948024907530
H	3.22575607786344	3.53390750598686	0.33993079908034
C	-2.70665882261125	2.40354166121340	0.67623025070958
H	-2.77215190986980	1.77899797508695	1.57207739175565
H	-1.73850926933814	2.91754021394554	0.64031644379676
H	-3.50307971919137	3.15352506196496	0.70520464083988
C	-2.53606089541294	-1.25359026380113	-2.73875513050376
H	-3.47837513682356	-1.78221802493202	-2.92456811339622
H	-2.04072199972398	-1.02135884248556	-3.68850685785526
H	-1.88020409984659	-1.88165284517184	-2.12864334289271

syn-$(2e)_2^{2+}$

N	7.32287061357971	7.00814437790663	3.51286151646811
P	9.57493497373969	8.04272934610090	4.78338364536337
C	8.15651604752867	6.89433703164748	4.58447572518805
N	7.58665523089521	5.98634627844488	5.42221775764864
C	6.25400416489780	6.15327560917891	3.66382462198972
C	6.41480407152853	5.51875409021202	4.86232236804575

N	10.69935931297193	9.62430158716192	12.60523024187007
C	7.50222512450819	7.92345774749802	2.37919504676933
H	8.07273203307216	7.43773463529830	1.57938885819715
C	8.07672186606304	5.56694734387866	6.73956785556063
H	8.44192025621270	6.43299693216902	7.30070375036230
C	10.75798119829960	7.36791574346855	6.09434306735481
H	10.80079723439070	6.26967041677293	6.05034614609728
C	10.70016965848746	7.87093398542969	7.49409672006049
C	10.87687261340593	6.97100053270345	8.55868349366366
H	11.02587638835620	5.90793152664553	8.35458849447818
C	10.87292095657939	7.41549278058349	9.87758449595657
H	11.00791085441811	6.71421399155340	10.70069499497139
C	10.69106384850372	8.78317054016438	10.15255801639528
C	10.50857651735979	9.69065751029333	9.09103948275373
H	10.36272640542033	10.74841316876436	9.30827909477991
C	10.51429882193599	9.23431798612743	7.77785946919130
H	10.37424166346377	9.94927237900465	6.96449933211485
C	10.69236143636716	9.24767868592176	11.50301859702844
N	14.36872329630384	7.00956141386074	6.18842131120323
P	12.11627259553583	8.04304272490510	4.91799467387440
C	13.53495292677512	6.89497009722246	5.11697104094331
N	14.10461400252581	5.98616213334951	4.27996965682821
C	15.43737372761953	6.15431910817660	6.03815900140149
C	15.27639961148165	5.51882210085040	4.84019892896868
N	10.99396535522263	9.62559144587700	-2.90361603827536
C	14.18956513715763	7.92569461860966	7.32147098603759
H	13.61947681911754	7.44039201426291	8.12182825407666
C	13.61462068740494	5.56606232876154	2.96281243789275
H	13.24898218758163	6.43177107205018	2.40144069481306
C	10.93334274095034	7.36815313040881	3.60691026607512
H	10.89079236694914	6.26988402534051	3.65065625144145
C	10.99139052156503	7.87140938499166	2.20724514768229
C	10.81558102611706	6.97157093314185	1.14242534775745
H	10.66686463079426	5.90841057129386	1.34628061116783
C	10.81998333760774	7.41627165395581	-0.17640682081208
H	10.68565810814073	6.71505708684465	-0.99968011250074
C	11.00139414277119	8.78406525363119	-0.45108107338326
C	11.18293293011181	9.69147099419007	0.61066895198159
H	11.32840003754786	10.74932967064450	0.39367108194342
C	11.17676246645467	9.23492408649731	1.92377478341749
H	11.31609041371133	9.94982022510274	2.73731492558760
C	11.00057500509504	9.24878606234425	-1.80146529773352
H	5.46886513893053	6.07232596081696	2.92078534832318
H	5.79105886422672	4.78721958890970	5.36309226497614
H	16.22253764308211	6.07383704359738	6.78122282108462
H	15.90002179957382	4.78681786129808	4.33995770864513
H	7.24132498813026	5.11998899656065	7.28735409268346
H	8.87230897671077	4.81966078816763	6.63119291311383
H	6.51461397366074	8.21719224298204	2.00883622114188
H	8.03707563956564	8.81990795716892	2.71917672246989
H	15.17723900479410	8.21997380421924	7.69122352913773
H	13.65428186101932	8.82168957152512	6.98099278056361
H	14.45017363124246	5.11932325237650	2.41507940110777
H	12.81939308253391	4.81843224913390	3.07151676405456

Structures of Figure S97

TS (blue)

N	7.42703145908426	7.42807642192256	3.15822490885927
P	9.30811585161514	7.93936857541816	5.17386051671706
C	7.86581799267628	7.10517921154373	4.40783253162710
N	6.91619628683108	6.28946354917338	4.94400778647040
C	6.22939482887087	6.79629435373464	2.90130004565743
C	5.90950800068684	6.08606197944790	4.02082866453574
N	12.41119649013877	8.99385284396361	12.42746154021941
C	8.10021291545197	8.34299264230590	2.23926453856874
H	8.88080305695087	7.81681719446969	1.67523648321442
C	6.86850745680974	5.71492904552263	6.29670680435150
H	7.33124740168133	6.40314470223187	7.00908842991840
C	9.78267198258141	6.85224015768745	6.48099423991222
H	9.65677739165348	5.77323889242300	6.37367783089053
C	10.28205258631834	7.33173603243010	7.73646583590090
C	10.75413826689732	6.39602168018226	8.69504002832665
H	10.70552663772753	5.32949955117364	8.46935671136369
C	11.28741265912353	6.81495507315845	9.90027748397763
H	11.64643062995190	6.08913105473415	10.62948095318659
C	11.37415169158988	8.19413019742520	10.19320610112534
C	10.89494480391654	9.13862002299571	9.26020411947503
H	10.93802362122710	10.19980409957699	9.50396015152362

C	10.35298895137283	8.71321797979389	8.05977925345546
H	9.94849163020598	9.45403169953722	7.36700952972800
C	11.93768741183616	8.63169290981191	11.42510201331256
N	12.38797611791621	9.04976798392251	2.40112499372590
P	10.99849129152649	6.91902351475136	3.85508849784524
C	11.80142240692081	7.81928474468445	2.43021520449859
N	11.86342037703722	7.34270377946222	1.15293196709140
C	12.82661729061099	9.32651766282518	1.12717393292413
C	12.49788029817798	8.25422670183630	0.34224349890977
N	18.78846770707830	5.53641391804553	4.65575891970492
C	12.57586675965234	9.95367042336904	3.53993644841472
H	13.49376614761325	9.69219764460911	4.08064356984289
C	11.40403380383473	6.02439017937949	0.70214544190651
H	10.91026917535027	5.51463026913328	1.53610187983115
C	12.19430146127067	7.22406864383131	5.12216252858417
H	11.92609135012937	7.82853758594451	5.98559671501915
C	13.54323375104120	6.79399710309827	5.04722719468862
C	14.48843821226202	7.31630349395532	5.97756861766086
H	14.14237735958394	7.99005407933322	6.76319432847182
C	15.82968146167145	6.99699305968618	5.88501176776712
H	16.54977855209753	7.40384000164567	6.59445785392810
C	16.27685569495840	6.13150167607058	4.86083400876122
C	15.34972218508218	5.57032028360150	3.95170223732579
H	15.70355250942750	4.86749918287429	3.19791034166505
C	14.00915124774440	5.88490490612605	4.05553659139010
H	13.28807775204397	5.38673053704346	3.40422451152411
C	17.65691673219185	5.80173817310058	4.75494733957090
H	5.70069174117296	6.91274052558049	1.96243420128380
H	5.04719227907556	5.46735013896678	4.24062506544458
H	13.33040432720021	10.25324052117926	0.87599016398159
H	12.67109720524757	8.07395329448059	-0.71291959736300
H	5.81660688486509	5.57022564389844	6.56370757234608
H	7.38487137299495	4.74789454965774	6.31835212668376
H	7.36092697555828	8.74898209670519	1.54128264120945
H	8.54266751969292	9.17044355030933	2.80786380873137
H	12.65241904172464	10.97731819146013	3.15889963878163
H	11.71743610505295	9.87986316401499	4.21467145997450
H	10.70323660259095	6.14413048126249	-0.13266877509768
H	12.26439182439873	5.43025144348913	0.37031751065282

Final Product (blue)

N	7.15886824320420	6.78682061408670	3.50585579455291
P	9.33096834688124	8.09193797818418	4.80929001912818
C	7.86696050088667	7.00060061995608	4.64673231651266
N	7.16854921782097	6.41293945835732	5.65341798316226
C	6.03223289387538	6.04225805307947	3.79302740591390
C	6.03838124621314	5.80987529816566	5.13753678566799
N	11.61976539791741	9.17357460561023	12.43356603022547
C	7.51659345390168	7.28502260138792	2.17778056202662
H	8.36529010598005	6.71595315029162	1.77691178230713
C	7.51276709207407	6.42550917335241	7.08049493751077
H	7.99524847041043	7.37355492572309	7.34167753324550
C	10.52599559229711	7.12271681973367	5.94155889667920
H	10.26240972699998	6.05455745107863	5.91152355322573
C	10.69720021364267	7.58384979398582	7.35603695255956
C	11.09220812439627	6.65187075716592	8.32890734150250
H	11.22239195441629	5.60277750240144	8.05668612049447
C	11.33800892367073	7.05431331491109	9.63752473892521
H	11.64801283430188	6.33042870429574	10.39063142098069
C	11.18296008631341	8.40639028449454	9.99528863870425
C	10.78374441175096	9.34490759438536	9.02609171452417
H	10.65873456858326	10.38953629824566	9.30939644892049
C	10.54967880110217	8.93094656918652	7.71870624293309
H	10.23183947516402	9.66906344805342	6.97818484031109
C	11.42432894352600	8.82695437763306	11.33888749925431
N	11.76915276138757	9.76302044252316	2.42888768474768
P	10.83742899792074	7.14413303602284	3.40525735497384
C	11.51626319957224	8.42793599009911	2.28551982088856
N	11.96551894313729	8.09380569443853	1.03727386867123
C	12.36610406430482	10.24680675153968	1.28898001436577
C	12.49230714392152	9.19978956341914	0.41791324640076
N	16.97946005051349	2.98064182198490	6.32982696009706
C	11.51448062240013	10.59727393513762	3.60817114840141
H	12.37295876359628	10.55741546070703	4.29075625923080
C	11.95973481686454	6.75750758695360	0.42472691502629
H	11.36665543919603	6.07832666216257	1.04443587359924
C	11.78899826992539	7.32867734418122	5.03692776512126
H	12.12789253639045	8.36625622597380	5.16531319731195

C	12.92459274670842	6.38426428369431	5.27031638096511
C	14.12191584404175	6.86188302811446	5.82255210297577
H	14.23216560231808	7.92408170663450	6.04889183441450
C	15.17145779943432	5.99201257092966	6.10272701354708
H	16.09947263205209	6.36408260417711	6.53567618844974
C	15.03401465017461	4.61993017655157	5.82719107417340
C	13.83534138073222	4.13335935551296	5.27084055423252
H	13.73591594286970	3.06956379394285	5.05734870422722
C	12.79424901494567	5.01253405212187	4.99866376090267
H	11.87140076108158	4.62149885244134	4.56302489023542
C	16.10677843621393	3.71898724806226	6.10620628511302
H	5.32316051037668	5.74826600163747	3.02786579623959
H	5.33365229364673	5.27711580991440	5.76560845713754
H	12.65038982627175	11.28796475762882	1.18404948191968
H	12.91245721213586	9.16007366807790	-0.58089968349559
H	6.58862099124008	6.32591084082647	7.65857111031453
H	8.18307234829660	5.59156142408317	7.31972877210292
H	6.65404633420973	7.16196583252805	1.51561734131074
H	7.76761718716819	8.35163258480558	2.23557589107420
H	11.36694690220980	11.63024994298376	3.27639517995793
H	10.60783534072031	10.25449630951250	4.11596569740884
H	11.52382701768333	6.82622731285004	-0.57842132703080
H	12.98858483100729	6.38327790405963	0.35781317571950
